# Cerebrospinal fluid dynamics coupled to the global circulation in holistic setting: Mathematical models, numerical methods and applications

**DOI:** 10.1002/cnm.3532

**Published:** 2021-10-19

**Authors:** Eleuterio Francisco Toro, Morena Celant, Qinghui Zhang, Christian Contarino, Nivedita Agarwal, Andreas Linninger, Lucas Omar Müller

**Affiliations:** ^1^ Laboratory of Applied Mathematics, DICAM University of Trento Trento Italy; ^2^ Department of Mathematics University of Trento Trento Italy; ^3^ Rovereto Hospital Italy; ^4^ Department of Bioengineering University of Illinois at Chicago Chicago Illinois USA

**Keywords:** advanced numerical methods, cerebrospinal fluid, circulatory system, cranio‐spinal fluid interaction, mathematical modeling modeling, neurological disorders

## Abstract

This paper presents a mathematical model of the global, arterio‐venous circulation in the entire human body, coupled to a refined description of the cerebrospinal fluid (CSF) dynamics in the craniospinal cavity. The present model represents a substantially revised version of the original Müller‐Toro mathematical model. It includes one‐dimensional (1D), non‐linear systems of partial differential equations for 323 major blood vessels and 85 zero‐dimensional, differential‐algebraic systems for the remaining components. Highlights include the myogenic mechanism of cerebral blood regulation; refined vasculature for the inner ear, the brainstem and the cerebellum; and viscoelastic, rather than purely elastic, models for all blood vessels, arterial and venous. The derived 1D parabolic systems of partial differential equations for all major vessels are approximated by hyperbolic systems with stiff source terms following a relaxation approach. A major novelty of this paper is the coupling of the circulation, as described, to a refined description of the CSF dynamics in the craniospinal cavity, following Linninger et al. The numerical solution methodology employed to approximate the hyperbolic non‐linear systems of partial differential equations with stiff source terms is based on the Arbitrary DERivative Riemann problem finite volume framework, supplemented with a well‐balanced formulation, and a local time stepping procedure. The full model is validated through comparison of computational results against published data and bespoke MRI measurements. Then we present two medical applications: (i) transverse sinus stenoses and their relation to Idiopathic Intracranial Hypertension; and (ii) extra‐cranial venous strictures and their impact in the inner ear circulation, and its implications for Ménière's disease.

## Graphical abstract

This paper presents a mathematical model of the global, arterio‐venous circulation in the entire human body, coupled to a refined description of the cerebrospinal fluid dynamics in the craniospinal cavity. The improvements concern physiological aspects, underlying mathematical models as well the associated computational methods. A major novelty of this paper is the coupling of the circulation to a refined description of the cerebrospinal fluid dynamics in the craniospinal cavity; other highlights include the myogenic mechanism of cerebral blood regulation, refined vasculature for the inner ear, the brainstem and the cerebellum, viscoelastic models for all blood vessels, arterial and venous.

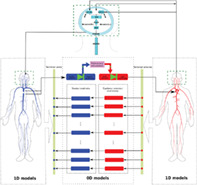

## 1 INTRODUCTION

The living human body is a complex biological, mechanical, electrical and chemical system, which involves the dynamic interaction of fluids, gases and solids, all mediated through controls, membranes and intricate networks of conduits and barriers. In this broad scenario, it is becoming increasingly accepted that bodily fluid systems and their interactive dynamics play a major role in human‐body physiology and pathology.^1^, ^2^, ^3^ A central part is played by the cardiovascular system, or circulatory system, composed of the heart, the blood and two blood vessel networks for arteries and veins connected through the microvasculature. The circulatory system, comprising both the systemic circulation and the pulmonary circulation,^1^, ^2^, ^3^ is primarily a transport system that connects muscles and organs of the full body, whereby the transporter, the flowing blood, carries nutrients, wastes, oxygen, carbon dioxide, hormones, heat, antibodies, glucose, aminoacids, fatty acids, vitamins, drugs, water and many more materials.

The functions of the cardiovascular system are accomplished through the contribution of body organs, such as the liver, the kidneys, the lungs and the central nervous system, among others. Transport of oxygen and carbon dioxide is a function that involves the respiratory system and the pulmonary circulation. The blood also transports nutrients to tissues and organs and this involves the digestive system. A major part of nutrients comes from the small intestine; here, nutrients are absorbed by capillaries into the blood stream, are transported back to the heart via the venous system and then into the arterial system that will deliver such nutrients at the level of the microvasculature. A closely related function is the clearance, or wash out, of metabolic waste products, which involves the venous system, the urinary system and the lymphatic system. This is a one‐way fluid and molecule transport system consisting of an intricate network of vessels, lymph nodes and lymph organs, such as the spleen, the thymus and the tonsils. Recall that blood in the cardiovascular system travels from the heart down to the capillaries, where blood plasma and solutes are extravasated into the interstitium, that is, into the interstitial fluid (ISF) compartment. Some of these materials are reabsorbed back directly into the circulation in the post‐capillary venules, back into the great veins and ultimately into the right atrium in the heart. The lymphatic system, starting at the initial lymphatics, collects the excess fluid and proteins from the interstitial space, called lymph once inside the lymphatic vessel, and transports these materials to the blood circulation again, primarily into the great veins in the neck and back to the heart. Transported in lymph are also proteins, waste products, interstitial macromolecules and immune cells; it is also noted that the lymphatic system is also a major transport route for disseminating tumor cells; it is also a privileged route for administered drugs; the lympahtic system plays a major role in theimmune system. Small molecules in the interstitium tend to be reabsorbed by blood capillaries/venules, whereas the lymphatic system tends to go for the larger molecules, typically of radius between 10nm and 100nm. Maintenance of tissue‐fluid balance is the primary function of the lymphatic system. All contents of the lymphatic network end up in the venous system and hence into the circulatory system. See reviews References 4 and 5.

Neurophysiology and neuropathologies draw more fluid compartments into the discussion. The cerebrospinal fluid (CSF) in the craniospinal cavity is the centre of the attention of many specialists concerned with a range of disorders of the central nervous system (CNS), such as hydrocephalus, syringomyelia, spinal cord injury, Chiari malformations, spinal tumors, to name but a few; see reviews References 6 and 7. Then, the relatively recent discovery of the so called *glymphatic system* has added strength to the biophysical point of view of neurological disorders.^8^ Evidence has been reported on its role on CNS clearance, particularly important in Alzheimers disease, which is strongly linked to the misaccumulation of amyloid beta, a protein said to be cleared by the glymphatic system. To these developments, one must add the recent discoveries of a meningeal lymphatic system by Aspelund et al^9^ and, independently, by Louveau et al^10^ the same year. The discovery of these fluid systems is contributing to configure a more complete picture of all and interconnected fluid systems acting on the CNS.

Surprisingly, the venous system,^11^ even if known for centuries as part of the circulatory system, has received much less attention than its courterpart, the arterial system. The work of Zamboni et al^12^ on the potential connection of multiple sclerosis to anomalies of the extracranial venous system has, on the one hand, resurfaced the vascular theory of multiple sclerosis, and on the other, has also stimulated increasing attention to the venous district of the circulatory system and its potential link to several neurological pathologies. As is known, the main routes for venous return from the CNS to the heart are the internal jugular veins, the vertebral veins and the azygous vein.^13^ It has become established that such venous draining routes may be affected by various types of anomalies, such as stenoses, resulting in impaired draining of venous blood from the CNS to the heart. Several venous‐system associated pathologies of unknown cause have been reported in recent years. Examples include retinal abnormalities,^14^ transient global amnesia,^15^, ^16^ transient monocular blindness,^17^, ^18^ Ménière's disease,^19^, ^20^, ^21^, ^22^ idiopathic Parkinson's disease,^23^ and many more. For a review, see Reference 24 and the many references therein.

Covid‐19 entered the world scene in the year 2020 and has spread to every corner of the world causing, so far, the death of more than 1 million people, as well as social and economic havoc, globally. Early scientific communications,^25^, ^26^, ^27^ identified Covid‐19 patients as suffering from hypoxemic respiratory failure, coagulopathy, evidence of ischemia in the lower limbs and cerebral infarcts in multiple vascular territories. Subsequente communications have confirmed the systemic character of Covid‐19, revealing the involvement of not only the respiratory system, but also of the circulatory system as a whole and the CNS.^28^ Covid‐19, perhaps more than any other disease, along with its global consequences, has also revealed the need for a global approach to the study of the human bodily fluid systems and related pathologies.

Mathematical modeling and simulation in many areas of science and technology is a success story in the last few decades. Developments have not gone unnoticed to cardiovascular mathematicians, who have made significant advances in the biomedical field. For a description of the state‐of‐the‐art in cardiovascular mathematics see for instance Reference 29 and references therein. Early contributions include the seminal work of Otto Frank in 1899_,_ ^30^ which was primarily concerned with the basic shape of the arterial pulse. This is probably the first application of a mathematical model to successfully describe haemodynamics, especially the exponential decay of the arterial pressure pulse in diastole. Another early, seminal contribution is due to Guyton in the early 70s,^31^ which among other aspects, was concerned with system analysis of arterial pressure regulation and hypertension, incorporating detailed regulatory mechanisms. The review by Shi^32^ is highly recommended. Over the last few decades, most of the mathematical modeling effort has gone into the arterial system, isolated from other fluid compartments, notably the venous system. A remarkable early attempt to model all intracranial vascular compartments is reported in Reference 33. Considering the cardiovascular system from a wider point of view, that is not considering exclusively cerebral blood flow (CBF), significant advances have been reported in recent years, see for example,^34^, ^44^ to name but a few. For venous flow, however, there is consensus in the cardiovascular modeling community that the subject is largely open. As a matter of fact, as already pointed out, research on the venous system as a whole has not been given the importance it deserves, also from the point of view of physiology. Early contributions on CSF dynamics are due to Ursino.^45^ ^,^ ^46^ Mathematical models of CSF dynamics with clinical relevance in normal and pathological conditions were pioneered by Linninger and coworkers.^47^, ^48^, ^49^ For more recent developments on the coupling of CSF mechanics and cerebral vasculature see Reference 50. The lymphatic system is also beginning to receive attention from mathematical modelers, although most works have considered the system in isolation. An early attempt to model the lymphatic system is due to Reddy.^51^ More recent works are due to Bertram et al,^52^ Caulk et al^53^ and Contarino and Toro,^54^ to name but a few. A major problem here, as for the rest of the human cardiovascular system, is the lack of data for model development and validation.

The most complete mathematical model for the human extracellular bodily fluids will include the complete circulatory system (arteries, veins, microvasculature), ISF, the lymphatic system and the CSF. The mathematical modelsshould recognize that most conduits are compliant, giving rise to free‐boundary problems, dealt with in the framework of fluid–structure interaction (FSI), involving time‐dependent, non‐linear systems of equations for the fluid and the vessel wall mechanics in three space dimensions.^29^ ^,^ ^55^ ^,^ ^56^ FSI models have the advantage of resolving local details such as rheology, velocity vectors and wall shear stresses. However, due to their complexity and computational cost, at the present time, it is unrealistic to think of deploying FSI models for a full human bodily fluid configuration, not even for the circulatory system alone. One‐dimensional (1D) averaged models derived from the full 3D models offer a realistic alternative, still retaining the fluid–structure interaction but in a simpler, computationally tractable setting. Cross‐sectional area, cross‐sectional averages of velocity, flow and pressure at any time and position along the length of vessels can be computed, at a much lower computational cost. Yet, these 1D models cannot be deployed for the full vessel network and one must still consider even simpler models, zero‐dimensional (0D) or compartmental or lumped parameter models.^45^ These are governed by systems of ordinary differential equations (ODEs) in time, subject to algebraic constraints. In view of the complexities alluded to, a realistic and popular approach is the geometric multi‐scale approach, whereby the human circulation, for example, can be represented by a combination of 3D FSI models, 1D averaged models and lumped parameter, or 0D models, with appropriate matching conditions.^29^, ^57^ One of the early works following this approach, in which both the arterial and venous systems are coupled in the model is that of Liang.^58^ More recently, Müller and Toro in References 59, 60 put forward a global, closed‐loop mathematical model, with sub‐models for the heart, the arterial system, the venous system, the microvasculature, the pulmonary circulation and a very simple model for the CSF; notably, this model included a sophisticated representation of the venous system, especially for the head and neck. Subsequent works along these lines include that of Mynard.^61^

**FIGURE 1:**
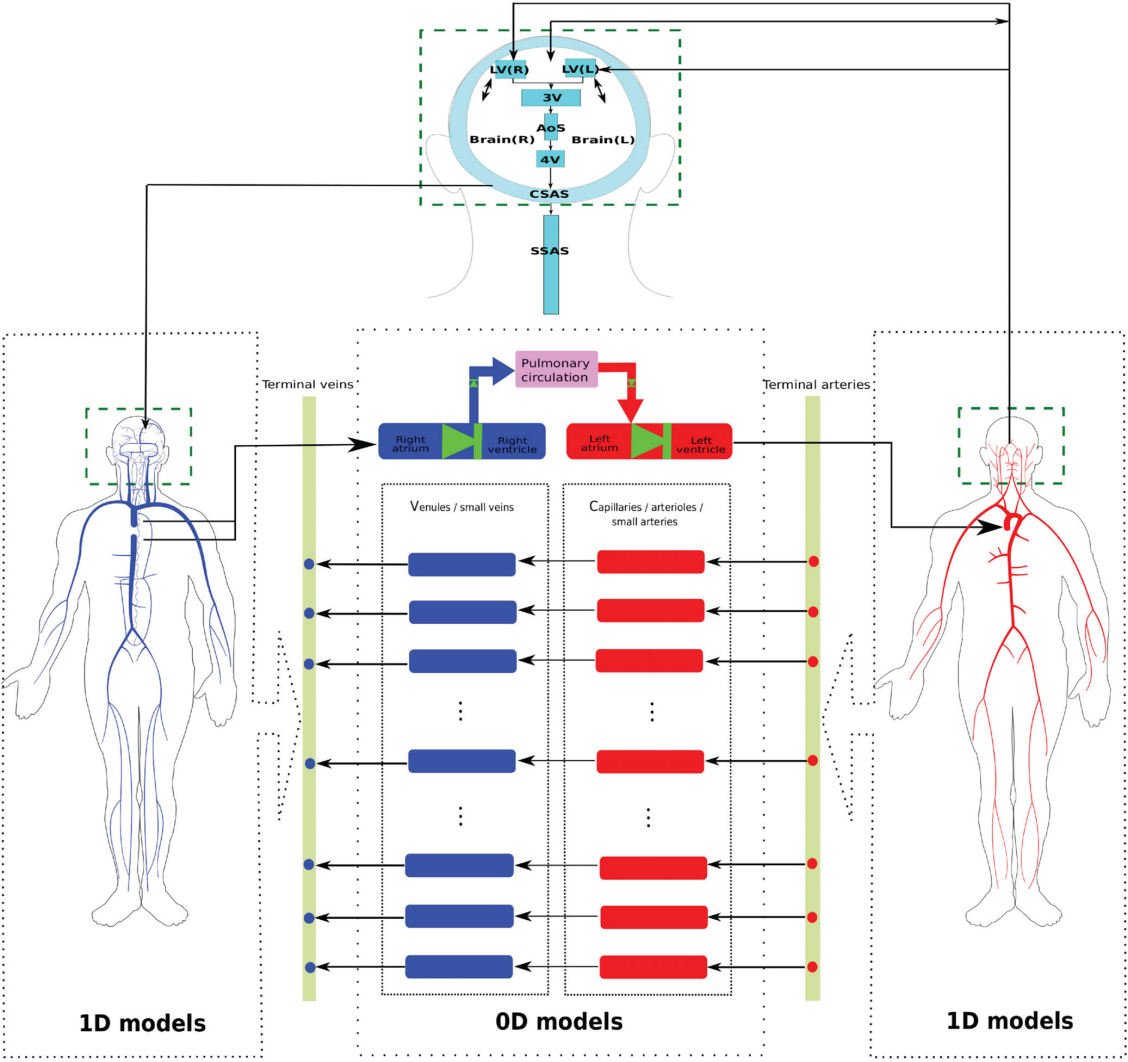
Schematic representation of the global model for the full human circulatory system coupled to the craniospinal fluids and brain parenchyma. The arterial 1D network is represented in the right dotted box with red vessels while the venous 1D network is displayed in the left dotted box with blue vessels. The terminal arteries of the arterial network are connected to draining veins of the venous circulation through 0D models representing arterioles, capillaries, small arteries (red boxes) and venules, small veins (blue boxes). The dotted arrows indicate the connection between 1D network and terminal vessels, depicted for simplicity as dots in the yellow bar. Left and right cardiac chambers are displayed by red and blue boxes, respectively, connected to green atrioventricular valves; the left ventricle is connected to aortic root and venae cavae are linked to right atrium through black arrows. Cardiac chambers are connected to the pulmonary circulation, comprising arteries, capillaries and veins, represented by the pink box, through the aortic and pulmonary valves (green arrows). The CSF compartments are represented by cyan boxes. The arrows between cardiovascular system and CSF circulation represent the fluid exchange between these systems through production and absorption while the green dashed boxes indicate the mechanical interaction between between all components in the cranial cavity through the Monro‐Kellie hypothesis

The present paper results from the amalgamation of two substantial and independently developed pieces of work and attempts to incorporate the major extracellular fluid compartments of the human body. The first piece consists of a substantially improved version of the original Müller–Toro mathematical model^59^ for the global systemic and pulmonary circulations in the entire human body. The improvements concern physiological aspects, underlying mathematical models as well the associated computational methods. Then, the second piece of work, a major novelty of this paper, is the coupling of the circulation as described, to a refined mathematical description of the CSF dynamics in the craniospinal cavity, building upon the model proposed by Linninger.^62^ This includes all major CSF pathways and the brain parenchyma, accounting for deformations and interaction between the cerebral vasculature, brain parenchyma and CSF compartments during the cardiac cycle. The present mathematical model is depicted in Figure 1. Major fluid components are the arterial system (right) and the venous system (left) for the entire body, comprising 323 major blood vessels. The craniospinal cavity, in addition to the vasculature and the two‐phase brain parenchyma, contains CSF represented by 0D compartments for the cranial subarachnoid space (CSAS), the four cerebral ventricles, the aqueduct of Sylvius (AoS) and the spinal subarachnoid space (SSAS). Additional components include the four heart chambers, cardiac valves, three compartments for the pulmonary circulation, 31 compartmental models describing the connections between terminal arteries and veins through the microcirculation, 17 venous valves, 21 Starling resistors (SR). The potential medical applications of the resulting model are numerous. Here, we have chosen to illustrate the applicability of our model to two classes of fluid‐dynamics related pathologies that involve the close dynamical interaction of all major fluid compartments in the craniospinal space. The first class of pathologies concerns transverse sinus stenoses and its relation to idiopathic intracranial hypertension (IIH).^63^, ^64^, ^65^ The second class of fluid‐related pathologies concerns the altered haemodynamics of the inner ear circulation resulting from extra‐cranial venous outflow strictures, and its implications for Ménière's disease.^20^, ^66^, ^67^, ^68^

Even if the resulting model is suitable for the study of several physiological and pathophysiological phenomena, it must be noted that its application for patient‐specific simulations would be complex. Indeed, it is characterized by many parameters that are difficult to be measured in the clinic, in particular for the CSF part. Thus, while the main goal of this model is to explore fundamental aspects of physiological and pathological states, an attempt toward a patient‐specific application was done with a previous version of the Müller‐Toro model.^59^ In that work, major head and neck veins were modified according to patient‐specific MRI‐derived geometrical information.

The rest of the paper is structured as follows. Section 2 presents the mathematical models; section 3 presents the numerical methods to solve the equations; section 4 deals with parametrization of the model; section 5 shows sample computations and validation of results against published data and MRI measurements. In section 6, we illustrate the potential applicability of the full model to fluid‐dynamics related pathologies. Discussion and conclusions are found in section 7.

## 2 MATHEMATICAL MODELS

In this section, we present the mathematical models for describing the human circulatory system and the CSF dynamics in the craniospinal space. As already pointed out, the present paper is based on two independently developed pieces of work. The circulatory system part emerges from References 59 and 69, with some significant improvements. For the 1D representation of the blood vessels we include viscoelasticity of the vessel wall.^39^, ^70^ The resulting partial differential equation system is solved numerically using the high‐order Arbitrary DERivative Riemann problem (ADER) framework^71^ with a solver that allows for local time stepping (LTS).^72^ The microcirculation and heart models are also partially modified with respect to Reference 59. Representation of pulmonary circulation follows the same models as in References 59 and 69 wherein a simple three‐compartment (arteries, capillaries, veins) description for systemic microcirculation based on the work by Sun et al^73^ is adopted. Venous valves and SR are modeled following.^41^ The second piece of work underpinning this paper is the coupling of the circulation to a refined mathematical description of the CSF dynamics in the craniospinal cavity, for which we follow the model proposed by Linninger.^62^ The Linninger model^63^ was chosen because it integrated results of extensive MR imaging studies^47^, ^48^, ^49^ with detailed two and 3D mathematical models into a comprehensive mathematical description of the major intracranial dynamics with fluid structure interactions of blood, CSF in the ventricular system, as well as cranial and the SSASs and the deformable brain parenchyma.

Since in this article we are particularly interested in the cerebral dynamics, we incorporate into our model one of the most important physiological control systems, the cerebral autoregulation. This process aims at maintaining adequate and stable CBF during changes in blood pressure working on dilatation or contraction of arterioles and capillaries.^77^ The model used to account for this phenomenon is based on References 75 and 76.

### 2.1 Equations for blood flow in major vessels

In this section, we review the partial differential equations representing blood flow in major vessels, along with closure laws and reformulations.

#### 2.1.1 Conservation laws and closure conditions

The flow of blood in major arteries and veins is represented through 1D cross‐sectional averaged models resulting in time‐dependent systems of partial differential equations. We start from the classical laws of conservation of mass and of balance of momentum

(1) $$\left\{\begin{array}{l} \partial_{t}A+\partial_{x}q=0\;,\\ \partial_{t}q+\partial_{x}\left(\hat{\alpha }\frac{q^{2}}{A}\right)+\frac{A}{\rho }\partial_{x}p=-f\;. \end{array}\right.$$

For details on the derivation of (1) see Reference 29, for example. The 2×2 system (1) contains three unknowns, namely $A\left(x,t\right)$, the cross‐sectional area of the vessel lumen; $q\left(x,t\right)$, the blood flow rate and $p\left(x,t\right)$, the cross‐sectionally averaged internal pressure. Parameters in the equations include $\hat{\alpha }$, the Coriolis coefficient, ρ the blood density, assumed constant, and f the friction force per unit length of the tube. The Coriolis coefficient depends on the assumed velocity profile; here we take $\hat{\alpha }=1$, which corresponds to an assumed parabolic velocity profile.

The system of differential Equations (1) has more unknowns than equations; hence, one extra closure condition is required. Such extra condition, usually called *tube law*, attempts to couple the internal blood flow distribution with the mechanical properties of the solid moving vessel wall. A comparative analysis of various mathematical descriptions of elastic properties of vessel walls in modern 1D models of hemodynamics can be found in Reference 77. In the existing versions of our model,^59^, ^69^ we used elastic tube laws for both arteries and veins. In the present paper, we improved upon this by adopting viscoelastic tube laws for both arteries and veins in the entire circulation. To this end, we follow recent works concerned with approximating time‐dependent parabolic systems with hyperbolic balance laws with stiff source terms,^78^, ^79^ The approach was applied in Reference 70 to a simplified arterial network. In the present paper, we deploy the framework to the full human circulatory system, major arteries and veins. Following Reference 62, the following pressure‐area relation (tube law) is adopted

(2) $$p\left(x,t\right)=\psi \left(A\left(x,t\right);A_{0}\left(x\right),K\left(x\right),P_{0}\right)+\varphi \left(A\left(x,t\right);A_{0}\left(x\right)\right)\partial_{t}A+p_{\mathrm{ext}}\left(x,t\right).$$

Here, the first term $\psi \left(A\left(x,t\right);A_{0}\left(x\right),K\left(x\right),P_{0}\right)$ is the elastic part of the tube law, which in turn depends on the reference pressure $P_{0}$ and the parameters $A_{0}\left(x\right)$ and $K\left(x\right)$ and can be written as

(3) $$\psi \left(A\left(x,t\right);A_{0}\left(x\right),K\left(x\right),P_{0}\right)=K\left(x\right)\Phi \left(A\left(x,t\right),A_{0}\left(x\right)\right)+P_{0},$$

with

(4) $$\Phi \left(A\left(x,t\right);A_{0}\left(x\right)\right)=\left({\left(\frac{A\left(x,t\right)}{A_{0}\left(x\right)}\right)}^{m}-{\left(\frac{A\left(x,t\right)}{A_{0}\left(x\right)}\right)}^{n}\right).$$

The term $\varphi \left(A\left(x,t\right);A_{0}\left(x\right)\right)\partial_{t}A$ represents the viscoelastic part of the tube law, while $p_{\mathrm{ext}}\left(x,t\right)$ denotes the external pressure. As usual, the transmural pressure is defined as

(5) $$p_{\text{transm}}\equiv p\left(x,t\right)-p_{\mathrm{ext}}\left(x,t\right)=K\left(x\right)\Phi \left(A\left(x,t\right),A_{0}\left(x\right)\right)+P_{0}+\varphi \left(A\left(x,t\right);A_{0}\left(x\right)\right)\partial_{t}A.$$

We now define geometric and mechanical parameters in the tube law. $A_{0}\left(x\right)$ defines the vessel cross‐sectional area at equilibrium; $K\left(x\right)$ represents the vessel wall stiffness, wile m and n are two real numbers, to be specified. Note that $A_{0}\left(x\right)$ and $K\left(x\right)$ are variable parameters, they depend on distance x along the vessel.Throughout this work, we assume m=0.5 and n=0 for arteries, while m=10 and n=−1.5 for veins. Moreover, $K\left(x\right)$ is a positive function that was obtained from the reference wave speed $c_{0}$ assumed for each vessel, distinguishing arteries, veins and dural sinuses.

The viscoelastic term of the tube law depends on the time partial derivative of the cross‐sectional area of the vessel and is defined as

(6) $$\varphi \left(A\left(x,t\right);A_{0}\left(x\right)\right)\partial_{t}A=\frac{\Gamma }{A_{0}\sqrt{A}}\partial_{t}A.$$

Γ is related to the viscoelastic properties of the vessel wall, which following^39^ is chosen as

(7) $$\Gamma =\frac{2}{3}\sqrt{\pi }\gamma h_{0}\left(x\right),$$

where γ is the *wall viscosity*. The wall viscosity is evaluated as the product of the viscoelastic parameter $K_{M}$ and the volume fraction of smooth muscle. $K_{M}$ is chosen such that hysteresis behavior of pressure‐area plots in peripheral arteries and veins lies within the physiological range. For arteries we take $K_{M}=3\times 10^{5}$ dyn s/cm^2^ and a percentage of smooth muscle of 10%.^80^ For veins we take $K_{M}=5\times 10^{4}$ dyn s/cm^2^ and a smooth muscle fraction of 8%. Concerning the wall thickness $h_{0}\left(x\right)$ we follow^80^ and express it in relation to the vessel radius at equilibrium. For arteries $h_{0}=10\%r_{0}$, while for veins $h_{0}=5\%r_{0}$.

The momentum equation in (1) contains the *friction term* $f\left(x,t\right)$, which depends on the local velocity profile (assumed a priori). Here, we take

(8) $$f=\frac{8\mu \pi }{\rho }\frac{q}{A},$$

with μ being the blood *dynamic viscosity*.

#### 2.1.2 Variable material properties and augmented equations

As already pointed out, the material and geometric parameters $K\left(x\right)$, $A_{0}\left(x\right)$ and $p_{\mathrm{ext}}\left(x,t\right)$ are in general functions of distance x along the vessel length. Computationally, in order to deal with this situation we adopt the variable‐parameter formulation of Toro and Siviglia,^81^ admitting now, viscoelastic tube laws for arteries and veins.^70^ System 1, along with the tube law (2), is then written as the following extended 5×5 system

(9) $$\{\begin{array}{c} \partial_{t}A+\partial_{x}q=0,\\ \partial_{t}q+\partial_{x}\left(\alpha \frac{q^{2}}{A}\right)=-\frac{A}{\rho }\partial_{x}p_{ext}-\frac{A}{\rho }\Phi \partial_{x}K-\frac{A}{\rho }\left(K\partial_{A}\Phi -\partial_{A}\varphi \partial_{x}q\right)\partial_{x}A\\ -\frac{A}{\rho }\left(K\partial_{A_{0}}\Phi -\partial_{A_{0}}\varphi \partial_{x}q\right)\partial_{x}A_{0}+\frac{A}{\rho }\varphi \partial_{x}^{\left(2\right)}q-f,\\ \partial_{t}K=0,\\ \partial_{t}A_{0}=0,\\ \partial_{t}p_{ext}=0\;. \end{array}$$

In succinct form system (9) reads

(10) $$\partial_{t}Q+A\left(Q\right)\partial_{x}Q=\partial_{x}G\left(Q;\partial_{x}Q\right)+S\left(Q\right),$$

where Q is the vector of unknowns

(11) $$Q={\left[A\;q\;K\;A_{0}\;p_{\mathrm{ext}}\right]}^{T},$$

$A\left(Q\right)$ is the coefficient matrix

(12) $$A\left(Q\right)=\left[\begin{array}{ccccc} 0 & 1 & 0 & 0 & 0\\ c^{2}-u^{2}+\frac{\varphi \partial_{x}q}{2\rho } & 2u & \frac{A}{\rho }\Phi & \frac{A}{A_{0}}\left(\frac{\varphi \partial_{x}q}{\rho }-c^{2}\right) & \frac{A}{\rho }\\ 0 & 0 & 0 & 0 & 0\\ 0 & 0 & 0 & 0 & 0\\ 0 & 0 & 0 & 0 & 0 \end{array}\right],$$

$S\left(Q\right)$ is the source term vector

(13) $$S\left(Q\right)={\left[\begin{array}{ccccc} 0 & -f & 0 & 0 & 0 \end{array}\right]}^{T}\;$$

and

(14) $$\partial_{x}G\left(Q;\partial_{x}Q\right)={\left[\begin{array}{ccccc} 0 & \frac{\mathrm{\varphi A}}{\rho }\partial_{x}^{\left(2\right)}q & 0 & 0 & 0 \end{array}\right]}^{T}\;$$

is a higher‐order differential term emanating from the viscoelastic part of the tube law. This last differential term in the advection–diffusion–reaction system defines a parabolic problem, no longer hyperbolic, as in the case of a purely elastic tube law.^81^

#### 2.1.3 Hyperbolic approximation of a parabolic system

Toro and Montecinos,^78^, ^79^ proposed a method to approximate time‐dependent parabolic problems by hyperbolic systems with stiffsource terms, by extending the Cattaneo relaxation approach.^82^ Montecinos and coworkers^70^ applied the procedure to a network of arterial blood vessels, thereby setting the bases for its extension to the global, closed‐loop circulation model of this paper, including arteries and veins.

To start with, a new variable ζ and a relaxation parameter ɛ>0 are introduced such that

(15) $$\zeta \to \partial_{x}q\;\mathrm{as}\;ɛ\to 0.$$

One then replaces the spatial gradient of the flow variable in (9) by the new variable ζ in (15), which is constrained to satisfy an additional evolutionary PDE, namely the constitutive Cattaneo's law, which reads

(16) $$\partial_{t}\zeta =\frac{1}{ɛ}\left(\partial_{x}q-\zeta \right).$$

We now have an augmented 6×6 system. Keeping the same notation for the vector of unknowns, the coefficient matrix and the source term we may write

(17) $$\partial_{t}Q+A\left(Q\right)\partial_{x}Q=S\left(Q\right),$$

with

(18) $$Q={\left[\begin{array}{cccccc} A & q & K & A_{0} & p_{\mathrm{ext}} & \zeta \end{array}\right]}^{T},$$

(19) $$A\left(Q\right)=\left[\begin{array}{cccccc} 0 & 1 & 0 & 0 & 0 & 0\\ c^{2}-u^{2}+\frac{a_{\Gamma }}{2} & 2u & \frac{A}{\rho }\Phi & \frac{A}{A_{0}}\left(a_{\Gamma }-c^{2}\right) & \frac{A}{\rho } & -\frac{A}{\rho }\varphi \\ 0 & 0 & 0 & 0 & 0 & 0\\ 0 & 0 & 0 & 0 & 0 & 0\\ 0 & -\frac{1}{ɛ} & 0 & 0 & 0 & 0 \end{array}\right],$$

(20) $$S={\left[\begin{array}{cccccc} 0 & -f & 0 & 0 & 0 & -\frac{1}{ɛ}\zeta \end{array}\right]}^{T},$$

(21) $$c^{2}=\frac{A}{\rho }K\partial_{A}\Phi ,\;u=\frac{q}{A},\;a_{\Gamma }=\frac{\varphi \zeta }{\rho }.$$

Here, c is the wave velocity (analogous to the sound speed) associated to theelastic tube law. The 6×6 system (17) with the state vector (18) and coefficient matrix (19) is hyperbolic,^70^, ^78^ provided the relaxationparameter is chosen so as to satisfy

(22) $${ɛ}^{-1}\geq -\frac{\zeta }{2A}-\frac{\rho c^{2}}{\mathrm{\varphi A}}.$$

All eigenvalues of the coefficient matrix (19) are real and given as

(23) $$\lambda_{1}=u-\tilde{c}\;,\;\lambda_{2}=\lambda_{3}=\lambda_{4}=\lambda_{5}=0\;,\;\lambda_{6}=u+\tilde{c}\;,$$

where now $\tilde{c}$ denotes the*wave speed* associated to the complete tube law.

(24) $$\tilde{c}=\sqrt{c^{2}+\omega },\;\omega =\frac{\mathrm{\varphi A}}{\rho ɛ}+\frac{a_{\Gamma }}{2}.$$

The corresponding right eigenvectors are

(25) $$\begin{array}{l} R_{1}={\left[\begin{array}{cccccc} 1 & u-\tilde{c} & 0 & 0 & 0 & -\frac{1}{\int } \end{array}\right]}^{T},\\ R_{2}={\left[\begin{array}{cccccc} 1 & 0 & 0 & 0 & 0 & \frac{c^{2}+a_{\Gamma }/2-u^{2}}{\varphi A}\rho \end{array}\right]}^{T},\\ R_{3}={\left[\begin{array}{cccccc} 0 & 0 & 1 & 0 & 0 & \frac{\Phi }{\varphi } \end{array}\right]}^{T},\\ R_{4}={\left[\begin{array}{cccccc} 0 & 0 & 0 & 1 & 0 & \frac{\left(a_{\Gamma }-c^{2}\right)}{\varphi A_{0}}\rho \end{array}\right]}^{T},\\ R_{5}={\left[\begin{array}{cccccc} 0 & 0 & 0 & 0 & 1 & \frac{1}{\varphi } \end{array}\right]}^{T},\\ R_{6}={\left[\begin{array}{cccccc} 1 & u+\tilde{c} & 0 & 0 & 0 & -\frac{1}{\int } \end{array}\right]}^{T}. \end{array}\}$$

These eigenvectors associated to the real eigenvalues (23) can be shown to be linearly independent, and hence system (17) is hyperbolic.

We now consider two fundamental properties of system (17), namely the nature of the characteristic fields and the generalized Riemann invariants. It can be shown that the characteristic fields associated to eigenvectors $R_{1}$ and $R_{6}$ are *genuinely non‐linear*, while the remaining characteristic fields are *linearly degenerate*. The generalized Riemann invariants associated to $R_{1}$ and $R_{6}$ are

(26) $$\Gamma_{1}=u+\int \frac{\tilde{c}}{A}\mathrm{dA}=\text{constant},$$

(27) $$\Gamma_{6}=u-\int \frac{\tilde{c}}{A}\mathrm{dA}=\text{constant},$$

respectively. We also note that for constant $p_{\mathrm{ext}},A_{0}$ and K, the generalized Riemann invariants for the linearly degenerate fields associated with $R_{2}$, $R_{3}$, $R_{4}$ and $R_{5}$ are

(28) $$\Gamma_{1}^{\mathrm{LD}}=\tilde{p}+\frac{1}{2}\rho u^{2}=\text{constant}\;$$

and

(29) $$\Gamma_{2}^{\mathrm{LD}}=q=\text{constant},$$

where

(30) $$\tilde{p}=p_{\mathrm{ext}}+\psi -\varphi \zeta \;.$$

More details about the hyperbolic reformulation of the problem and its eigenstructure are found in References 79, 70. Next, we present the 0D mathematical models, which consist of systems of ODEs.

### 2.2 Equations for lumped‐parameter models

In the previous section, we described mathematical models for major arterial and venous blood vessels, consisting of systems of partial differential equations. Here, we present compartmental, or 0D, models consisting of systems of ODEs, for other districts of the circulation. These include the microvasculature (arterioles, capillaries and venules/veins), the heart, the pulmonary circulation, venous valves and SR. We also present a mathematical model for cerebral autoregulation, which works on the terminal portion of the cerebral arteries and on the cerebral vascular beds. Lumped‐parameter models for CSF compartments^62^ will be introduced in section 2.3.

#### 2.2.1 The microvasculature

**FIGURE 2:**
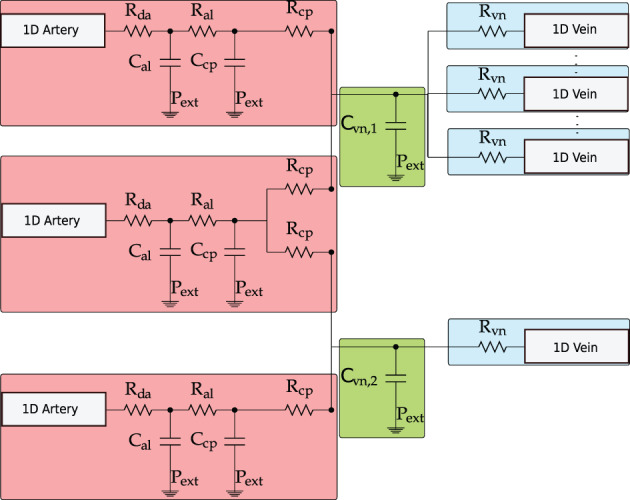
Schematic representation of the generic vascular bed model. The red boxes include the terminal 1D arteries and the corresponding arterioles and capillaries compartments; the green boxes represent the venous capacitors while the light blue boxes refer to terminal venules/1D veins

Physiologically, the arterial system is connected to the venous system through arterioles, capillaries and venules. To describe this connection, the microvasculature is represented by lumped‐parameter, or 0D, models. This connection can be simple, such as between one artery and one vein, or entail numerous compartments. The generic vascular bed model for all microvasculature beds, depicted in Figure 2, is inspired in the three‐element Windkessel model. The model is characterized by

- Characteristic impedances. These couple any number of connecting 1D arteries/veins to lumped‐parameter models for the microvasculature ($R_{\mathrm{da}}$ or $R_{\mathrm{vn}}$) and regulate the pressure drop between 1D domains and vascular beds;
- Peripheral resistances and compliances divided into arterioles $\left(R_{\mathrm{al}},C_{\mathrm{al}}\right)$ and capillaries $\left(R_{\mathrm{cp}},C_{\mathrm{cp}}\right)$;
- Venous compartment with related compliance $C_{\mathrm{vn}}$.

As illustrated in Figure 2, each connecting artery can be linked to one or both venous capacitors, while each venous capacitor can be connected to any number of terminal veins. Note that the second artery splits into both venous capacitors, while the other arteries supply only one of them. Moreover, any number of veins can be connected to each venous capacitor.

For each vascular bed, the variables to be computed are pressure and flow, denoted as follows: for arterioles $P_{\mathrm{al}}$, $Q_{\mathrm{al}}$; for capillaries $P_{\mathrm{cp}}$, $Q_{\mathrm{cp}}$ and for venules $P_{\mathrm{vn}}$ and $Q_{\mathrm{vn}}$. Thus, for each element of the vascular bed one has

(31) $$\left\{\begin{array}{l} \frac{\mathrm{dP}}{\mathrm{dt}}=\frac{1}{C}\left(Q_{\text{in}}-Q\right)+\frac{{\mathrm{dP}}_{\mathrm{ext}}}{\mathrm{dt}},\\ Q=\frac{P-P_{\text{out}}}{R}, \end{array}\right.$$

where C is compliance and $P_{\mathrm{ext}}$ is the external pressure, generally taken as zero (relative to atmospheric pressure) or equal to the intracranial pressure in the case of intracranial peripheral beds. $Q_{\text{in}}$ and $P_{\text{out}}$ are flow and pressure in neighboring compartments or obtained from the 1D models through boundary conditions. Well‐matched coupling to the connecting 1D arterial and venous segments is achieved via the characteristic impedances, as suggested in Reference 83.

#### 2.2.2 Valves, Starling resistors and stenosis

Here, we describe a valve model based on Reference 41 that predicts valve motion on the basis of the instantaneous difference between upstream and downstream pressures. In the present paper, the model is applied to describe cardiac valves, venous valves, SR in the cerebral circulation and stenosis. Here, we illustrate the general model, while for each specific application, more details are given in sections 2.2.3, 2.2.4 and 6.

The valve dynamics is described by means of the function $A_{e}\left(t\right)$ defining the effective cross‐sectional area, expressed by

(32) $$A_{e}\left(t\right)=A_{a}\left[M_{s}\xi \left(t\right)+M_{r}\left(1-\xi \left(t\right)\right)\right],$$

where $A_{a}$ is the annulus area and the time‐dependent function $\xi \left(t\right)$ is the valve state, a dimensionless number in the range $\left[0,1\right]$ and representing the rate of opening and closing of the valve. $\xi \left(t\right)$ is given by the solution of the following variable‐coefficient ODEs

(33) $$\left\{\begin{array}{ll} \frac{\mathrm{d\xi}}{\mathrm{dt}}=K_{o}\left(\Delta p\left(t\right)-\Delta p_{o}\right)\left(1-\xi \left(t\right)\right) & \text{if}\;\Delta p>\Delta p_{o}\;\text{opening state},\\ \frac{\mathrm{d\xi}}{\mathrm{dt}}=K_{c}\left(\Delta p\left(t\right)-\Delta p_{c}\right)\xi \left(t\right) & \text{if}\;\Delta p<\Delta p_{c}\;\text{closing state}. \end{array}\right.$$

Here, $K_{o}$ and $K_{c}$ are rate coefficients for opening and closing respectively; $\Delta p_{o}$ and $\Delta p_{c}$ are opening and closure threshold pressures. In (32), $M_{r}$ and $M_{s}$ are parameters representing regurgitating and stenotic valves respectively. Specifically, a healthy valve corresponds to $M_{r}=0$ and $M_{s}=1$, an incompetent valve is described by $M_{r}>0$, while a stenotic valve is represented by $M_{s}<1$. Flow variation in time across the valve is given by a first‐order, variable coefficients, ODE

(34) $$\frac{\mathrm{dq}\left(t\right)}{\mathrm{dt}}=\frac{1}{L\left(t\right)}\left(\Delta p\left(t\right)-B\left(t\right)q\left(t\right)|q\left(t\right)|\;\right).$$

$\Delta p\left(t\right)$ is the pressure difference across the valve length, defined as

(35) $$\Delta p\left(t\right)=p_{\mathrm{up}}\left(t\right)-p_{\text{down}}\left(t\right)\;,$$

where $p_{\mathrm{up}}\left(t\right)$ and $p_{\text{down}}\left(t\right)$ are the upstream and downstream pressures at time *t*, with respect to valve direction. $p_{\mathrm{up}}\left(t\right)$ and $p_{\text{down}}\left(t\right)$ are evaluated from other compartments of the global model, specified later. $L\left(t\right)$ and $B\left(t\right)$ are time‐dependent coefficients; $L\left(t\right)$ is blood inertance, which accounts for the component of the pressure difference related to blood acceleration; $B\left(t\right)$ is the Bernoulli's resistance, which governs pressure differences related to convective acceleration and dynamic pressure losses due to diverging flow. They are expressed by

(36) $$L\left(t\right)=\frac{\rho l_{e}}{A_{e}\left(t\right)},$$

and

(37) $$B\left(t\right)=\frac{\rho }{2A_{e}^{2}\left(t\right)}.$$

Here, ρ is the constant blood density and $l_{e}$ is the effective length. More details on the valve model are found in References 41, 59, 75 and in the appendix of Reference 84.

#### 2.2.3 Heart and pulmonary circulation

In the present paper, we consider a heart model for the dynamics of the four chambers and the cardiac valves. The chambers are denoted as $\mathrm{ch}=\left\{\mathrm{RA},\mathrm{RV},\mathrm{LA},\mathrm{LV}\right\}$, where RA and RV are the right atrium and ventricle, while LA and LV represent the left atrium and ventricle. For the chambers we follow the*time‐varying elastance* model in References 58, 73, while cardiac valves are modeled as presented in section 2.2.2 following.^41^ Briefly, blood pressure in each cardiac chamber is calculated as

(38) $$P_{\mathrm{ch}}=P_{\text{peri}}+E\left(t\right)\left(V_{\mathrm{ch}}-V_{\mathrm{ch},0}\right)+\gamma P_{\mathrm{ch}}\frac{{\mathrm{dV}}_{\mathrm{ch}}}{\mathrm{dt}},$$

where $V_{\mathrm{ch}}$ is the current cardiac volume; $V_{\mathrm{ch},0}$ is the dead volume (assumed to be 0); $\gamma P_{\mathrm{ch}}\left(t\right)$ is the viscoelasticity coefficient of the cardiac wall and $P_{\text{peri}}\left(t\right)$ is the external pericardial pressure defined by

(39) $$P_{\text{peri}}=\exp\left(\frac{V_{H}-V_{\mathrm{PC}}}{\Phi_{\mathrm{PC}}}\right),$$

where $V_{H}\left(t\right)$ is the sum of the volume of each heart chamber and $V_{\mathrm{PC}}$, $\Phi_{\mathrm{PC}}$ are constant parameters. $P_{\mathrm{ext}}\left(t\right)$ is the external pericardial pressure and $E\left(t\right)$ in (38) is the time‐varying elastance, defined as

(40) $$E\left(t\right)=E_{A}e\left(t\right)+E_{B},$$

where the constants $E_{A}$ and $E_{B}$ are the active and passive elastances, respectively, while $e\left(t\right)$ is the normalized time‐varying elastance, taken as in Reference 73 as

(41) $$e(t)\equiv e_{a}(t)=\{\begin{array}{cc} \frac{1}{2}\{1+\cos\left[\pi (t+T-t_{ar})/T_{arp}\right]\}, & 0\leq t\leq t_{ar}+T_{arp}-T,\\ 0, & t_{ar}+T_{arp}-T<t\leq t_{ac},\\ \frac{1}{2}\{1-\cos\left[\pi (t-t_{ac})/T_{acp}\right]\}, & t_{ac}<t\leq t_{ac}+T_{acp},\\ \frac{1}{2}\{1+\cos\left[\pi (t-t_{ar})/T_{arp}\right]\}, & t_{ac}+T_{acp}<t\leq T, \end{array}$$

for atria and as

(42) $$e\left(t\right)\equiv e_{v}\left(t\right)=\left\{\begin{array}{ll} \frac{1}{2}\left[1-\cos\left(\mathrm{\pi t}/T_{\mathrm{vcp}}\right)\right], & 0\leq t\leq T_{\mathrm{vcp}},\\ \frac{1}{2}\left\{1+\cos\left[\pi \left(t-T_{\mathrm{vcp}}\right)/T_{\mathrm{vrp}}\right]\right\}, & T_{\mathrm{vcp}}<t\leq T_{\mathrm{vcp}}+T_{\mathrm{vrp}},\\ 0, & T_{\mathrm{vcp}}+T_{\mathrm{vrp}}<t\leq T, \end{array}\right.$$

for ventricles.

The modeling of cardiac valves is described in what follows. As each chamber of the heart contracts, blood is pushed through a valve either into another chamber or out of the heart into an artery (aorta or pulmonary). The four cardiac valves (tricuspid, pulmonary, mitral and aortic) ensure one‐way blood flow by (a) opening to let blood through and (b) closing to prevent backflow. The mechanism that leads to opening or closure of a valve is driven by blood pressure changes as the heart contracts and relaxes. Such mechanism is modeled here following section 2.2.2. The pressure drop across each cardiac‐valve length is defined from pressure data from the neighboring cardiac chamber, from the aortic root and from the pulmonary arterial compartment. In particular, the tricuspid and mitral valves are atrioventricular valves that prevent backflow of blood from the ventricles into the atria; in these cases, the upstream pressure is the pressure of the atrium while the downstream pressure is that of the ventricle. The pulmonary valve is located at the opening between the right ventricle and the pulmonary trunk, therefore its upstream and downstream pressures are the right ventricle and the arterial pulmonary pressure, respectively. Finally, the pressure drop across the aortic valve is determined by the difference between the left ventricle and the aortic root pressure. In Equation (33), $\Delta p_{o}$ and $\Delta p_{c}$ are set to 0 for all the cardiac valves. Other parameters are defined later in section 4.1.

#### 2.2.4 Venous valves and Starling resistors

The Müller–Toro global model^59^, ^69^ is equipped with submodels for venous valves and SR consisting of ideal diodes. In the present article, these elements are replaced with the model described in section 2.2.2.

##### Venous valves

Venous valves are placed at different locations of the venous network; each venous valve is located between two venous vessels and governs flow across this interface. Location of valves are reported in Table 5 and are related to the venous network depicted in Figures 5 and 6. In this case, the pressure drop which governs the flow rate across the valve is determined by the pressure difference between the upstream and downstream vessels with respect to the valve direction. Moreover, the annulus area $A_{a}$ is assumed to be equal to the mean area between the reference areas of the connecting vessels on the right and left side. In the same way, the effective length $l_{e}$ is taken as the mean diameter at equilibrium between the upstream and downstream vessels. Finally, $\Delta p_{o}$ and $\Delta p_{c}$ are set to 0.

##### Starling resistors

Venous CBF is ensured by a SR mechanism, a fluid dynamic construct which governs the flow in collapsible tubes exposed to variable external pressure. The SR act at the confluence of cortical veins in the dural sinuses; these are located in the dura mater and are more rigid than cerebral veins. During the large physiological fluctuations of the intracranial pressure, the SR mechanism prevents the vein collapse maintaining the blood pressure upstream the collapsed segment higher than the intracranial pressure.

In previous work, we described SR simply via ideal diodes.^69^ Here, we adopt a model that allows for a richer description of opening/closing dynamics, as well as accounting for a better description of underlying physical processes. In our venous network, Starling resistor behavior is represented through the model proposed in section 2.2.2. The pairs of vessels between which each SR element is placed are reported in Table 5, where the left vessel index represents the number of the cortical vein while the right vessel index indicates the corresponding venous sinus. The annulus area $A_{a}$ and the effective length $l_{e}$ are assumed to be the mean area and diameter, respectively, between the reference area and diameter at equilibrium of the pair of vessels connected to the SR element. The flow across SR is limited to that given by the pressure difference between the cerebral vein (upstream vessel) and the larger of intracranial pressure and the downstream pressure. When the intracranial pressure is much higher than the downstream pressure, the flow rate through the vessel becomes independent of the downstream pressure and the driving pressure difference is given by the upstream and the external pressures.As for other intracranial compartments, the external pressure is the intracranial pressure, that in this work is taken as the pressure in the fluid part of the brain parenchyma. Therefore, the valve state in Equation (33) is determined by $\Delta p-\Delta p_{o}=\Delta p-\Delta p_{c}=p_{\text{down}}\left(t\right)-p_{\mathrm{ext}}\left(t\right)$, where $p_{\mathrm{ext}}$ is the intracranial pressure. If $p_{\text{down}}\left(t\right)<p_{\mathrm{ext}}\left(t\right)$, in Equation (34), the driving pressure difference Δp is given by $\Delta p=p_{\mathrm{up}}-p_{\mathrm{ext}}$; on the other hand, if the downstream pressure is higher than the external pressure, the flow across the SR element is determined by the pressure difference between the upstream and downstream pressures $\Delta p=p_{\mathrm{up}}-p_{\text{down}}$. Other parameters used in the valve and SR models are reported in.

#### 2.2.5 Control system: Cerebral autoregulation

We consider a model of the cerebrovascular regulation mechanisms, which acts by modifying resistances and compliances of the arterial microcirculation; changes in these parameters are not independent but are related through biomechanical and geometrical laws. The model is based on References 75 and 76, with appropriate modifications. Only one control mechanism is considered in this work, the myogenic response, which is linked to changes in arterial pressure and CBF. The original autoregulation model proposed in Reference 75 reproduces also the metabolic response of cerebral autoregulation, which is linked to carbon dioxide reactivity and the amount of oxygen reaching the brain tissue. This mechanism is not included in the present paper, as the current version of our model does not yet include a submodel for the transport of CO_2_ and oxygen in the brain. It is an aspect to be considered in the near future.

Cerebral myogenic autoregulation is activated by changes in CBF; its action on the arterial microvasculature (arterioles and capillaries) includes a static gain G and first‐order low‐pass dynamics with the time constant τ. An increase in CBF causes vasoconstriction and, consequently, a decrease in compliance and an increase in resistance. The regulatory response is modeled by a sigmoidal static relationship with upper and lower levels to account for the limits of vasodilatation and vasoconstriction capacities.

The following differential equations describe the actions of autoregulation by means of first‐order low‐pass dynamics, time constants and gains

(43) $$\tau \frac{{\mathrm{dx}}_{i}}{\mathrm{dt}}=-x_{i}+G_{i}\left(\frac{{\bar{Q}}_{i}-{\bar{Q}}_{i}^{T}}{{\bar{Q}}_{i}^{T}}\right);$$

$x_{i}$ is the state variable of cerebral autoregulation of the *i*th cerebral terminal artery that responds to alteration of CBF. $G_{i}$ is the static gain for the *i*th cerebral terminal artery; it is evaluated from the total static gain of autoregulation G (which valued will be defined in section 4.1) according to the flow distribution inside the brain. ${\bar{Q}}_{i}$ is the time averaged flow and ${\bar{Q}}_{i}^{T}$ is the reference flow at the *i*th terminal artery over the period $\left[t-T,t\right]$, where t is the current time and T is the cardiac cycle duration.

Once the control actions $x_{i}$ of each *i*th terminal artery are found solving the ordinary differential Equation (43), arterial compliances are changed through a sigmoidal relationship.

(44) $$C_{i}=\frac{{\bar{C}}_{i}\left\{\left(1-\Delta C_{i}/2\right)+\left(1+\Delta C_{i}/2\right)\exp\left[\left(-x_{i}\right)/k_{i}\right]\right\}}{1+\exp\left[\left(-x_{i}\right)/k_{i}\right]},$$

with upper and lower saturation levels. In this section, $C_{i}$, $R_{i}$ and $V_{i}$ stand for compliance ($C_{\mathrm{al}}$, $C_{\mathrm{cp}}$), resistance ($R_{\mathrm{al}}$, $R_{\mathrm{cp}}$) or volume of arteriolar and capillaries compartments that are in the vascular beds linked to the *i*th terminal artery, $k_{i}$ is a constant parameter, inversely proportional to the central slope of the sigmoidal curve, ${\bar{C}}_{i}$ and $\Delta C_{i}$ are the central value and the amplitude of the sigmoidal curve. $\Delta C_{i}$ depends on whether vasodilation or vasoconstriction is considered and it is chosen for each terminal artery as follows

(45) $$\left\{\begin{array}{cc} \Delta C_{i}=2{\mathrm{sat}}_{1},\;k_{i}={\bar{C}}_{i}{\mathrm{sat}}_{1} & \text{if}\;x_{i}>0,\\ \Delta C_{i}=2{\mathrm{sat}}_{2},\;k_{i}={\bar{C}}_{i}{\mathrm{sat}}_{2} & \text{if}\;x_{i}<0, \end{array}\right.$$

where ${\mathrm{sat}}_{1}$ and ${\mathrm{sat}}_{2}$ are constant parameters that define the upper and lower saturation levels of the sigmoidal curve. According to the literature,^85^ the sigmoidal curve is not symmetrical; the increase in blood volume induced by vasodilation is higher than the blood volume decrease induced by vasoconstriction; therefore, two different values must be chosen for the parameter $\Delta C_{i}$. From (44) and (45), it follows that the upper and lower saturation levels of the sigmoidal curve are ${\bar{C}}_{i}+\frac{{\mathrm{sat}}_{2}}{2}$ and ${\bar{C}}_{i}-\frac{{\mathrm{sat}}_{1}}{2}$, respectively.

The cerebrovascular control mechanisms affects not only the compliances, but also the arterial resistances. The variation of compliance in time changes the arterial volume. Since blood volume in a vessel, as a first approximation, is proportional to the radius squared ($V\propto r^{2}$), for a given vessel length, and vessel resistance is proportional to the inverse of radius to the power four ($R\propto 1/r^{4}$), volume varies according to the inverse square root of the resistance ($V\propto 1/\sqrt{R}$). Therefore, the following relationship is used to update the resistances of regulating arteries

(46) $$\frac{{\bar{V}}_{i}}{{\bar{V}}_{i}^{T}}=\sqrt{\frac{{\bar{R}}_{i}^{T}}{R_{i}}},$$

where ${\bar{V}}_{i}$ is the mean volume of the *i*th arterial compartment (arterioles and capillaries) in the interval $\left[t-T,t\right]$, while ${\bar{V}}_{i}^{T}$ is the mean baseline condition volume, $R_{i}$ is the current resistance of the arteriolar‐capillaries compartment and ${\bar{R}}_{i}^{T}$ is the resistance under baseline conditions.

### 2.3 Equations for cerebrospinal fluid and brain dynamics

A major aspect of the present paper is the coupling of the circulatory system to a more refined description of the CSF dynamics than in the first version of our model.^59^, ^69^ We depart from the model proposed by Linninger and collaborators.^62^ The version of the Linninger model we present here differs somehow from the original version and includes nine CSF compartments: the lateral (LV and RV), the third (3 V) and the fourth (4 V) ventricles; the cerebral aqueduct (AoS), the CSAS, the SSAS and the bi‐phasic brain parenchyma, comprising the left and right hemispheres. Each compartment is spatially idealized as a cylinder of length l and variable cross‐sectional area $A\left(t\right)$. Each brain parenchyma hemisphere is treated as an incompressible, deformable medium composed of two phases, the solid cell matrix, representing neurons, glial cells and axon fibers (70% of its total volume), and the extracellular fluid (remaining 30%); the model assumes that the volume of the solid matrix does not change and therefore brain parenchyma size changes depend only on the extracellular fluid content variations, that is, changes in porosity. All CSF compartments are interconnected and contain CSF assumed to be a Newtonian and incompressible liquid, with a constant viscosity of $0.001\mathrm{kg}/\left(\mathrm{ms}\right)$ and a constant density of $998.2\mathrm{kg}/m^{3}$_._ ^62^

In our model, we assume that CSF is secreted by the choroid plexuses, a highly vascularized region from the microcirculation of the anterior and posterior cerebral arteries, into the lateral ventricles. Also included is a constant CSF production from arterioles to the ventricles and the diffuse capillary production throughout the brain parenchyma to the ventricles. Then, CSF flows from the lateral ventricles to the third ventricle and, through the cerebral aqueduct, to the fourth ventricle. Then CSF is assumed to enter the subarachnoid space. Here, CSF is absorbed into the venous system through arachnoid villi into the superior sagittal sinus. Moreover, from the CSAS, CSF is displaced into another CSF compartment, namely the SSAS.

**FIGURE 3:**
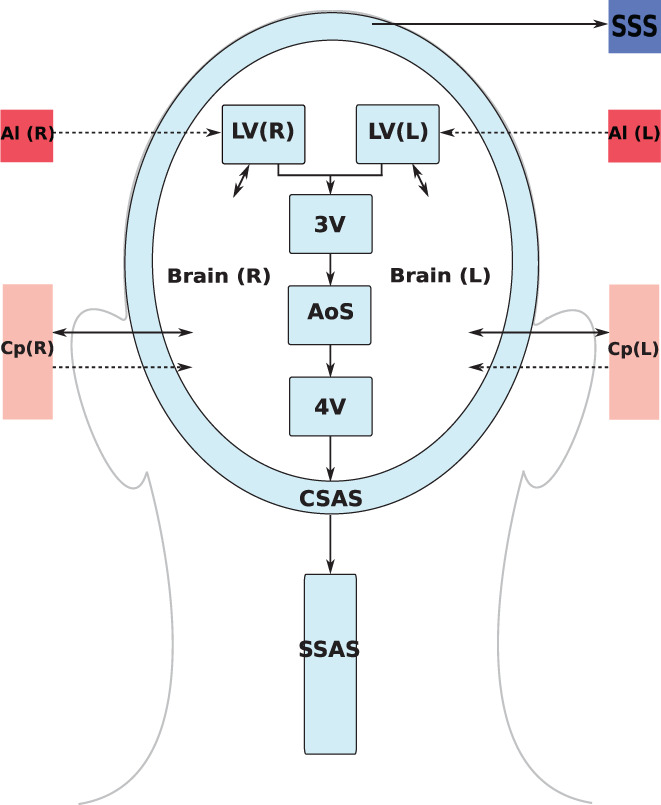
Schematic representation of the cerebrospinal fluid (CSF) compartments. RV: right lateral ventricle; LV: left lateral ventricle; 3V: third ventricle; 4V: fourth ventricle; AoS: aqueduct of Sylvius; CSAS: cranial subarachnoid space; SSAS: spinal subarachnoid space; SSS: superior saggital sinus Brain: fluid part of brain parenchyma; Al: cerebral arterioles; Cp: cerebral capillaries. Solid double arrows denote fluid exchange between different compartments driven by pressure differences, while dashed arrows describe constant CSF production. The combination of a single dashed arrow and a solid double arrow between the brain parenchyma and the capillaries indicates that there are both fluid exchange driven by pressure differences ($q_{\mathrm{br}}^{\text{in}}$ driven by $p_{\mathrm{Cp}}-p_{\mathrm{br}}$) and constant CSF production ($q_{\mathrm{Cp}\to \mathrm{br},\text{const}}$). Single solid arrow denotes CSF reabsorption into the venous system (SSS)

Figure 3 shows the craniospinal compartments involved in the CSF system and their connectivity. The arrows indicate fluid exchange between compartments driven by pressure differences, while dashed arrows denote constant production of CSF, from the cerebral arterioles into the lateral ventricles $q_{\mathrm{Al}\to \mathrm{LVs},\text{const}}$ and from the brain capillaries into the extracellular space of the parenchyma. The type of arrows identifies whether the exchange of CSF between different compartments is unidirectional or bidirectional.

Flow of CSF in CSF compartments is governed by mass conservation and momentum balance. Such equations are accompanied by a tube law, relating deformation state and pressure, as for 1D vessels. Other equations are included in the model to account for fluid exchange between different compartments of the CSF system or with the vasculature. The full CSF model is composed of 36 equations and 36 unknowns. The continuity equations read

(47) $$l_{\mathrm{RV}}\frac{{\mathrm{dA}}_{\mathrm{RV}}}{\mathrm{dt}}=q_{\mathrm{RV}}^{\text{in}}-q_{\mathrm{RV}}^{\text{out}}\;,$$

(48) $$l_{\mathrm{LV}}\frac{{\mathrm{dA}}_{\mathrm{LV}}}{\mathrm{dt}}=q_{\mathrm{LV}}^{\text{in}}-q_{\mathrm{LV}}^{\text{out}}\;,$$

(49) $$l_{3V}\frac{{\mathrm{dA}}_{3V}}{\mathrm{dt}}=q_{3V}^{\text{in}}-q_{3V}^{\text{out}}\;,$$

(50) $$l_{\mathrm{AoS}}\frac{{\mathrm{dA}}_{\mathrm{AoS}}}{\mathrm{dt}}=q_{\mathrm{AoS}}^{\text{in}}-q_{\mathrm{AoS}}^{\text{out}}\;,$$

(51) $$l_{4V}\frac{{\mathrm{dA}}_{4V}}{\mathrm{dt}}=q_{4V}^{\text{in}}-q_{4V}^{\text{out}}\;,$$

(52) $$l_{\text{CSAS}}\frac{{\mathrm{dA}}_{\text{CSAS}}}{\mathrm{dt}}=q_{\text{CSAS}}^{\text{in}}-q_{\text{CSAS}}^{\text{out}}\;,$$

(53) $$l_{\text{SSAS}}\frac{{\mathrm{dA}}_{\text{SSAS}}}{\mathrm{dt}}=q_{\text{SSAS}}^{\text{in}}-q_{\text{SSAS}}^{\text{out}}\;,$$

(54) $$l_{\mathrm{br},R}\frac{{\mathrm{dA}}_{\mathrm{br},R}}{\mathrm{dt}}=q_{\mathrm{br},R}^{\text{in}}+q_{\mathrm{Cp}\to \mathrm{br},\text{const}}-q_{\mathrm{br},R}^{\text{out}}-q_{\mathrm{br}\to \mathrm{RV},\text{const}}\;,$$

(55) $$l_{\mathrm{br},L}\frac{{\mathrm{dA}}_{\mathrm{br},L}}{\mathrm{dt}}=q_{\mathrm{br},L}^{\text{in}}+q_{\mathrm{Cp}\to \mathrm{br},\text{const}}-q_{\mathrm{br},L}^{\text{out}}-q_{\mathrm{br}\to \mathrm{LV},\text{const}}\;.$$

Equations ((47), (48), (49), (50), (51), (52), (53), (54), (55)) are continuity equations that ensure that CSF is neither gained nor lost. Equations ((47), (48), (49), (50), (51), (52), (53)) refer to continuity equations for ventricles, Aqueduct of Sylvious, cranial and SSAS. Each equation guarantees that the volume change is given by the difference between the volumetric flow rates in and out of that compartment. Equations (54) and (55) are the continuity equations for the right and left fluid part of the brain parenchyma, respectively. In this case, the right‐hand‐side of the equations considers both the volumetric flow rate in and out of the compartment that is driven by pressure differences and the constant mass transfer. Flow into the the brain parenchyma is the sum of a constant CSF production from the brain capillaries into the extracellular space of the parenchyma, $q_{\mathrm{Cp}\to \mathrm{br},\text{const}}$ and the pressure driven seepage from the capillaries to the brain parenchyma, $q_{\mathrm{br}}^{\text{in}}$. Flow exiting the brain parenchyma is the sum of a constant seepage from the extracellular space of the parenchyma into the ventricles, $q_{\mathrm{br}\to \mathrm{LV},\text{const}}$, and a pressure driven exchange between brain parenchyma and lateral ventricles $q_{\mathrm{br},L}^{\text{out}}$.

The momentum equations are effectively Darcy's law of flow and relate the pressure difference between two compartments to the volumetric flow q exchanged between them and a resistance to flow R. For the brain parenchyma compartments, there are two momentum equations, one refers to CSF exchange between the lateral ventricles and the extracellular fluid matrix of the brain, while the other one relates to the secretion of CSF from cerebral capillaries. As shown in Figure 3, these exchange pathways are bi‐directional, depending on the hydrostatic pressure differences. When intracranial pressure exceeds the capillary pressure, reverse flow occurs, that is, in the present model capillaries are a pathway for CSF drainage. The equations for CSF flow are

(56) $$q_{\mathrm{RV}}^{\text{out}}=\frac{p_{\mathrm{RV}}-p_{3V}}{R_{3V}}\;,$$

(57) $$q_{\mathrm{LV}}^{\text{out}}=\frac{p_{\mathrm{LV}}-p_{3V}}{R_{3V}}\;,$$

(58) $$q_{\mathrm{AoS}}^{\text{in}}=\frac{p_{3V}-p_{\mathrm{AoS}}}{R_{\mathrm{AoS}}}\;,$$

(59) $$q_{4V}^{\text{in}}=\frac{p_{\mathrm{AoS}}-p_{4V}}{R_{4V}}\;,$$

(60) $$q_{\text{CSAS}}^{\text{in}}=\frac{p_{4V}-p_{\text{CSAS}}}{R_{\text{CSAS}}}\;,$$

(61) $$q_{\text{SSAS}}^{\text{in}}=\frac{p_{\text{CSAS}}-p_{\text{SSAS}}}{R_{\text{SSAS}}}\;,$$

(62) $$q_{\mathrm{br},R}^{\text{in}}=\frac{p_{\mathrm{Cp},R}-p_{\mathrm{br},R}}{R_{\mathrm{br}}}\;,$$

(63) $$q_{\mathrm{br},L}^{\text{in}}=\frac{p_{\mathrm{Cp},L}-p_{\mathrm{br},L}}{R_{\mathrm{br}}}\;,$$

(64) $$q_{\mathrm{br},R}^{\text{out}}=\frac{p_{\mathrm{br},R}-p_{\mathrm{RV}}}{R_{\mathrm{br},2}}\;,$$

(65) $$q_{\mathrm{br},L}^{\text{out}}=\frac{p_{\mathrm{br},L}-p_{\mathrm{LV}}}{R_{\mathrm{br},2}}\;.$$

The notation for pressures is obvious for most compartments; for example $p_{\text{CSAS}}$ denotes pressure in the cerebral subarachnoid compartment. Just for clarity, in the last four equations $p_{\mathrm{Cp},R}$ is pressure in the capillary compartments of the right side of the brain, while $p_{\mathrm{Cp},L}$ is pressure in the capillary compartments of the left part of the brain; $p_{\mathrm{br},R}$ is pressure in the extracellular fluid part of the right brain parenchyma and $p_{\mathrm{br},L}$ is pressure in extracellular fluid part of the left brain parenchyma. We note that Equation (62) attempts to account for the interacting dynamics of two major CSF compartments. We are currently investigating these aspects as it has clearly some limitations, particularly regarding the omission of inertial terms that are known to influence the timing of flow exchange between CSAS and SSAS.^87^

As already pointed out, the distensibility equations play the role of the tube law; they relate the internal pressure with the cross‐sectional area of the compartment in a linear manner. They describe the dilation and compression of a compartment; if the pressure of the compartment exceeds the external pressure, the compartment is dilated with respect to the reference state ɛ0ɛ; in the opposite case, the compartment is compressed. For each compartment inside the cranial cavity, the external pressure is that of the brain parenchyma; for the SSAS, the external pressure is taken equal to zero. For a generic compartment z, the distensibility equation expresses pressure $p_{z}$ as a function of cross‐sectional area $A_{z}$ in the compartment and three additional parameters, namely an external pressure $p_{\mathrm{ext},z}$, baseline cross‐sectional area $A_{z}^{0}$ and a coefficient $E_{z}$ denoting elastance, that is

(66) $$p_{z}=p_{\mathrm{ext},z}+E_{z}\left(\frac{A_{z}}{A_{z}^{0}}-1\right)\;,$$

Therefore, for each specific compartment the equations are

(67) $$p_{\mathrm{RV}}=p_{\mathrm{br},R}+E_{\mathrm{RV}}\left(\frac{A_{\mathrm{RV}}}{A_{\mathrm{RV}}^{0}}-1\right)\;,$$

(68) $$p_{\mathrm{LV}}=p_{\mathrm{br},L}+E_{\mathrm{LV}}\left(\frac{A_{\mathrm{LV}}}{A_{\mathrm{LV}}^{0}}-1\right)\;,$$

(69) $$p_{3V}=\frac{1}{2}\left(p_{\mathrm{br},R}+p_{\mathrm{br},L}\right)+E_{3V}\left(\frac{A_{3V}}{A_{3V}^{0}}-1\right)\;,$$

(70) $$p_{\mathrm{AoS}}=p_{\mathrm{br}}+E_{\mathrm{AoS}}\left(\frac{A_{\mathrm{AoS}}}{A_{\mathrm{AoS}}^{0}}-1\right)\;,$$

(71) $$p_{4V}=\frac{1}{2}\left(p_{\mathrm{br},R}+p_{\mathrm{br},L}\right)+E_{4V}\left(\frac{A_{4V}}{A_{4V}^{0}}-1\right)\;,$$

(72) $$p_{\text{CSAS}}=\frac{1}{2}\left(p_{\mathrm{br},R}+p_{\mathrm{br},L}\right)+E_{\text{CSAS}}\left(\frac{A_{\text{CSAS}}}{A_{\text{CSAS}}^{0}}-1\right)\;,$$

(73) $$p_{\text{SSAS}}=E_{\text{SSAS}}\left(\frac{A_{\text{SSAS}}}{A_{\text{SSAS}}^{0}}-1\right)\;.$$

Additional equations connecting different compartments are required to complete the description of CSF flow. Specifically, for the right lateral ventricle RV, the amount of CSF that enters the right lateral ventricle RV is equal to the amount of CSF exiting the fluid part of the brain parenchyma plus the constant production rate from arterioles $q_{\mathrm{Al}\to \mathrm{RV},\text{const}}$ plus the constant production rate from capillaries $q_{\mathrm{Cp}\to \mathrm{br},\text{const}}$, namely

(74) $$q_{\mathrm{RV}}^{\text{in}}=q_{\mathrm{br},R}^{\text{out}}+q_{\mathrm{br}\to \mathrm{RV},\text{const}}+q_{\mathrm{Al}\to \mathrm{RV},\text{const}}\;.$$

Similarly for the left lateral ventricle LV,

(75) $$q_{\mathrm{LV}}^{\text{in}}=q_{\mathrm{br},L}^{\text{out}}+q_{\mathrm{br}\to \mathrm{LV},\text{const}}+q_{\mathrm{Al}\to \mathrm{LV},\text{const}}\;.$$

Then, CSF flows from lateral ventricles to the third ventricle

(76) $$q_{\mathrm{RV}}^{\text{out}}+q_{\mathrm{LV}}^{\text{out}}=q_{3V}^{\text{in}}\;,$$

from the third ventricle to the AoS

(77) $$q_{3V}^{\text{out}}=q_{\mathrm{AoS}}^{\text{in}}\;,$$

from the AoS to the fourth ventricle

(78) $$q_{\mathrm{AoS}}^{\text{out}}=q_{4V}^{\text{in}}\;,$$

and from the fourth ventricle to the CSAS

(79) $$q_{4V}^{\text{out}}=q_{\text{CSAS}}^{\text{in}}\;.$$

From the cerebral subarachnoid space CSAS, CSF is temporarily displaced into the spinal cavity and reabsorbed into the superior saggital sinus (SSS) through the arachnoid granulations.^62^, ^87^ Reabsorption is represented by a mass transfer flux, which is a function of the pressure difference between the CSAS and CSAS and a reabsorption constant coefficient k

(80) $$q_{\text{CSAS}}^{\text{out}}=q_{\text{SSAS}}^{\text{in}}+\max\left(0,k\left(p_{\text{CSAS}}-p_{\text{sinus}}\right)\right).$$

We take the maximum value between zero and the mass transfer flux to enforce the unidirectional flow from the cranial SAS to the venous sinus. Previous MRI measurements^88^ have shown that in a normal subject CSF reabsorption in the spinal cavity is negligible, since almost the total flow into the spinal SAS goes back into the cerebral SAS; for this reason, we set the CSF outflow from the SSAS equal to zero,

(81) $$q_{\text{SSAS}}^{\text{out}}=0.$$

Finally, the Monro–Kellie hypothesis is enforced: all compartments, except the SSAS, are enclosed inside the cranium and the volume of each cerebral hemisphere remains constant over time

(82) $$V_{\text{Blood},R}+V_{\mathrm{RV}}+\frac{1}{2}V_{3V}+\frac{1}{2}V_{\mathrm{AoS}}+\frac{1}{2}V_{4V}+\frac{1}{2}V_{\text{CSAS}}+V_{\mathrm{br},R}+V_{\text{Solid Parenchyma}}=\text{constant},$$

(83) $$V_{\text{Blood},L}+V_{\mathrm{LV}}+\frac{1}{2}V_{3V}+\frac{1}{2}V_{\mathrm{AoS}}+\frac{1}{2}V_{4V}+\frac{1}{2}V_{\text{CSAS}}+V_{\mathrm{br},L}+V_{\text{Solid Parenchyma}}=\text{constant}.$$

The volume of each compartment is evaluated as product of its length and its cross‐sectional area.

So far, we have presented the complete model for the circulatory system and the craniospinal dynamics. In the next section, we briefly present the numerical methods to solve all the equations.

## 3 NUMERICAL METHODS

Much of the numerical methodology utilized here has already been applied in the original Müller–Toro model for the human circulatory system.^59^ Therefore, in the present paper we limit ourselves to a succinct presentation of the numerical methods, with emphasis on the new aspects, along with relevant references.

### 3.1 Overview of methods for PDE system

The hyperbolic system of blood flow Equations (17) is solved numerically using the ADER high‐order numerical framework, originally reported in Reference 64; some of the numerous, subsequent ADER developments are found in References 89, 90, 91, 92, 93, 94, 95, 96. An up to date review of ADER is found in^97^ and references therein. An introductory presentation of the ADER methods is given in Chaps. 19 and 20 of Reference 98. A key ingredient of the ADER method is the solution of the generalized Riemann problem (GRP). In the present paper, we use the Dumbser–Enaux–Toro (DET) method,^95^ extended to nonconservative systems in References 99 and 100. This is a locally implicit method that is able todeal with stiff source terms, in particular, the stiff source terms resulting from the hyperbolic approximation,^70^, ^78^, ^79^ of the parabolic system incorporating the viscoelastic nature of the vessel wall mechanics. The initial conditions for the GRP are piece‐wise smooth and result from a high‐order nonlinear spatial reconstruction; here we use the WENO method, as presented in Reference 101. The DET method makes succesive use of a solver for the conventional piece‐wise constant data Riemann problem. As already pointed out, the governing Equations (17) are written in non‐conservative form, for which we deploy the path‐conservative framework^102^ to compute the numerical fluctuations, the analogues of the numerical fluxes for conservative methods. As is well known, the presence of source terms in the equations requires a suitable modification of the scheme so as to make it well‐balanced. The present numerical scheme is indeed well‐balanced, as proposed in Reference 60; this is a modification of the original Dumbser–Osher–Toro (DOT)^103^, ^104^ for constructing well‐balanced fluctuations for a first‐order nonoscillatory scheme in the framework of path‐conservative schemes. Full details of the schemes for the present application are found in References 105, 60.

An enhancement to the numerical methodology for the present model of the human circulatory system is the LTS technique,^72^ adapted from Reference 106. The technique is akin to the adaptive mesh refinement methodology,^107^ in which different patches of the full domain are identified by a common spatial discretization length; then each patch is updated with a constant time step for that patch. Explicit methods, such as the present ADER method, are constrained to satisfy the Courant–Friedrichs–Lewy (CFL) stability condition. This is normally implemented on the basis of the maximal wave speed present in the full domain and a minimal length emerging from the spatial discretization. Then, the time step for the updating of each cell is constant, though the local Courant numbers are variable. By allowing a variable time step for each cell, or patches of cells, according to its length and maximal wave speed one may reduce local numerical diffusion and increase efficiency. It is here where the LTS technique can be very useful. The procedure requires a careful global time matching at selected time levels. In our previous works on the human circulatory system^59^, ^70^ we have employed the common technique of a fixed time step per time level. In the present paper, we adopt the LTS technique,^72^, ^106^ in a vessel‐wise manner. It seems as if the first example of a LTS solver applied to blood flow is in Reference 108. In the present paper, we follow,^72^ allowing an explicit local time‐stepping temporal discretization of the underlying finite‐volume type ADER scheme. The net benefits of the LTS technique are two fold: reduced numerical dissipation and enhanced computational efficiency by orders of magnitude.

Considering the introduction of viscoelasticity and the LTS technique, it is essential to adopt a numerical methodology that considers the viscoelastic effects and solves the GRP at the coupling of several 1D vessels (junctions) accurately enough in order to preserve the formal accuracy of the scheme used for the resolution of conservation laws within the 1D domain. For the numerical treatment of the junctions, we follow.^59^, ^72^, ^109^, ^110^ Briefly, the adopted coupling strategy enforces mass conservation and total pressure continuity among vessels sharing a node, while generalized Riemann invariants are used to ensure that coupling conditions and states within 1D domains belong to a smooth solution of the original PDE system.

A succinct description of the numerical methodology for solving the system of PDEs (17) for the human circulatory system has been presented. Next, we describe the solution methods for the equations for the CSF system in the craniospinal space, along with the coupling procedure between the circulation and the CSF and brain dynamics.

### 3.2 Numerical treatment of CSF equations and its coupling to the circulation

**FIGURE 4:**
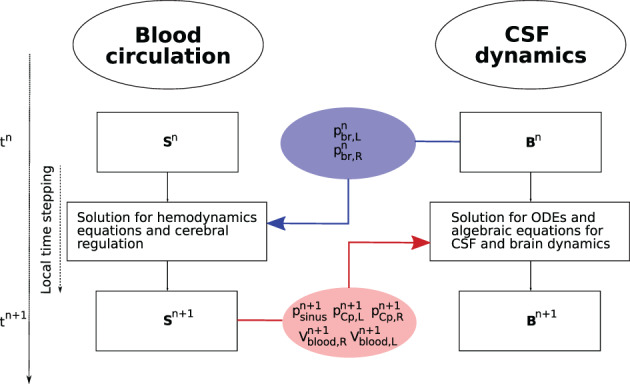
Schematic representation of the coupling between blood circulation and CSF and brain dynamics models. $S^{n}$ and $B^{n}$ are the vectors of unknowns in the blood circulation models (1D vessels and other 0D blood compartments) and in the CSF and brain dynamics model at time $t^{n}$. From $B^{n}$, the pressures of the left and right fluid part of the brain parenchyma $p_{\mathrm{br},R}^{n}$ and $p_{\mathrm{br},L}^{n}$ at time $t^{n}$ are used to find the solution $S^{n+1}$ for the hemodynamics equations and the cerebral regulation. From $S^{n+1}$, the superior sagittal sinus pressure $p_{\text{sinus}}^{n+1}$, the pressure of capillaries $p_{\mathrm{br},R}^{n+1}$, $p_{\mathrm{br},L}^{n+1}$ and the total cerebral blood volume $V_{\text{blood},R}^{n+1}$, $V_{\text{blood},L}^{n+1}$ are used to find the solution $B^{n+1}$ for the CSF and brain equations

The description of CSF and brain dynamics leads to a system of 36 equations with 36 unknowns. The unknowns for each CSF compartment are pressure, cross‐sectional area (which defines a volume since each compartment has an assigned length), inflow and efflux. The coupling between blood flow and CSF dynamics is explicit. The two systems are solved in a sequential manner. As we are using a LTS technique in a vessel‐wise fashion, each 1D vessel is allowed to evolve in time according to a local time step given by its local stability criterion. All vessels have a common synchronization time defined by the prescribed maximum time step $\Delta t_{\max}$ allowed by the LTS procedure. Therefore, the coupling between blood flow and the cranio‐spinal systems is performed every synchronization time $t^{n}=t_{0}+n\;\Delta t_{\max}$, with $t_{0}$ the initial time. Figure 4 describes the coupling procedure from time $t^{n}$ to time $t^{n+1}$. At the beginning of each time step, the vectors $S^{n}$ and $B^{n}$ are known. The vector $S^{n}$ represents the unknowns for the blood circulation system that includes area, flow and pressure in 1D vessels, as well as other 0D blood compartments. The vector $B^{n}$ represents the unknowns for the CSF and brain dynamics models. In the first step, the equations for the blood circulation models are solved. First, the system of ODEs for the cerebral autoregulation model are solved by the explicit Euler scheme, in order to find the new cerebral resistances and compliances, as described in section 2.2.5. Then we solve the system of partial differential equations for blood flow in 1D vessels and the 0D blood compartments for the heart and pulmonary circulation, microvasculature, SR and venous valves. Each *j*th vessel is evolved using the ADER scheme according to its local time step until it reaches the next time step $t^{n+1}=t^{n}+\Delta t_{\max}$. The 0D blood compartments are solved by an explicit Euler scheme and coupled to the 1D vessels. The external pressure for the intracranial 1D vessels and vascular beds, as well as for the SR models, is given by the mean pressure between the left and right sides of the brain parenchyma ($p_{\mathrm{br},R}$ and $p_{\mathrm{br},L}$) at time $t^{n}$. Once the blood circulation equations have been solved, the cerebral capillary pressures ($p_{\mathrm{Cp},R}$ and $p_{\mathrm{Cp},L})$, the superior sagittal sinus pressure ($p_{\text{sinus}}$) and the intracranial blood volume ($V_{\text{blood},R}$, $V_{\text{blood},L}$) are provided to the CSF models. This determines the CSF production, reabsorption rates and the blood volume inside the skull for the Monro–Kellie hypothesis. At this point, the system of differential and algebraic equations for the CSF and brain dynamics are solved by an implicit Euler scheme.

At the beginning of the simulation, given the initial conditions for the blood circulation models, the ODEs and systems of equations for the CSF and brain dynamics models are solved. In this way, the initial intracranial pressures are found and used as external pressure in the first time step update of the blood circulation.

## 4 PARAMETRIZATION OF THE MODEL

In this section, we present all the parameters needed for the implementation of the global closed‐loop model. The parameters assignment correspond to a healthy young male subject. We underline that the parameters are, unless otherwise specified, the ones proposed in References 59, 69.

### 4.1 Blood flow model parameters

#### 4.1.1 Arteries and veins

**FIGURE 5:**
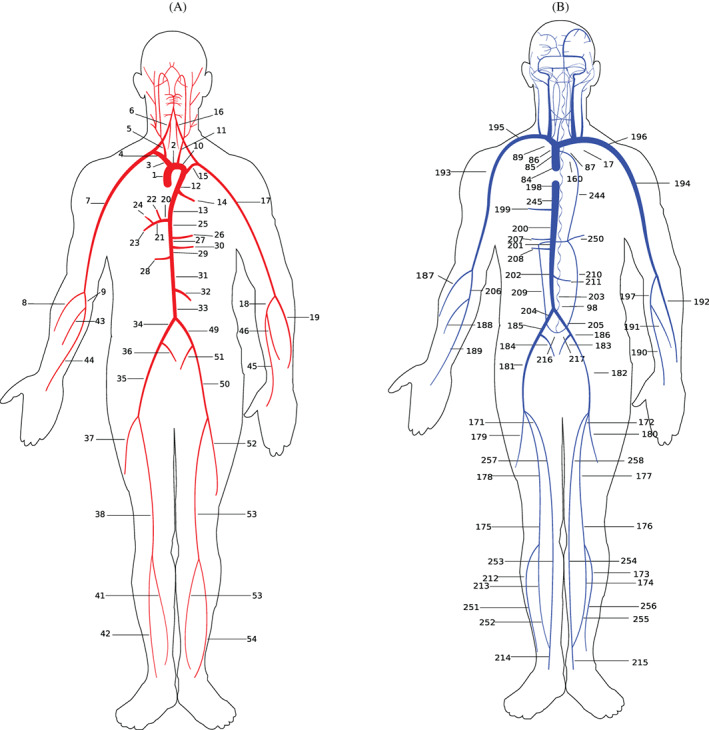
Arterial and venous network composed of 114 arteries and 209 veins; numbers refer to those used in Tables A1 and A2

**FIGURE 6:**
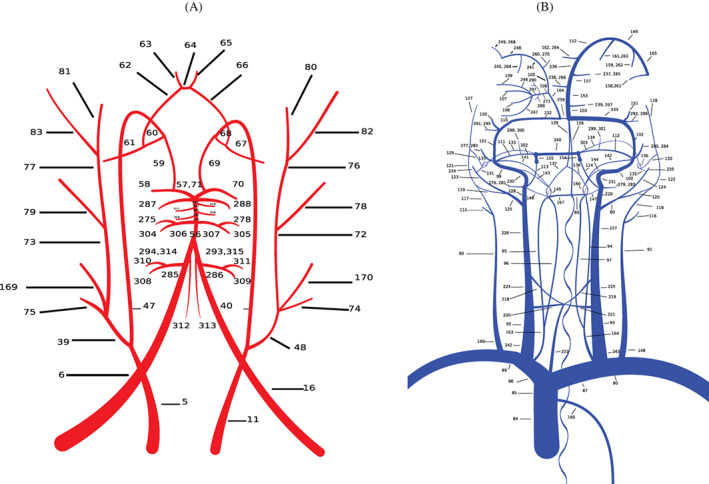
Detail of head and neck arteries (left) and veins (left); numbers refer to those used in Tables A1 and A2

The 1D vascular network contains 323 1D segments, of which 98 are arteries and 209 are veins, all linked by 143 junctions. Figure 5 shows a schematic representation of such networks, while Figure 6a,b provide a detailed description of head and neck arteries and veins. Compared to the network used in References 59, 69, in the present article we propose a detailed description of the highly vascularized regions of the posterior part of the brain. Blood supply to the brainstem is crucial for the function of sensory and motor pathways, as the nerve connections of these systems from the main part of the brain to the rest of the body pass through it. More importantly, the brainstem plays a key role in maintaining cardiac and respiratory functions, such as heart rate and breathing. Three main arteries supply blood to the cerebellum: the superior cerebellar artery (SCA), the anterior inferior cerebellar artery (AICA) and the posterior inferior cerebellar artery (PICA). AICA (No. 304, 305, 306, 307) was previously included in our model for the ear circulation network^68^; the other arteries are added here using data from the ADAN network^111^ and from the literature.^112^ PICA (No. 285, 286, 310, 311) arises from the vertebral artery at about 15 mm from the vertebrobasilar junction. SCA (No. 287, 288) arises from the basilar artery near the bifurcation of the basilar into the posterior cerebral artery. The brainstem is supplied by the medullary branch of the PICA (No. 308, 309), the anterior spinal artery (No. 312, 313), which arises from the terminal part of the vertebral artery and the pontine arteries (No. 316, 317, 318, 319), lateral branches from the basilar artery that supply the pons. All these arteries end in the vascular beds of the cerebellum and brainstem.

According to experimental observations,^113^, ^114^, ^115^ the mean value of blood flow to the cerebellum and the brainstem is about 10% of the total CBF. Following Reference 113, we estimate that flow to the cerebellum and to the brainstem are 1.01 and 0.13 ml/s, respectively. The posterior part of the brain is drained by the group of cerebellar veins, such as the superior cerebellar veins and the inferior cerebellar veins. In this work, the intricate venous vasculature is represented by three main veins: the superior vermian vein (No. 289, 290), the superior petrosal vein (No. 298, 299, 300, 301) in the superior part and the inferior vermian vein (No. 291, 292, 295, 296) in inferior area. The superior vermian vein drains into the vein of Galen (No. 106), the superior petrosal vein drains into the petrous sinus (No. 111, 112) while the inferior vermian vein drains into the transverse sinus (No. 101, 102). The addition of the posterior brain vasculature is essential to explore some medical conditions that affect the brainstem and the cerebellum, such as the effect of vertebral artery hypoplasia in the ipsilateral PICA.^116^ Moreover, in order to analyze better the implications of venous strictures in the pathophysiology of Ménière's disease, we redefine the ear vasculature previously included in References 68. The ear is mainly supplied by the labyrinthine artery,^117^, ^118^, ^119^ which arises from the AICA, passes through the internal acustic meatus and then perfuses the inner ear. More details about the complete vessel network can be found in the Appendix.

The coefficient K present in tube law (5) is obtained from the reference wave speed $c_{0}$ assumed for each vessel; in this work, we estimate its value, distinguishing arteries, veins and dural sinuses. For arteries, this wave speed is computed as proposed by Olufsen et al,^120^ namely,

(84) $$c_{0}^{2}=\frac{2}{3\rho }\left(k_{1}\exp\left(k_{2}r_{0}\right)+k_{3}\right)\;,$$

where $r_{0}$ is the artery's radius at the reference configuration, $k_{1}$, $k_{2}$ and $k_{3}$ are empirical constants and are taken to achieve normal wave speeds in the large vessels for a young adult human and a reasonable increase in smaller vessels. We set $k_{1}=3\times 10^{6}\;g/s^{2}/\mathrm{cm}$, $k_{2}=-7\;{\mathrm{cm}}^{‐1}$ and $k_{3}=40\times 10^{4}\;g/s^{2}/\mathrm{cm}$. Following Reference 59, the venous reference wave speed is estimated as follows

(85) $$c_{0}=c_{0,\max}-\left(c_{0,\max}-c_{0,\min}\right){\left(\frac{r-r_{\min}}{r_{\max}-r_{\min}}\right)}^{\frac{1}{2}}\;,$$

where $c_{0,\max}=400\;\mathrm{cm}/s$, $c_{0,\min}=150\;\mathrm{cm}/s$ and $r_{\max}=0.8\;\mathrm{cm}r_{\min}=0.08\;\mathrm{cm}$ are the maximum and minimum vein radii in the network. Due to the physiological rigid nature of the dural sinuses, for them we set a constant reference wave speed equal to 1500cm/s.

#### 4.1.2 Vascular beds

**FIGURE 7:**
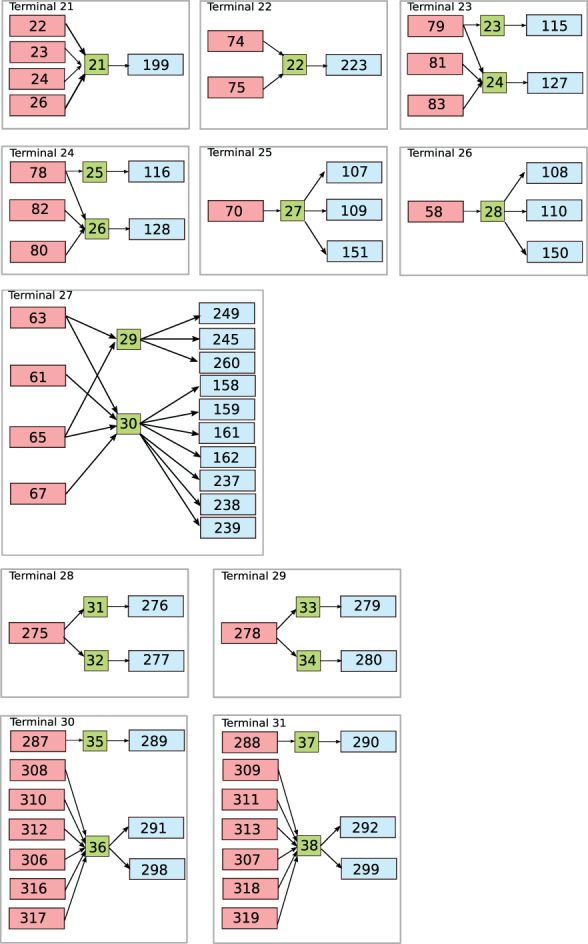
Complex vascular beds: red rectangles refer to connecting arteries, green squares to venous capacitors while blue rectangles to terminal veins, as depicted in Figure 2. Arrows show if an artery is linked to one or both capacitors and the veins connected to each capacitor. Vessel numbers refer to those used in Tables A1 and A2

As a consequence of the addition of vessels to the original network presented in References 59, 69, the present work includes four new terminal models representing the microvasculature of inner ear and brainstem‐cerebellum. Table 1 summarizes the simple connections between one artery and one vein while Figure 7 shows the complex vascular beds.

**TABLE 1:** Vascular beds—simple connections between one artery and one vein. $C_{\mathrm{vn}}$ index: number of the venous capacitor

Terminal index	Artery index	$C_{\mathrm{vn}}$ index	Vein index
1	8	1	187
2	43	2	188
3	44	3	189
4	45	4	190
5	46	5	191
6	19	6	192
7	41	7	251
8	42	8	214
9	37	9	179
10	55	10	215
11	54	11	256
12	52	12	180
13	36	13	184
14	51	14	183
15	32	15	211
16	28	16	208
17	30	17	207
18	169	18	234
19	170	19	235
20	14	20	250

Parameters corresponding to the microcirculation are not always retrievable from the literature; therefore, we use the strategy proposed by References 58 and 61. Total arterial resistance, arterial compliance and venous compliance are fixed up to a constant, according to literature data. Total arterial resistance is fixed to 0.85mmHg/ml while arterial and venous compliances are 1.7ml/mmHg and 146ml/mmHg, respectively. For each terminal artery, we set a total arterial resistance *R* ^*T*^ (taken as the equivalent resistance of the circuit formed by distal arteries, arterioles and capillaries, using data from References 59, 69, and then modified to match the fixed arterial resistance). Then, each resistance is distributed between vascular subsystems according to the general pressure distributions among vascular segments. Arterialcharacteristic impedance $R_{\mathrm{da}}$ is set to be 15% of *R* ^*T*^ while the remaining part is partitioned between arterioles and capillaries as 70% for $R_{\mathrm{al}}$ and 30% for $R_{\mathrm{cp}}$; if an artery splits into two venous capacitors (as for the second artery in the Figure 2), we divide the resistance of the capillaries part according to the flow distribution into venous capacitors. In order to approximate the flow distribution of each venous capacitor, we use the Murray's law, that is, we assume that the flow rate of a vessel is proportional to the cube of its radius; therefore, the flow rate of each venous capacitor is proportional to the sum of the cube of the radii of 1D terminal veins draining from this capacitor. As for the total arterial resistance, the total arterial compliance is fixed to a value taken from Reference 61 and then it is distributed among the respective vascular bed compartments ($C_{\mathrm{art}}$) according to Reference 58. Finally, $C_{\mathrm{al}}$ is set equal to $C_{\mathrm{art}}$ and $C_{\mathrm{cp}}$ is set to be $0.15C_{\mathrm{art}}$. In Table A1 we report the total arterial resistance $R^{T}$ and compliance $C_{\mathrm{art}}$ for each terminal artery.

Concerning the venous compliances, we redistribute the venous compliance of each territory following Liang et al.^58^ If a vascular bed is composed of two venous capacitors, we divide the venous compliance of the entire vascular beds according to flow divisions among them. The venous impedance $R_{\mathrm{vn}}$ is taken as in References 59, 69. Table A2 shows the venous resistance $R_{\mathrm{vn}}$ of each terminal vein and the value of the venous compliance $C_{\mathrm{vn}}$ of the venous capacitor associated to the vein.

#### 4.1.3 Heart and pulmonary circulation

Parameters for heart chambers and cardiac valves are taken from literature References 59 and 61, and then adjusted accordingly to our vessel network. The duration of a cardiac cycle is set to 0.8s. Other parameters are reported in Tables 2 and 3. Parameters for the pulmonary circulation are the same as in the Müller–Toro model, previously taken from References 73, and reported in Table 4. Finally, concerning the pericardium parameters, we set $V_{\mathrm{PC}}$ equal to 400ml and $\Phi_{\mathrm{PC}}$ equal to 100ml.

**TABLE 2:** Heart chambers parameters RA: right atrium, RV: right ventricle, LA: left atrium, LV: left ventricle

	RA	RV	LA	LV
$E_{A}\left(\text{mmHg}/\mathrm{ml}\right)$	0.07	0.55	0.07	2.75
$E_{B}\left(\text{mmHg}/\mathrm{ml}\right)$	0.04	0.05	0.09	0.12
$T_{\mathrm{cp}}\left(s\right)$	0.25	0.4	0.17	0.4
$T_{\mathrm{rp}}\left(s\right)$	0.17	0.15	0.17	0.15
$t_{c}\left(s\right)$	0.7	0.3	0.8	0
$t_{r}\left(s\right)$	0.97	0.0005	0.97	0.3
α	0.0005	0.0005	0.0005	0.001
$P_{\mathrm{ini}}\left(\text{mmHg}\right)$	5.09	5.06	6.56	8.6

**TABLE 3:** Cardiac valves parameters. TriVal: tricuspid valve, PulVal: pulmonary valve, MitVal: mitral valve, AorVal: aortic valve

	TriVal	PulVal	MitVal	AorVal
$M_{s}$	1	1	1	1
$M_{r}$	0.00001	0.00001	0.00001	0.00001
$K_{o}$ (cm^2^/dynes/s)	0.03	0.02	0.02	0.02
$K_{c}$ (cm^2^/dynes/s)	0.04	0.02	0.04	0.02
$l_{e}$ (cm)	2	1.5	2	1
$A_{a}$ (cm^2^)	6	5.7	5.1	4.9

**TABLE 4:** Parameters for pulmonary circulation. $E_{0}$: baseline elastance (mmHg/ml); Φ: volume constant (ml); R: resistance (mmHg/s/ml); L: inductance (mmHg/s^2^/ml); S: viscoelasticity (mmHg/s/ml)

	$E_{0}$	Φ	R	L	S
Artery	0.02	20.0	0.040	0.0005	0.01
Capillary	0.02	60.0	0.040	0.0005	0.01
Vein	0.02	200.0	0.005	0.0005	0.01

#### 4.1.4 Venous valves and Starling resistors

According to the vessel network extension, this work presents additional valves and SR, for a total of 17 valves and 21 resistors. Table 5 shows the location of these elements in the vessel network. Parameter values for venous valves are set to describe a normal functioning valve and are given by $M_{s}=1$, $M_{r}=0.001$, $K_{o}=133.32\;\frac{1}{\text{mmHg}\;s}$, $K_{c}=40\;\frac{1}{\text{mmHg}\;s}$. Parameters for SR are set to $M_{s}=0.5$, $M_{r}=0.05$, $K_{o}=K_{c}=133.32\;\frac{1}{\text{mmHg}\;s}$.

**TABLE 5:** Location of venous valves (on the left) and Starling resistors (on the right). Vessels numbers refer to those used in Table A2

No.	Left vessel index	Right vessel index
1	193	195
2	194	196
3	244	160
4	257	171
5	258	172
6	253	257
7	254	258
8	175	178
9	176	177
10	251	212
11	256	173
12	252	213
13	255	174
14	92	242
15	93	243
16	90	140
17	91	148

No.	Left vessel index	Right vessel index
1	158	261
2	159	262
3	161	263
4	162	264
5	237	265
6	238	266
7	239	267
8	249	268
9	245	269
10	260	270
11	271	106
12	150	272
13	151	273
14	276	281
15	277	282
16	279	283
17	280	284
18	291	295
19	292	296
20	298	300
21	299	301

#### 4.1.5 Autoregulation

The cerebral autoregulation model works on 12 terminal cerebral arteries; the baseline haemodynamic parameters of these arteries are set from the periodic solution obtained for a baseline simulation and reported in Table 6.

**TABLE 6:** Baseline values of cerebral haemodynamic variables obtained from a periodic baseline simulation

No.	Vessel name	${\bar{Q}}^{T}$ [ml/s]	${\bar{R}}^{T}$ [mmHg/ml]	$\bar{C}$ [mmHg/ml]	${\bar{V}}^{T}$ [ml]
58	Right posterior cerebral artery II	1.42	39.15	3.324E‐06	0.26
61	Right middle cerebral artery	3.01	19.06	6.649E‐06	0.55
63	Right anterior cerebral artery II	1.54	38.08	3.324E‐06	0.28
65	Left anterior cerebral artery II	1.54	38.08	3.324E‐06	0.28
67	Left middle cerebral artery	3.01	19.06	6.649E‐06	0.55
70	Left posterior cerebral artery II	1.42	39.15	3.324E‐06	0.26
287	Right SCA	0.30	132.55	6.649E‐07	0.06
288	Left SCA	0.30	132.55	6.649E‐07	0.06
306	Right AICA II	0.08	714.26	6.589E‐07	0.058
307	Left AICA II	0.08	714.26	6.589E‐07	0.058
308	Right PICA MB	0.005	11885.65	6.589E‐07	0.058
309	Left PICA MB	0.005	11,887.52	6.589E‐07	0.058
310	Right PICA II	0.13	449.13	6.589E‐07	0.058
311	Left PICA II	0.13	449.13	6.589E‐07	0.058
312	Right anterior spinal a.	0.06	1048.93	6.589E‐07	0.058
313	Left anterior spinal a.	0.06	1048.93	6.589E‐07	0.058
316	Right pontine a. I	0.001	63,752.68	6.589E‐07	0.058
317	Right pontine a. II	0.001	63,669.28	6.589E‐07	0.058
318	Left pontine a. I	0.001	63,726.28	6.589E‐07	0.058
319	Left pontine a. II	0.001	63,668.30	6.589E‐07	0.058

Other parameters of the model are taken from Ursino and Giannessi work^75^ and adjusted to match our cerebral vessel network values (Table 7). For each terminal artery, the gain of autoregulation $G_{\mathrm{aut},i}$ is computed from the total G, according to the flow distribution inside the brain.

**TABLE 7:** Parameters for the autoregulation model, taken from Reference 75

Parameter	Value
τ (s)	20
G	0.9
${\mathrm{sat}}_{1}$	0.55
${\mathrm{sat}}_{2}$	2.0

### 4.2 CSF model parametrization

Parameters for the CSF model are based on Linninger et al.^62^ Table 8 shows length and area at rest of the cylindrical volume representing each cerebral compartment, and different values of elastance taken from the literature. Table 9 reports flow resistance values; they account for the pressure drop in the fluid along the length of a compartment due to viscous forces and they are obtained from the dynamic fluid viscosity μ, the length of the compartment l and the square of the compartments' cross‐sectional area.

**TABLE 8:** Hydraulic length, area at rest and elastance of each cerebral compartment

Compartment	Length [cm]	Area at rest [${\mathrm{cm}}^{2}$]	Elastance [mmHg]
LVs	0.75	12	7.55
V	1	2.5	7.55
AoS	1.8	0.00785	7.55
V	1	3.5	7.55
CSAS	1.69	17.76	80
SSAS	43	2	160
Brain parenchyma (Fluid)	14	30	
Brain parenchyma (Solid)	14	70	

**TABLE 9:** Flow resistances of cerebral compartments

Compartment	Resistance [mmHg/ml]
3V—*R*_3*V*_	0.2
AoS—*R*_*AoS*_	5.5
V—*R*_4*V*_	0.2
CSAS—*R*_*SSAS*_	0.2
SSAS—*R*_*CSAS*_	0.1
Brain—*R*_*br*_	81,520
Brain—*R*_*br*,2_	500

As already written in section 2.3, the CSF model adopted here accounts for constant production of CSF, from capillaries $q_{\mathrm{Cp}\to \mathrm{br},\text{const}}$ and from arterioles to lateral ventricles $q_{\mathrm{Al}\to \mathrm{LV},\text{const}}$. Almost two‐thirds of the total CSF production takes place in the choroid plexus of the lateral ventricles; it was found clinically that this process is almost invariant to pressure changes suggesting an active transport process.^121^ As in Reference 62, we fix a constant mass transfer independent from pressure equal to $q_{\mathrm{Al}\to \mathrm{LVs},\text{const}}=0.00583$ ml/s. Moreover, there is CSF mass transfer from capillary beds into the brain parenchyma; the constant diffuse CSF production is set equal to $q_{\mathrm{Cp}\to \mathrm{br},\text{const}}=0.0005$ ml/s. The active exchange between capillaries and brain parenchyma is governed by Equations (62) and (63), where CSF seepage is governed by pressure differences.

CSF reabsorption is described in (80) by a mass transfer flux that is a function of the pressure difference between the subarachnoid space and the superior sagittal sinus and a reabsorption constant k. In this work, we use k=0.0027 mmHg/ml/s. We underline that variation of reabsorption constant could simulate pathological situations; for example, an increase of the reabsorption resistance may be due to inflammation of meninges while acute communicating hydrocephalus could be simulated by reducing k_._ ^48^, ^62^

## 5 SAMPLE NUMERICAL RESULTS AND VALIDATION

In this section, we present computational results obtained with the presented model in order to perform a comprehensive validation of the model's outputs. 1D domains are divided into computational cells with a reference length of Δx=1cm, imposing a minimum of one computational cell in each vessel. Once that the mesh spacing of a vessel is fixed, the respective relaxation time ɛ for each vessel is computed in order to ensure that the accuracy criterion for the hyperbolic reformulation proposed in Reference 70 is satisfied. The CFL coefficient is set to CFL=0.9 according to the linear stability limit of ADER finite volume schemes for 1D problems. A maximum time step of $\Delta t_{\max}=1\times 10^{-3}\;s$ is allowed. All computations are run using a second‐order accurate version of the numerical scheme previously described. Other parameters linked to the blood characteristics are the blood viscosity taken as μ=0.045P and the blood density $\rho =1.06\;g/{\mathrm{cm}}^{3}$. The reference pressures taken as initial conditions are reported in Table 10. Given the closed‐loop nature of our model, such pressures are important since they determine the periodic solution that the system will reach by defining the stretched blood volume. All the computational results shown in this section are obtained with simulations of 2000 cardiac cycles. A periodic state is reached after approximately 1600 cycles; compared to References 59, the time used to reach the periodicity of the simulation is higher due to coupling between two systems (blood and CSF) that have different time scales. Therefore, the verification of convergence of the solution is mainly based on the equality of the CSF production and CSF reabsorption, since CSF production involves the arterial pressure, and the CSF reabsorption rate is related to intracranial venous pressure. While future work will regard a more efficient treatment of coupling for the two systems under investigation, it is interesting to note that this difference in time scales poses severe constrains as to the mass conservation properties of the numerical schemes used to solve this problem. In fact, a discretization that is not able to enforce mass conservation at a discrete level would result in inability to reach a periodic solution due to mass conservation error accumulation.

**TABLE 10:** Initial pressure conditions for vascular compartments

Compartment	$P_{\mathrm{ini}}$ [mmHg]
Arteries	70.0
Veins	5.0
Arterioles	45.0
Capillaries	25.0
Venules	10.0

### 5.1 Validation of systemic haemodynamics

#### 5.1.1 Arteries and veins

**FIGURE 8:**
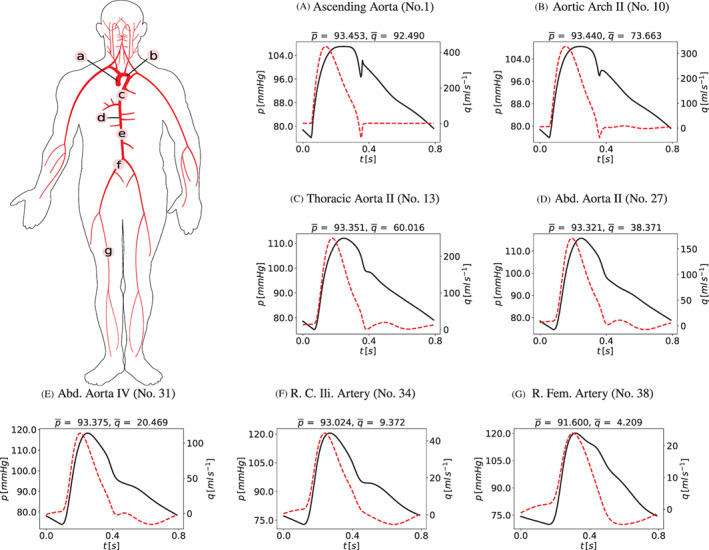
Computed blood pressure p (continuous black line) and blood flow q (dashed red line) in the aortic tree at different locations (a) to (g). Cardiac‐cycle averaged values are denoted by $\bar{p}$ and $\bar{q}$

**FIGURE 9:**
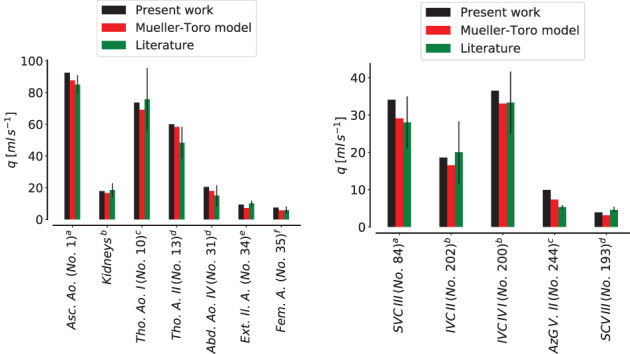
Blood flow distribution along in selected systemic arteries (left frame) and veins (right frame): computational results of the present model, computational results from Reference 59 and literature data (average and standard deviation). Asc. Ao.: Ascending Aorta; Kidneys: sum of both Renal Arteries; Tho. Ao.: Thoracic Aorta; Abd. Ao.: Abdominal Aorta; Ext. Il. A.: External Iliac Artery; Fem. A.: Femoral Artery; SVC: Superior Vena Cava; IVC: Inferior Vena Cava; AzG V.: Azygos Vein; SCV: Subclavian Vein. Literature: ^*a*^Murgo et al^122^; ^*b*^Wolf et al^123^; ^*c*^Zitnik et al^124^; ^*d*^Cheng et al^125^; ^*e*^Itzchak et al^126^; ^*f*^Lewis et al^127^; ^*g*^ Be′eri et al^128^; ^*h*^ Cheng et al^125^; ^*i*^ Nabeshima et al^129^; ^*j*^ Fortune & Feustel.^130^

**FIGURE 10:**
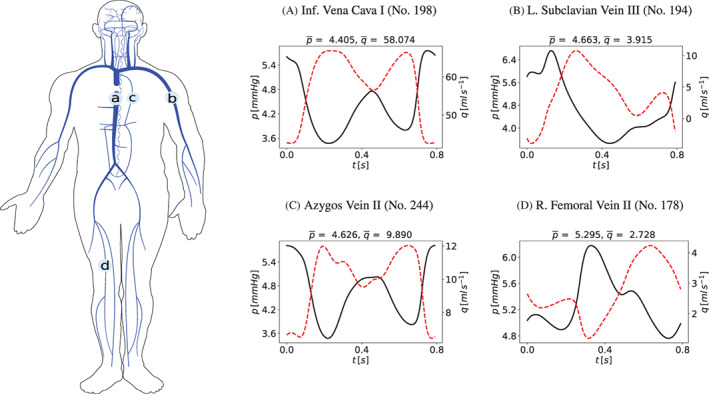
Computed blood pressure p (continuous black line) and blood flow q (dashed red line) in selected systemic veins in different locations (a) to (d). Cardiac‐cycle averaged values are denoted by $\bar{p}$ and $\bar{q}$

Figures 8, 9, 10 show computed pressure and flow rate at the midpoint of selected vessels in the arterial and venous networks. In particular, Figure 8 shows the computed waveforms along the aorta and major arteries of the lower limb. We can notice that, as the wave travels away from the heart toward the periphery, the systolic peak pressure increases according to physiological patterns, with a pulse pressure from 25 mmHg in ascending aorta to 45 mmHg in femoral artery. Moreover, the pressure range covers normal values of a young subject. Along the aorta pathway, the peak flow decreases progressively. Flow distribution along systemic arteries is assessed by comparing computational results with literature data and results obtained with the previous version of the model^59^; the corresponding bar plots are reported in Figure 9 (left). We note that when using the terminology *literature data* we always make reference to experimental data gathered in vivo and published by other research groups. Main cardiovascular indexes, such as systolic, diastolic and mean blood pressure and pulse pressure are computed and compared to literature data in Table 11. We conclude that waveform patterns in the arterial system are in accordance with general physiological data and that blood flow distribution along the aorta is reasonable. Concerning the venous circulation, Figure 10 shows the pressure and flow rate along the main systemic veins while Figure 9 (right) depicts a comparison of the predicted flow at different locations of the systemic venous circulation with literature data and results obtained in Reference 59.

**TABLE 11:** Cardiovascular indexes

Index	Current value	Ref. value	References
SBP [mmHg]	107.065	105 ± 8	^131^
DBP [mmHg]	76.126	71 ± 7	^131^
MBP [mmHg]	93.272	89 ± 8	^131^
PP_*Aorta*_ [mmHg]	30.939	30 ± 6	^131^
PP_*Brachial*_ [mmHg]	37.382	49 ± 9	^131^
PP_*Amplification*_ [mmHg]	1.208	1.7 ± 0.14	^131^
CO [ml/s]	91.363		
C_*a*_ [ml/mmHg]	2.001	1.7	^132^
E_*es*_ [mmHg/ml]	5.205	4.5	^133^
E_*a*_ [mmHg/ml]	2.746	2.3	^133^
E_*a*_/E_*es*_	0.528	0.58	^133^
LV_*max*_	114.263	150 ± 67	^61^
LV_*EF*_	0.655	0.68 ± 0.12	^61^
max. $\frac{{\mathrm{dP}}_{\mathrm{LV}}}{\mathrm{dt}}$	1546.419	1915 ± 410	^61^
min. $\frac{{\mathrm{dP}}_{\mathrm{LV}}}{\mathrm{dt}}$	−2861.828	−2296 ± 530	^61^

*Note*: Num. Value: computed numerical value; Ref. value: literature reference value with mean and standard deviation. (S/D) BP: systolic/diastolic aortic blood pressure; MBP: mean blood pressure; PP: pulse pressure in aortic root and in brachial artery; PP_*Amplification*_: ratio between pulse pressure in brachial artery and aortic root; CO: cardiac output; C_*a*_: arterial compliance evaluated as the ratio between stroke volume and brachial pulse pressure^132^; E_*a*_: arterial elastance; E_*es*_: left ventricle elastance; E_*a*_/E_*es*_: arterial‐ventricular coupling index; LV_*max*_: maximum left ventricle volume; LV_*EF*_: averaged left ventricle volume; max. $\frac{{\mathrm{dP}}_{\mathrm{LV}}}{\mathrm{dt}}$: maximum pressure rate of left ventricle; min. $\frac{{\mathrm{dP}}_{\mathrm{LV}}}{\mathrm{dt}}$: minimum pressure rate of left ventricle.

**FIGURE 11:**
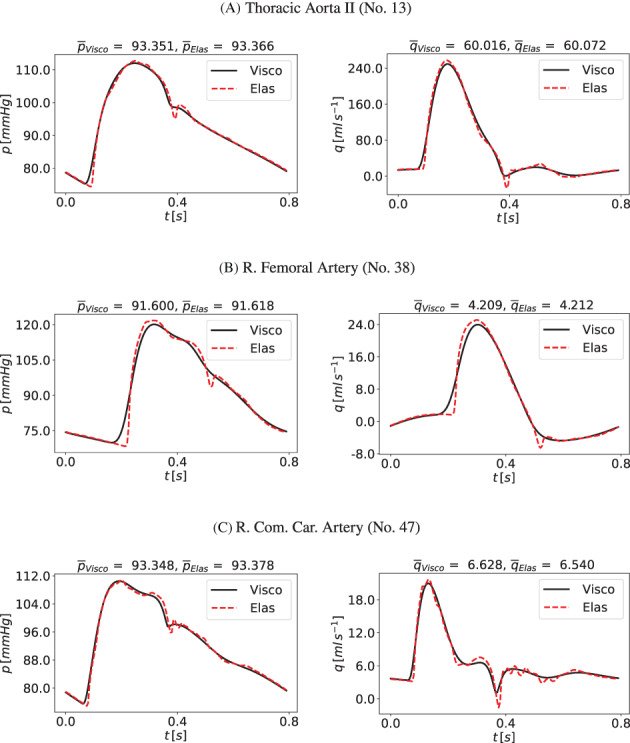
Computed blood pressure p and blood flow q in selected arteries obtained with viscoelastic (continuous black line, Visco) and elastic (dashed red line, Elas) model for vessels wall. Cardiac‐cycle averaged values are denoted by ${\bar{p}}_{\text{Visco}}$ and ${\bar{q}}_{\text{Visco}}$ for viscoelastic vessels and by ${\bar{p}}_{\text{Elas}}$ and ${\bar{q}}_{\text{Elas}}$ for elastic vessels

**FIGURE 12:**
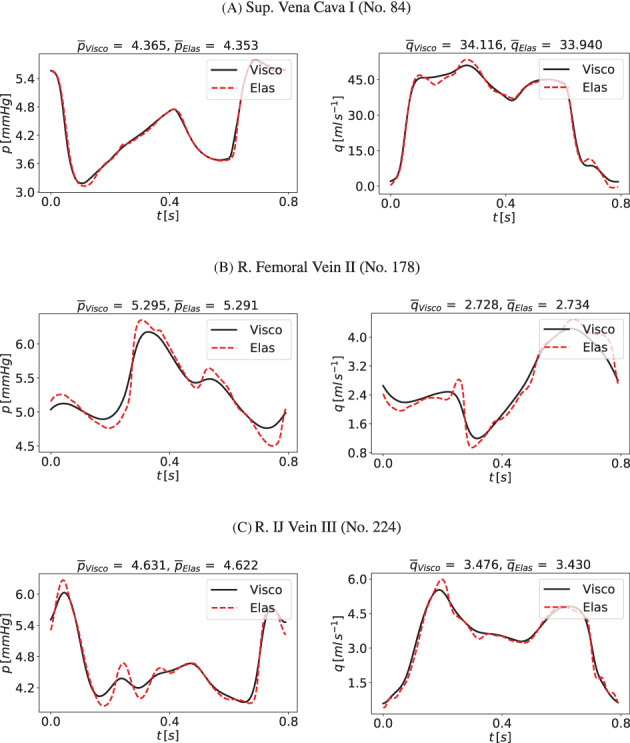
Computed blood pressure p and blood flow q in selected veins obtained with viscoelastic (continuous black line, Visco) and elastic (dashed red line, Elas) model for vessels wall. Cardiac‐cycle averaged values are denoted by ${\bar{p}}_{\text{Visco}}$ and ${\bar{q}}_{\text{Visco}}$ for viscoelastic vessels and by ${\bar{p}}_{\text{Elas}}$ and ${\bar{q}}_{\text{Elas}}$ for elastic vessels

It is well known that blood flow in large to medium vessels is a convection‐dominated process; therefore neglecting viscoelasticity of vessel walls in 1D models is often chosen as compromise. However, the viscoelastic behavior of arterial and venous walls is well‐known. It has an impact on fundamental hemodynamic characteristics of the cardiovascular system and plays a determinant role in setting the functional level of the cardiovascular system under physiological and under pathological conditions. Previous works^39^, ^134^, ^135^ have shown the benefits of considering viscoelastic properties of vessel walls in arterial circulation comparing model predictions and in vivo measurements of pressure and flow at different location. The effects of viscoelasticity become more significant in the periphery, especially on the flow wave. The same happens in the venous circulation. Zocalo et al^136^ showed the importance of the dynamic process of veins walls to understand venous functioning under normal and pathological conditions; pressures and diameters of anterior cava, jugular and femoral veins from sheep were registered during cyclical volume‐pressure pulses. The vein viscosity was higher in the peripheral segments and this could be important in the response to acute overloads and in haemodynamic control. In this work, we introduce a viscoelastic tube law not only for the arterial tree but also in the venous circulation. Both arteries and veins are represented as a Voigt‐type visco‐elastic material (Equation 7). Figures 11 and 12 compare computed pressure and flow rate when vessels are represented with elastic and viscoelastic behavior of their walls. Figure 11 refers to thoracic aorta, femoral and carotid artery while Figure 12 depicts superior vena cava, femoral and jugular vein; the effect of the viscoelastic tube law is more evident in peripheral vessels (femoral artery and vein, carotid artery and jugular vein) with respect to central vessels. In particular, it can be seen that the solution obtained for viscoleastic vessels presents significantly less high‐frequency components with respect to the solution obtained for elastic vessels. This fact is consistent with the dissipative capacity of real vessels, which, at least for physiological states, do not display pressure and flow waveforms with very high‐frequency components.

#### 5.1.2 Vascular beds

**FIGURE 13:**
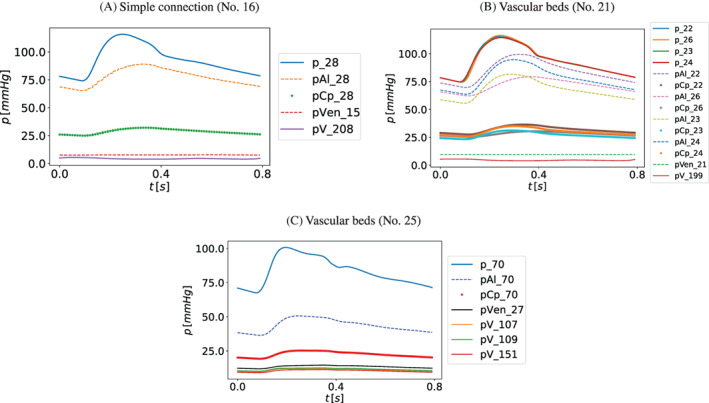
Computed pressure values for three vascular beds. p: pressure of supplying artery; pAl: pressure in arterioles; pCp: pressure in capillaries; pVen: pressure in venules at venous capacitor; pV: pressure of draining vein. Numbers for supplying arteries and draining veins refer to numeration in Tables 1 and 7

Figure 13 shows the computed pressures during a cardiac cycle for three different compartments. The pressure values display a physiologically behavior in all compartments; from arterioles to venules, the pressure slowly decreases. It ranges between 40 and 80 mmHg for arterioles, between 20 and 25 mmHg for capillaries and between 5 and 15 mmHg for venules. In particular, 13a refers to a simple connection in the kidney, 13b is a vascular bed in the abdominal region with four supplying arteries and one draining vein while 13c represents the microcirculation pressures in the left part of the posterior brain.

#### 5.1.3 Heart

**FIGURE 14:**
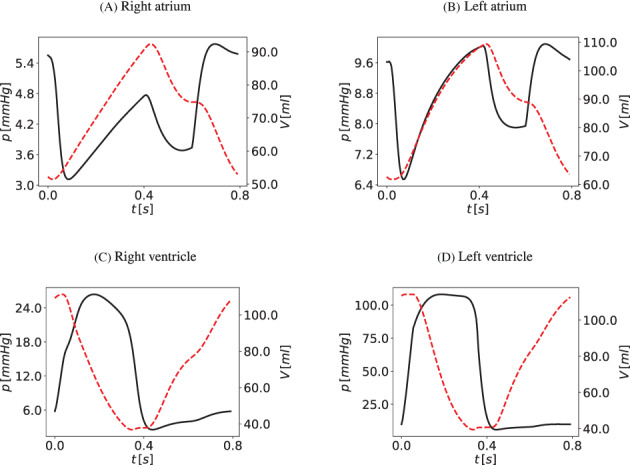
Computed pressures p and volumes V in the heart. Continuous black line denotes pressure, dashed red line refers to volume

**FIGURE 15:**
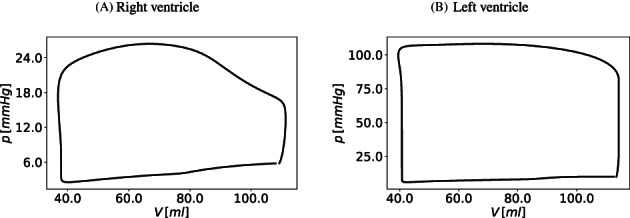
Computed Pressure‐Volume loop for the right and left ventricles

Figure 14 shows the computed pressures and volumes for the four cardiac chamberswhile Figure 15 displays the pressure‐volume relationship for the left and right ventricles. The heart model well represents the physiological variations of pressure over the cardiac cycle for both atria and ventricles. Moreover, Table 11 compares the predicted values for selected cardiovascular indexes to literature data, showing overall satisfactory agreement.

### 5.2 Validation of cerebral haemodynamics

**FIGURE 16:**
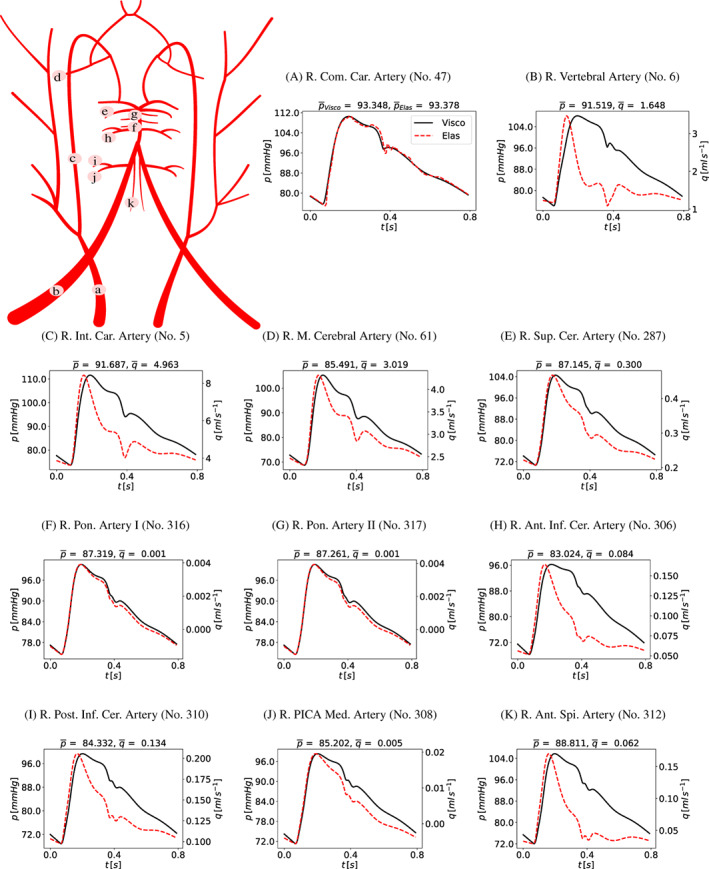
Computed blood pressure p (continuous black line) and blood flow q (dashed red line) in the main cerebral and neck arteries. Cardiac‐cycle averaged values are denoted by $\bar{p}$ and $\bar{q}$

**FIGURE 17:**
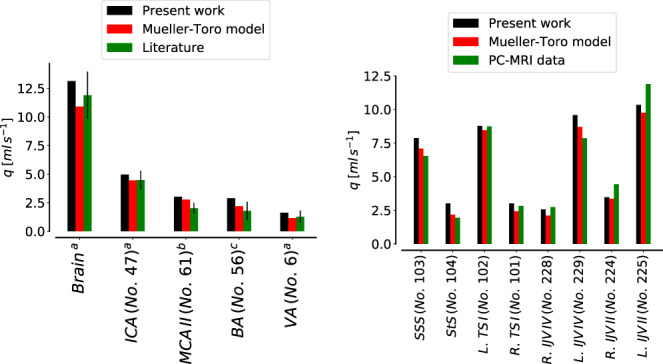
Comparison between present computed results, other computed results^59^ and literature data (average and standard deviation) or MRI flow quantification data^59^ for blood flow in head and neck arteries and veins. Brain: sum of average flow rate in both internal carotid and vertebral arteries; ICA: Internal Carotid Artery; MCA: Middle Cerebral Artery; BA: Basilar Artery; VA: Vertebral Artery; SSS: Superior sagittal Sinus; StS: Straight Sinus; TS: Transverse Sinus; IJV: Internal Jugular Vein. Literature: ^*a*^Stoquart‐ElSankari et al^137^; ^*b*^Stock et al^138^; ^*c*^Boorder et al^139^

**FIGURE 18:**
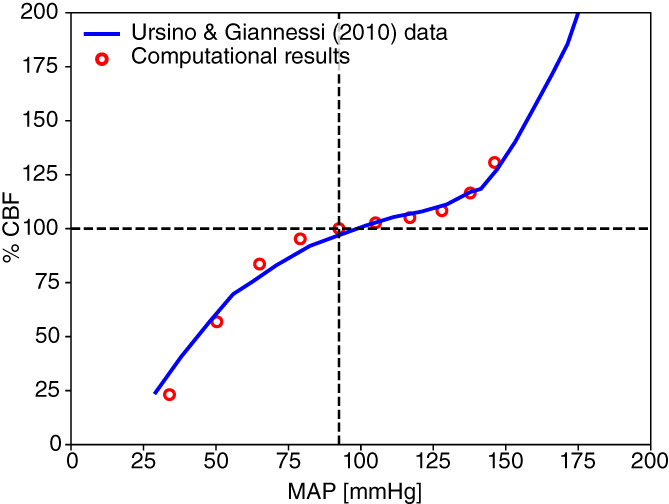
Computed autoregulation curve (blue continuous line) compared to Ursino's data^78^ (red dots). Mean arterial pressure, MAP, against the percentage change in cerebral blood flow, % CBF, with respect to the baseline situation

Figure 16 illustrates predicted pressure and flow waveforms in the head and neck arterial circulation. Moreover, flow distribution among cerebral arteries is assessed via comparison to literature data and results reported in Reference 59 in Figure 17 (left). The functioning of cerebral autoregulation is verified by changing arterial resistances of all but the cerebral arteries in order to cause a mean arterial pressure change, which would cause an increment in cerebral flow if peripheral cerebral resistance would not adapt. Figure 18 shows the computed autoregulation curve compared with literature data from Reference 75. The autoregulation curve relates mean arterial pressure (pressure of vessel No. 1) and the percentage change in CBF with respect to the baseline situation (evaluated as sum of mean flow over a cardiac cycle of internal carotid arteries and vertebral arteries).

**FIGURE 19:**
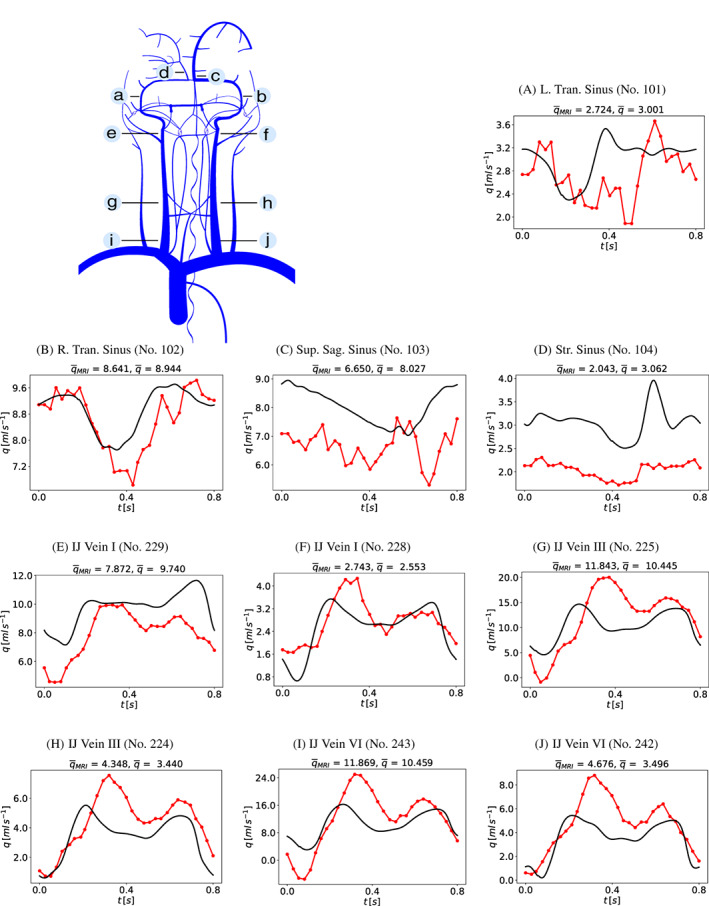
Comparison between computed blood flow q and PC‐MRI flow quantification^59^ in dural sinuses and internal jugular veins. Full line denotes present model results, PC‐MRI flow quantification data is shown with symbols and full line. Cardiac‐cycle averaged values are denoted by $\bar{q}$ and ${\bar{q}}_{\mathrm{MRI}}$

Particular attention is given to the head and neck veins; in this case, PC‐MRI flow measurements were available from Reference 59; these data were collected by the MR Research Facility at Wayne State University, Detroit (USA) and were used in Reference 59 to construct the head and neck venous network of the present model. For details on the image acquisition procedure and a discussion on expected agreement between MRI‐derived flow and model predictions refer to Reference 59; flow waveforms are compared with the underlying patient‐specific MRI flow quantification data. Furthermore, the average flow rate is compared with phase‐contrast MRI data in Figure 17 (right). Predicted flow waveforms display characteristic features of cerebral venous flow with a bi‐phasic character. Moreover, agreement of PC‐MRI‐derived average flows and predicted ones is reasonable (Figure 19).

**FIGURE 20:**
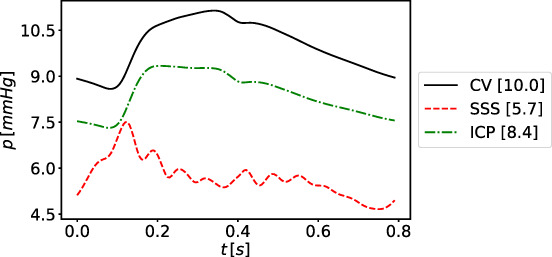
Computed cerebral venous pressure p and cerebrospinal fluid dynamics. CV: cerebral vein (No. 158); SSS: superior sagittal sinus (No. 165); ICP: intracranial pressure (pressure of the fluid parts of the brain parenchyma compartments)

**FIGURE 21:**
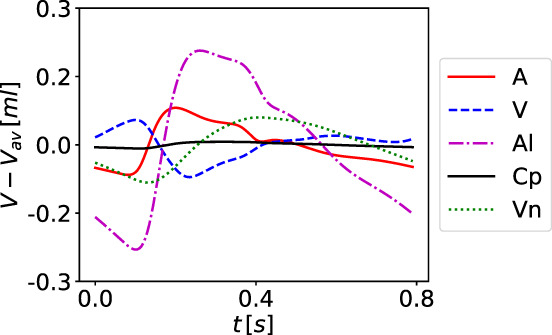
Variation in time of $V-V_{\mathrm{av}}$ where V is the volume of a compartment and $V_{\mathrm{av}}$ is the averaged volume over a cardiac cycle. A: arteries; Al: arterioles; Cp: capillaries; Vn: venules; V: veins

Figure 20 depicts computed intracranial pressure and pressures of a dural sinus and a cerebral vein; the effect of the SR is evident: pressure in cerebral veins is always higher than intracranial pressure while dural sinus pressure is governed by downstream conditions. Figure 21 shows the changes in volume of the main blood cerebral compartments: arteries, arterioles, capillaries, venules and veins.

### 5.3 Validation for CSF and brain dynamics

**FIGURE 22:**
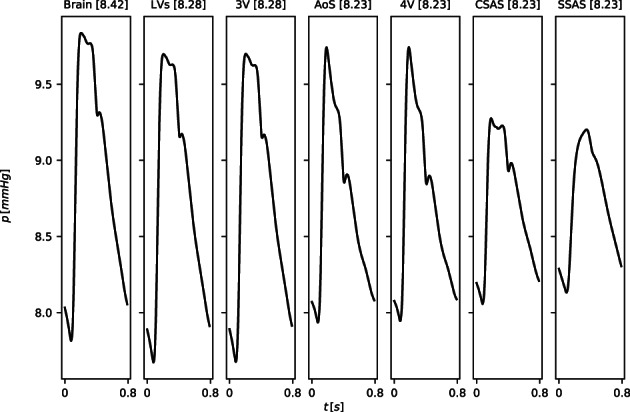
Pressure in cerebrospinal fluid compartments over a cardiac cycle. Mean pressure over the cardiac cycle is reported in brackets. Brain: pressure in fluid part of brain parenchyma; LVs: pressure in lateral ventricles; 3V: third ventricle; AoS: aqueduct of Sylvius; 4V: fourth ventricle; CSAS: cranial subarachnoid space; SSAS: spinal subarachnoid space

**FIGURE 23:**
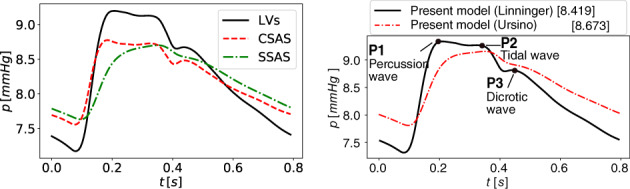
Cerebrospinal fluid pressure. Left frame shows computed pressure of lateral ventricles (LVs), cranial subarachnoid space (CSAS) and spinal canal (SSAS). Right frame shows computed intracranial pressure with present blood circulation model and the Linninger's CSF model^62^ with analysis of the peaks following^140^ and computed intracranial pressure with current blood circulation model and Ursino's CSF model^141^

**FIGURE 24:**
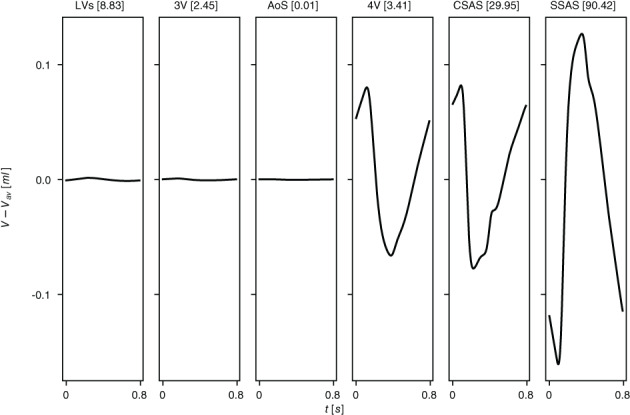
Cerebrospinal fluid volumes. Variation in time of $V-V_{\mathrm{av}}$, where V is the volume of the compartment and $V_{\mathrm{av}}$ is the averaged volume over the cardiac cycle (value reported in brackets). LVs: lateral ventricles; 3V: third ventricle; AoS: aqueduct of Sylvius; 4V: fourth ventricle; CSAS: cranial subarachnoid space; SSAS: spinal subarachnoid space

**FIGURE 25:**
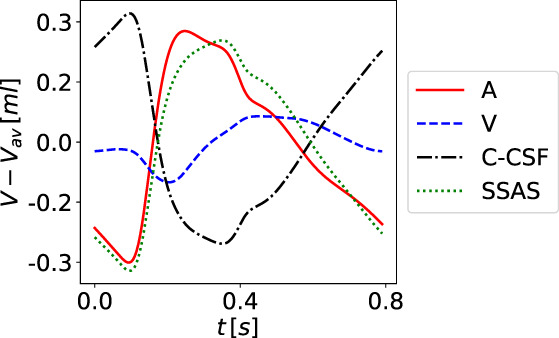
Time variation of $V-V_{\mathrm{av}}$ of fluid volumes within the cardiac cycle, where V is the volume of the compartment and $V_{\mathrm{av}}$ is the averaged volume over the cardiac cycle. A: cerebral 1D arteries, arterioles and capillaries; V: cerebral 1D veins and venules; C‐CSF: cranial CSF (CSF in all compartments inside the skull); SSAS: spinal CSF

Here, we analyze pressure, flow and volume of different CSF compartments and their interaction with arterial and venous blood within the brain; see Figures 22, 23, 24, 25. Physiological intracranial pressure (ICP) values have been investigated extensively, ranging from 7 to 15 mmHg for an adult in supine position.^142^ Generally, ICP refers to the CSF pressure, regardless of where it is measured. In our simulation, slight differences are found between the mean pressure of the extracellular fluid part of the brain parenchyma and other cranial and spinal CSF compartments. In this context, we call ICP the pressure of CSF located in the brain parenchyma compartments; specifically, ICP is defined as $\frac{1}{2}\left(p_{\mathrm{br},R}+p_{\mathrm{br},L}\right)$, that is the average between the CSF pressure of the right and left extracellular fluid part of the brain parenchyma compartments. Figure 22 reports the pressures of the CSF compartments. The ventricular pressure ranges from 7.7 to 9.7 mmHg with a pulse pressure of about 2 mmHg. The cranial and SSASs pressures values lie between 8.1/8.2 and 9.3/9.2 mmHg and the pulse pressure is around 1.0 mmHg. Comparing the left and right hemisphere, there are no distinct differences concerning pressures. The brain parenchymal pressure varies from 7.8 to 9.8 mmHg, with a mean value of 8.42 mmHg on the right and left sides.

Figure 23 shows an analysis of the intracranial pressure waveform according.^143^, ^144^, ^145^, ^146^ We can notice three physiological peaks: the first one, P1 or percussion wave, is the highest, followed by P2 or tidal wave and finally there is P3 or dicrotic wave, which appears after the dicrotic notch. The peaks come from the arterial pulse wave from the heartbeat on the brain which essentially floats in CSF; the ICP waveform can usually be seen in time‐synchronized fashion relation to the arterial waveform. The three typical peaks of the intracranial pressure can be observed in all CSF compartments but in the spinal canal they are less pronounced. P1 is caused by the arrival of the arterial flow pulse. P2 is caused by the second arterial flow peak, which happens before the dicrotic notch, while the third peak, P3, is related to the increased flow occurring right after the dicrotic notch.

Figure 23 compares the intracranial pressure waveform of the current CSF model based on Reference 62 and that evaluated considering the simple CSF model proposed by Reference 141 and adopted in Reference 69. While mean ICP depends on initial conditions for both models, in this second case, the waveform peaks are less well defined. Figure 24 reports changes in volume of the CSF model compartments. The major part of CSF is contained in the cranial and SSASs, about 30 and 90 ml respectively, where the changes over the cardiac cycle tale place.

**FIGURE 26:**
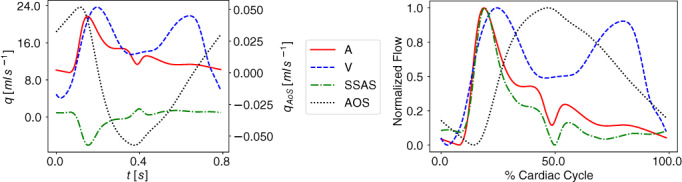
Time variation of blood and cerebrospinal fluid (CSF) flow within a cardiac cycle. Left frame shows blood and CSF flow within a cardiac cycle, where axis on the right refers to AoS flow. Right frame shows CSF and blood normalized flow analysis over a cardiac cycle. To highlight the temporal dynamic sequence in the four fluid compartments, each flow profile was normalized between 0 and 1 such that all four systolic peaks correspond to 1. A: arterial flow of internal carotid arteries and vertebral arteries at C2C3 level; V: internal jugular veins flow at C2C3 level; SSAS: flow of the spinal subarachnoid space; AOS: flow in the aqueduct of Sylvius

**FIGURE 27:**
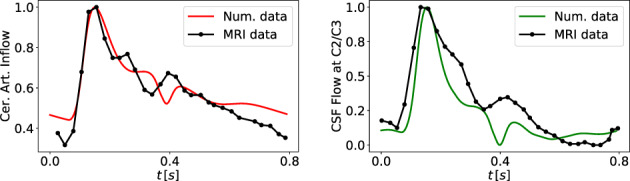
Computed results compared to measured data. Left frame: normalized cerebral arterial inflow (internal carotid arteries and vertebral arteries) over a cardiac cycle compared with MRI data from Reference 149. Right frame: normalized inflow of spinal subarachnoid space at C2/C3 level over a cardiac cycle compared with MRI data from Reference 149

**FIGURE 28:**
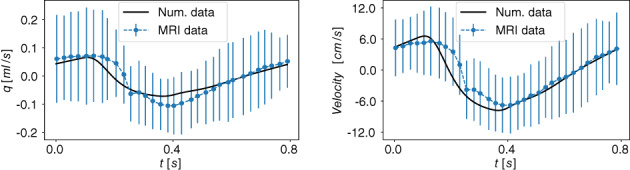
Computed flow and velocity in the aqueduct of Sylvius. Left frame: computed cerebrospinal fluid (CSF) flow through the aqueduct of Sylvius over a cardiac cycle compared with MRI data (mean and standard deviation) from Reference 138. Right frame: computed CSF velocity in the aqueduct of Sylvius over a cardiac cycle compared with MRI data (mean and standard deviation) from Reference 150

Figure 25 shows the time variation of the volumes occupied by different compartments of the cranio‐spinal system, stressing the effect of the Monro–Kellie hypothesis. During systole, intracranial arterial blood increases and arterial pulsations are transmitted directly into the incompressible CSF filled SAS. This evokes a chain of events in the following temporal order: CSF shifts out of the skull into the spinal canal; venous blood from the sinuses flows out of the brain mainly through the internal jugular veins and part of the CSF from the ventricles is displaced out through the AoS. Figure 26 underlines the relation between blood and CSF compartments' flow. In Figure 26 (left), arterial inflow, venous outflow through internal jugular veins, flow in AoS and inflow in SSAS are depicted during a cardiac cycle. In Figure 26 (right), the CSF and blood normalized flow of the same compartments is shown over a cardiac cycle. The lag in time between the systolic peaks is reported in Table 12. The arterio‐spinal CSF delay is underestimated by the mathematical model compared to the literature range, although the lag in time between arterial systolic peak and CSF peak in the AoS follows the literature data. This mismatch in the time lag of the flow of CSF into the spinal canal can be attributed to the lack of inertia of the model describing flow in the spinal canal.^147^ A 1D model for computing the flow of CSF within the SSAS under the simplifying assumption that it consists of two coaxial tubes representing the spinal cord and the dura^148^ could better capture the physiology and the interaction with other compartments. Despite this, the comparison of arterial cerebral inflow and inflow of the SSAS with MRI data from Reference 149, depicted in Figure 27, shows a good match between numerical results and physiological behavior. Finally, Figure 28 shows flow and CSF velocity through the AoS, featuring an oscillatory behavior with shape and amplitudes similar to those obtained from PC‐MRI flow quantification studies.^150^

**TABLE 12:** CSF and blood flow over a cardiac cycle

Index	Current value	Ref. value
Mean arterial flow [ml/s]	13.13	13.55 ± 3.07
Mean venous flow [ml/s]	12.15	9.42 ± 2.37
Mean CSF flow [ml/s]	0.01	0.08 ± 1.33
Mean AoS flow [ml/s]	0.01	0.03 ± 0.013
tIJV/tA [%]	92.52	71.1 ± 22
AV delay [% CC]	5.62	12.5 ± 8.06
Arterio‐CSF_*SSAS*_ delay [% CC]	0.625	5.35 ± 2.36
Arterio‐CSF_*AoS*_ delay [% CC]	28.12	22.1 ± 74.66

*Note*: Literature range taken from Reference 71. Arterial flow: flow in internal carotid arteries and vertebral arteries; Venous Flow: flow in internal jugular veins at C2C3 level; CSF flow: inflow of spinal subarachnoid space; tIJV/tA: ratio between total internal jugular veins flow and arterial flow at C2C3 level; AV, Arterio‐CSF_*SSAS*_, Arterio‐CSF_*AoS*_ Delay: lag in time between arterial and venous, spinal CSF and AoS CSF systolic peaks represented as a percentage of cardiac cycle.

## 6 APPLICATIONS: IMPACT OF HEAD AND NECK VENOUS STRICTURES ON BLOOD AND CSF DYNAMICS

In order to show the applicability of the presented model to situations of clinical relevance, we carry out a preliminary analysis of the effects of transverse sinus stenosis and of strictures in the main extra‐cranial venous outflow routes on the cerebral circulation, CSF and brain dynamics.

### 6.1 Idiopathic intracranial hypertension patient with transverse sinus stenoses

The role of vascular abnormalities in the onset and course of neurological diseases has long been recognized. In the last decade, the influence of intra‐ and extra‐cranial venous pathology as a trigger/cause of certain neurological disorders has gained attention. IIH is a neurological condition of unknown etiology, which requires prompt diagnosis and if left untreated can result in a rapidly progressive visual loss. As said before, how increased CSF pressure in the subarachnoid space influences intracranial arterial and venous fluid dynamics within the framework of the Monroe‐Kellie hypothesis remains unclear. Bateman showed that in IIH, the total cerebral arterial inflow is increased by 21%.^69^ On the venous front, there is evidence that almost 93% of patients with IIH harbor some degree of dural sinus stenosis.^139^

#### 6.1.1 Problem setup

**FIGURE 29:**
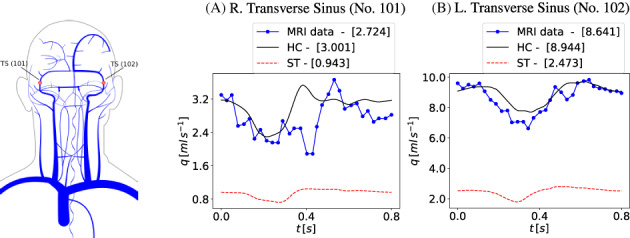
Location of transverse sinus stenosis and computed flow in healthy control (HC) and stenotic case (ST) compared with MRI data^59^

We investigate the impact of bilateral transverse sinus stenosis on cerebral venous flow and CSF dynamics, paying special attention to the role played by collateral flow pathways between deep cerebral vessels and extra‐cranial regions. We introduce stenoses to the reference venous network presented in Table 17 dividing the vessels affected by the stricture (the right and left transverse sinuses, No. 101 and 102, Figure 29) in two segments and putting between them a stenosis model. This model is based on References 152, 153 and it evaluates the flow variation in time across the stenosis by means of a first‐order ODE

(86) $$\frac{\mathrm{dq}\left(t\right)}{\mathrm{dt}}=\frac{1}{L}\left(\Delta p\left(t\right)-\mathrm{Rq}\left(t\right)-\mathrm{Bq}\left(t\right)|q\left(t\right)|\;\right).$$

where Δp is the difference between the upstream and downstream pressures and *L*, *R* and *B* are defined by

(87) $$L=\frac{k_{u}\rho l_{s}}{A_{0}},\;R=\frac{k_{v}\mu }{2r_{0}A_{0}},\;B=\frac{k_{t}\rho }{2A_{0}^{2}}{\left(\frac{A_{0}}{A_{s}}-1\right)}^{2}.$$

$A_{0}$ and $r_{0}$ are the mean reference area and radius of the vessels wherein the stenosis model is placed while $l_{s}$ and $A_{s}$ are the length and the minimum area of the stricture, taken equal to 1 cm and 10 $\%A_{0}$. Finally, $k_{u}=1.2$, $k_{t}=1.52$ and $k_{v}=16A_{0}^{2}/\left(r_{0}A_{s}^{2}\right)\left(0.83l_{s}+3.28r_{s}\right)$ are empirical coefficients.^152^, ^153^

#### 6.1.2 Comparison between healthy and IIH patient

**FIGURE 30:**
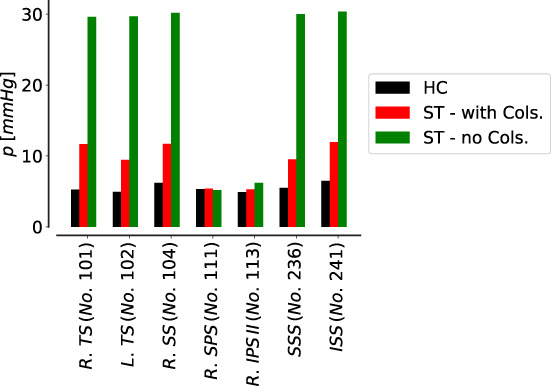
Averaged pressure over a cardiac cycle of different dural sinuses: comparison between healthy control (HC) and stenotic case (ST). HC: healthy control subject; ST—with Cols.: subject with transverse sinus stenosis and complete collateral circulation; ST—no Cols.: subject with transverse sinus stenosis without collateral circulation. TS: transverse sinus; SS: straight sinus; SPS: superior petrosal sinus; IPS: inferior petrosal sinus; SSS: superior sagittal sinus; ISS: inferior sagittal sinus

**FIGURE 31:**
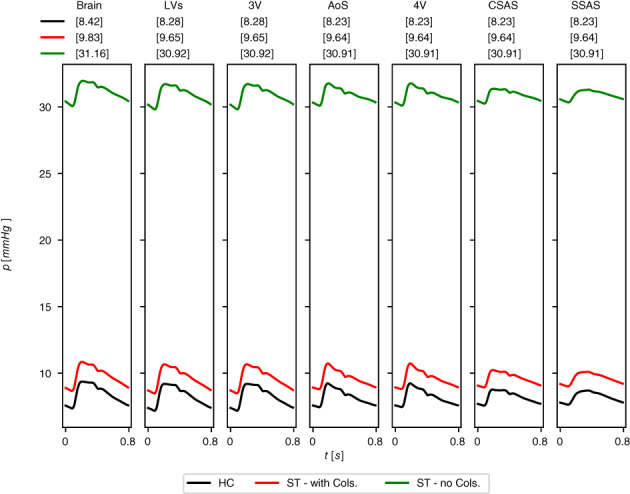
Pressure in CSF compartments over a cardiac cycle: comparison between healthy control (HC) and subject with transverse sinuses stenosis (ST). When the collateral routes are blocked, the intracranial pressure rises by 30 mmHg. HC: healthy control subject; ST—with Cols.: subject with transverse sinus stenosis and complete collateral circulation; ST—no Cols.: subject with transverse sinus stenosis without collateral circulation. Brain: fluid part of brain parenchyma; LVs: lateral ventricles; 3V: third ventricle; AoS: aqueduct of Sylvius; 4V: fourth ventricle; CSAS: cranial subarachnoid space; SSAS: spinal subarachnoid space

**FIGURE 32:**
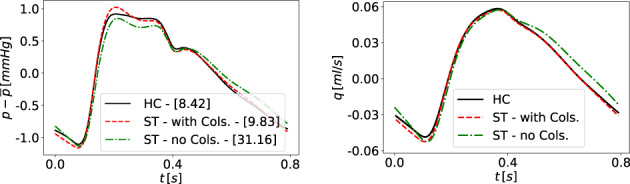
**(**Left frame) Intracranial pressure waveform: comparison between healthy control (HC) and subject with transverse sinuses stenosis (ST) with and without collateral circulation. We consider as intracranial pressure the pressure in the fluid part of the brain parenchyma. p: computed intracranial pressure, $\bar{p}$: averaged pressure over a cardiac cycle (value written in brackets). (Right frame) Variation in time of flow through the aqueduct of Sylvius for the healthy control (HC), a patient with transverse sinus stenosis with collaterals (ST—with Cols.) and a patient with transverse sinus stenosis without collaterals (ST—no Cols.). HC: healthy control subject; ST—with Cols.: subject with transverse sinus stenosis and complete collateral circulation; ST—no Cols.: subject with transverse sinus stenosis without collateral circulation

Figure 29 shows computed flow in the transverse sinuses for the healthy control (HC) and for the stenotic case (ST), along with MRI data for a healthy subject; there is a reduction of average flow rate of about 70%. Moreover, due to the stenosis, there is a re‐distribution of flow in dural sinuses and an increase in dural pressure (Figure 30). As a consequence, there is an increase in pressure in intracranial veins while the arterial flow and pressure are not modified. The venous hypertension due to transverse sinuses stenosis leads to decreased CSF reabsorption via arachnoid granulation which depends linearly on the pressure difference between intracranial pressure and superior sagittal sinus pressure. As a consequence, in order to maintain the balance between CSF generation and absorption, the intracranial pressure rises; moreover, following the Monro–Kellie hypothesis, a major amount of CSF is displaced into the spinal canal. For equal narrowing of the transverse sinuses, the severity of intracranial hypertension depends on the ability of the venous vascular network in developing collateral pathways to brain drainage. Occipital vein and sinus are the main collateral routes for flow limited by stenosis; the flow through these vessels increases significantly, in particular in the occipital vein (from 0.722 to 6.567 ml/s). We must consider that the venous network for head and neck used here represents a best‐case scenario, with all possible collaterals present. If the collateral circulation is impaired, the consequences of a stenosis in the dural sinuses should be aggravated. In order to explore this hypothesis, we performed a simulation where blood is forced to flow exclusively through the dural sinuses. Results are shown in Figure 31. There we show the computed intracranial pressure for the HC and for the patient with stenotic transverse sinuses, when the collateral routes are activated and also when they are compromised. In the first case, there is an increased intracranial pressure from 8.42 to 9.83 mmHg in brain parenchyma and a comparable increase in other intracranial compartments; on the other hand, when the collateral routes are excluded, the averaged intracranial pressure over the cardiac cycle rises from 8.42 to 31.16 mmHg in the brain. Concerning the intracranial pressure waveform, in Figure 32 (left) we observe that the pulse amplitude between the healthy subject and the one with transverse sinus stenosis does not change, both in case of complete collateral circulation and without collaterals. According to Reference 143, if the ICP values were low, the pulse wave presents a descending saw‐tooth appearance, with a clearly distinct P1 component; as the mean ICP rises, there is a progressive elevation in the magnitude of P2 and to a lesser extent of P3. Increase in the P2 component of the intracranial pressure wave is thought to represent decreased intracranial compliance.^140^, ^154^ Moreover, the increase in the P2 and P3 components of the ICP waveform may result from retrograde transmission of venous pressures to the CSF when there are changes in the cerebral venous system.^144^ From the numerical experiments shown here, we barely observe such changes in the ICP waveform. We attribute this fact to the linear character of intracranial compliant compartments. We consider it a limitation of our model in its current form, that will be addressed in future work.

Table 13 reports the cardiac cycle‐averaged cerebral arterial volume (1D arteries and arterioles), venous flow (capillaries, venules and 1D veins), cranial CSF and spinal CSF. Arterial volume is 28.98% of total blood volume; this data is in line with Reference 155 where the percentage of arterial blood volume with respect to total cerebral blood volume was estimated to be approximately 20–30%. When the collateral routes are blocked, the spinal CSF volume increases from 90.42 to 100.90 ml without significant changes in cerebral venous blood and cranial CSF. This rather insensitive behavior of venous blood volume is thought to be related to the modeling approach adopted for representing SR, which resistance to flow is not influenced by transmural pressure, see section 7 for more details. Figure 32 (right) shows flow through the AoS. As for the case of ICP, we observe small changes in flow pulsatility for all cases considered. This observation is not in line with experimental observations of flow across the AoS in patients with venous obstructions to cerebral blood drainage, where visible increase in flow pulsatility is observed.^150^, ^156^, ^157^ As already remarked for ICP, we believe that this behavior is due to the lack of non‐linear compliance in intracranial compartments.

**TABLE 13:** Computed averaged volume of blood and CSF over a cardiac cycle in main cerebral compartments

	HC	ST—with Cols.	ST—no Cols.
Arterial blood [ml]	48.01	48.00	48.05
Venous blood [ml]	117.84	118.12	119.83
Cranial CSF [ml]	469.55	469.86	471.53
Spinal CSF [ml]	90.42	91.18	100.90

*Note*: Arterial Blood: 1D arteries and arterioles; Venous Blood: capillaries, venules, 1D veins; Cranial CSF: lateral, third and fourth ventricles, aqueduct of Sylvius, cranial subarachnoid space and fluid part of the brain parenchyma; Spinal CSF: spinal subarachnoid space. Comparison between healthy control and stenotic cases. HC: healthy control; ST—with Cols.: stenotic case with collaterals; ST—no Cols.: stenotic case without collaterals.

Cerebral arterial inflow is similar between healthy and stenotic subjects with collaterals, 13.13 and 12.92 ml, showing that the cerebral autoregulation is maintaining the cerebral perfusion. The cerebral arterial pressure evaluated in the middle cerebral artery is 85.49 mmHg in healthy subject and 85.64 mmHg in the ST. However, the ratio between total jugular veins flow and arterial inflow is decreased from 93% in HC to 87% in subject with transverse sinuses stenosis; the outflow is deviated to external jugular veins and vertebral veins. When the collateral circulation is blocked in the ST, the arterial inflow is decreased to 10.11 ml and the ratio between total jugular veins flow and arterial inflow is 94%. The pressure in main cerebral arteries is increased by 3 mmHg. In this case, the CBF is significantly lower than that of HC, since the cerebral autoregulation is not able to fully compensate the drop in perfusion pressure (cerebral arterial pressure minus intracranial pressure) by reducing peripheral arterial resistances. According to Reference 63, standard MRI, MR venography and MR flow quantification studies revealed that the mean arterial inflow in stenotic patient with IIH is 21% above normal, but the SSS outflow was within the normal range; this means that the mean outflow as a percentage of the total inflow was reduced: this is evidence of collateral flow. In our mathematical model, we do not observe an increase in arterial inflow, both with and without collaterals circulation. However, when the collaterals are present, the percentage of venous outflow to arterial inflow is decreased, as in Reference 63. When the collateral circulation is reduced, the arterial inflow predicted by our model decreases. While this is the expected behavior if one analyses how our model is constructed, it contradicts observations in Reference 63. This disagreement could be due to the fact that we consider a sudden change from a baseline situation to a pathological one. There certainly are short‐ and/or long‐term mechanisms not considered in our model that produce the above mentioned experimental observation of increase cerebral flow in IIH patients. This aspect will be investigated in future work. Table 14 shows the flow coming in and out of the left and right brain parenchyma (considering the pressure driven and the constant flow) and the brain porosity, evaluated as the ratio between the volume of the fluid part of the brain parenchyma and its total volume. The flow through the brain parenchyma decreases when there are stenotic transverse sinuses with respect to the HC; when the collateral circulation is blocked, this decrease reaches 5%, that is almost 16% if we consider the pressure driven seepage of extracellular fluid flow from capillaries into the brain. The brain porosity remains almost constant in all cases.

**TABLE 14:** Cerebrospinal fluid exchange $q_{\mathrm{br},L-R}^{\text{in}}$ and $q_{\mathrm{br},L_{R}}^{\text{out}}$ and brain porosity $\Phi_{\mathrm{br}}$ (ratio between fluid part and total volume of brain parenchyma, considering a solid part of 980 ml): Comparison between healthy control and subject with transverse sinuses stenosis with and without collaterals

	HC	ST ‐ with Cols.	ST ‐ no Cols.
$q_{\mathrm{br},L}^{\text{in}}\times 10^{-4}$ [ml/s]	7.4	7.3 (−1.37)	7.0 (−5.4)
$q_{\mathrm{br},R}^{\text{in}}\times 10^{-4}$ [ml/s]	7.4	7.3 (−1.37)	7.0 (−5.4)
$q_{\mathrm{br},L}^{\text{out}}\times 10^{-4}$ [ml/s]	7.4	7.3 (−1.37)	7.0 (−5.4)
$q_{\mathrm{br},R}^{\text{out}}\times 10^{-4}$ [ml/s]	7.4	7.3 (−1.37)	7.0 (−5.4)
$\Phi_{\mathrm{br}}$	0.298	0.298	0.298

*Note*: Percentage variation with respect to healthy case in brackets. HC: healthy control; ST—with Cols.: stenotic case with collaterals; ST—no Cols.: stenotic case without collaterals.

### 6.2 Extracranial venous outflow strictures and their implication for Ménière's disease

Chronic cerebrospinal venous insufficiency (CCSVI) has been described as a chronic syndrome, characterized by extracranial venous malformations involving internal jugular veins, vertebral veins and the azygos vein.^12^ The narrowing of these veins hampers the normal outflow from the brain, causing an impact on intracranial haemodynamics, as well as on CSF and brain dynamics. Some works on modeling this condition are available in the literature. In References 69 and 158, stenoses of the internal jugular veins were studied, while Reference 84 concerns stenotic venous valves. In Reference 68, the neck venous strictures are associated to Ménière's disease, a pathology of the inner ear. These works reveal that CCSVI leads to a significant increase in intracranial pressure; however, since in previous versions of our work, a single CSF compartmental model was used, no refined information about the CSF dynamics could be obtained. Here, we investigate the impact on the CSF and brain dynamics resulting from the CCSVI condition, using a more sophisticated multicompartment model for CSF.

#### 6.2.1 Problem setup

We consider two different malformations of the extracranial venous vessels. The first one (case A) includes left and right stenotic internal jugular veins, symmetrically above the insertion of the middle thyroid vein, and also a stenosis in the azygos vein. In order to account for these strictures in the model, we introduce stenoses as represented in section 6.1.1 for vessels No. 224, 225, 244. The minimum area of the strictures is taken equal to 10% of the reference cross‐sectional area of the vessels where the stenosis model is placed (Table 2). As for transverse sinuses stenosis, this area, as well as the reference cross‐sectional area of the vessels, defines the parameters of the stenosis model in Equation (87). The second configuration (case B) takes into account stenotic valves, symmetric in both left and right internal jugular veins. The parameter $M_{s}$ of Equation (33) is taken equal to 0.25, causing an obstruction of 75% of the reference cross‐sectional area.

#### 6.2.2 Comparison between healthy and pathological patients

**FIGURE 33:**
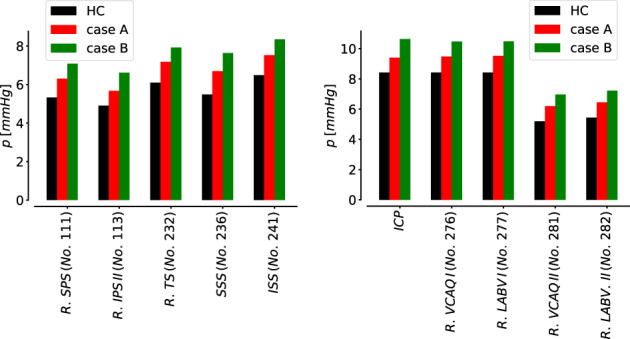
Computed cardiac‐cycle averaged pressures p for the healthy control (HC) and the CCSVI subjects with case 1 and case 2 with collateral circulation. (Left frame) Pressures p in main dural sinuses. SPS: superior petrosal sinus, IPS: inferior petrosal sinus; TS: transverse sinus; SSS: superior sagittal sinus; ISS: inferior sagittal sinus. (Right frame) Brain parenchyma pressure and results in main veins of the right inner ear. VCAQ: vein of the cochlear aqueduct; LABV: labyrinthine vein

**FIGURE 34:**
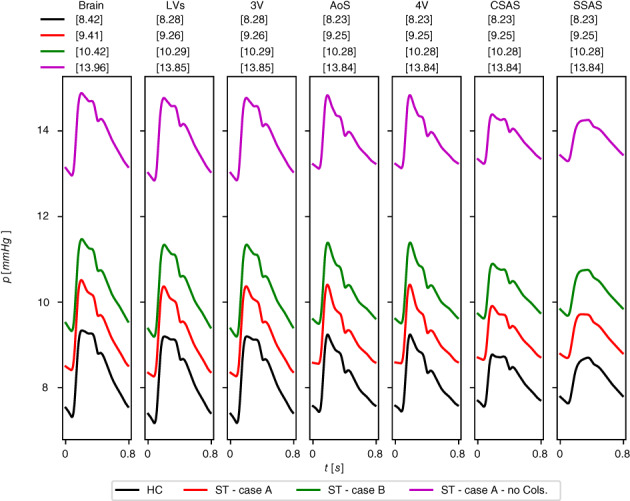
Computed pressure p in CSF compartments over a cardiac cycle: comparison between the healthy control (HC) and CCSVI patient (ST). Brain: fluid part of brain parenchyma; LVs: lateral ventricles; 3V: third ventricle; AoS: aqueduct of Sylvius; 4V: fourth ventricle; CSAS: cranial subarachnoid space; SSAS: spinal subarachnoid space

As expected, the CCSVI condition is related to a significant pressure drop across strictures. Pressure drops between the pre‐ and post‐stenotic locations are negligible in the case of the HC while in case A it is almost 1.37 mmHg and in case B it is 2.25 mmHg. The pressure rise observed in the extra‐cranial venous strictures is transmitted to the intracranial circulation. Figure 33 (left) depicts the computed cardiac‐cycle averaged pressures in the main dural sinuses of the venous network. Moreover, Figure 33 (right) shows averaged pressures for veins of the right inner ear. As reported in Reference 68, the venous pressure in the ear veins is increased due to the extra‐cranial venous stenoses; because of the functioning of the SR located in the ear circulation, this rise is not caused by the backward transmitted pressure waves from the obstructed sites, as occurs in dural sinuses, but it is due to the increased intracranial pressure. As shown in Figure 34, in all intracranial compartments the pressure is raised by 1 mmHg in case A and 2 mmHg in case B. By early animal experiments in Reference 159, it was proved that the subarachnoid space is linked to the endolymphatic space and the CSF pressure increase could be transmitted via the endolymphatic duct and sac to the inner ear, leading to the formation of the endolymphatic hydrops, one of the main anomalous conditions in Ménière's disease patients.

**FIGURE 35:**
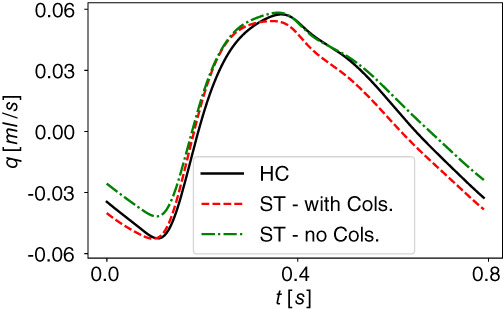
Variation in time of flow through the aqueduct of Sylvius for the healthy control (HC), the CCSVI patient with collaterals (ST—with Cols.) and the CCSVI patient without collaterals (ST—no Cols.)

As in the previous pathological setting, the ability of developing collateral routes for brain drainage are important in determining the severity of intracranial hypertension. When there are stenoses in the internal jugular veins, the flow is redirected to the extracranial jugular and vertebral veins. The arterial inflow and the ratio between internal jugular vein flow and arterial inflow are 13.11 ml and 76.31% for case A, while for case B they are 13.10 ml and 62.64%. When the collateral routes are blocked, the internal jugular veins are the main drainage alternatives. We simulated such a situation where collateral pathways are blocked; in this case the arterial inflow is 12.52 ml and the ratio between internal jugular veins outflow and arterial inflow is 99.4%. The intracranial pressure increases by 5.5 mmHg (see Figure 34). This raise in pressure is of the same order of magnitude of the pressure drop through the stenosis (10.13 mmHg above the stenosis and 4.48 mmHg below in the right internal jugular vein); in fact, the increase in venous pressure is transmitted up the vessels into the superior sagittal sinus (from an averaged pressure of 5.31 mmHg in the HC to 10.97 mmHg), causing a proportional reduction in CSF absorption and then an increase in intracranial pressure until equilibrium between CSF generation and absorption is reestablished.^160^ Table 15 reports data about the CSF flow in the brain parenchyma; due to the presence of stenosis, the pressure driven CSF seepage from the capillaries to the brain parenchyma is decreased. Figure 35 shows flow through the AoS. Deviations in terms of pulsatility are very small, even for the configuration featuring no collaterals. However, in Reference 150 changes in pulsatility were observed for patients with CCSVI. The fact that our model correctly captures the trend, that is, increased pulsatility, but fails to capture the magnitude of such increment, is certainly due to how well our model characterizes intracranial space compliance in the pathological case. Clearly some modeling aspects have to be improved, specifically the model of the SR (see discussion in next section), as well as mechanical properties of brain dynamics model compartments for patients with CCSVI.

**TABLE 15:** Cerebrospinal fluid exchange $q_{\mathrm{br},L}^{\text{in}}$, $q_{\mathrm{br},R}^{\text{in}}$, $q_{\mathrm{br},L}^{\text{out}}$, $q_{\mathrm{br},R}^{\text{out}}$ and brain porosity $\Phi_{\mathrm{br}}$ (ratio between fluid part and total volume of brain parenchyma, considering a solid part of 980 ml): Comparison of results for a healthy control and subject with IJV stenoses (case 1) with and without collaterals, and subject with IJV stenotic valves (case 2)

	HC	ST ‐ case 1 ‐ with Cols.	ST ‐ case 1 ‐ no Cols.	ST ‐ case 2 ‐ with Cols.
$q_{\mathrm{br},L}^{\text{in}}$ [ml/s]	0.00074	0.00074 (−0.84)	0.00073 (−2.05)	0.00073 (−1.75)
$q_{\mathrm{br},R}^{\text{in}}$ [ml/s]	0.00074	0.00074 (−0.84)	0.00073 (−2.05)	0.00073 (−1.75)
$q_{\mathrm{br},L}^{\text{out}}$ [ml/s]	0.00074	0.00074 (−0.84)	0.00073 (−2.05)	0.00073 (−1.75)
$q_{\mathrm{br},R}^{\text{out}}$ [ml/s]	0.00074	0.00074 (−0.84)	0.00073 (−2.05)	0.00073 (−1.75)
$\Phi_{\mathrm{br}}$	0.298	0.298	0.298	0.298

*Note*: Percentage variation with respect to healthy case in brackets. HC: healthy control; ST—with Cols.: stenotic case with collaterals; ST—no Cols.: stenotic case without collaterals.

## 7 CONCLUDING REMARKS

In this paper, we have presented a global, closed‐loop, multiscale model of the human circulation coupled with a multi‐compartmental model for the cerebrospinal‐fluid dynamics. The model comprises 1D descriptions for medium to large blood vessels, arteries and veins, accounting for the viscoelastic property of the blood vessel wall. Lumped‐parameter descriptions are used for other components of the full model that include the heart, the pulmonary circulation, the microvasculature, venous valves, SR and the dynamics of CSF in the craniospinal cavity, along with cerebral autoregulation. The present work departs from the Müller–Toro mathematical model^59^, ^69^ for the global systemic and pulmonary circulations in the entire human body. The main improvements with respect to the original Müller‐Toro model and other works fall into three categories: (a) mathematical models for a better description of the physiology of the circulatory system; (b) the computational methods used to solve the governing equations and (c) the enlarged range of potential applications of the resulting model.

On the physiological aspects of the present paper, the improvements include the adoption of a viscoelastic tube law, not just for the arterial tree as in References 39, 61, 134 but also for the entire venous circulation. We find that computed pressures and flow waveforms for the arterial and venous circulations are more realistic for the viscoelastic tube laws, than for the purely elastic case. The heart model in the present paper is also an improvement over that in the original global model,^59^, ^69^ the cardiac valves are represented through a model based on Reference 41. A novelty of the present paper is the coupling of the blood circulation to a refined mathematical description of the CSF dynamics in the craniospinal cavity.^62^ The CSF model comprises the cerebral ventricles, the AoS, the cranial and SSASs and the brain parenchyma. Other additions include the parametrization of the vascular beds, which together with the cerebral autoregulation model, is relevant when studying anatomical malformations of the cerebral circulation. The computational results for the arterial and venous circulation are compared with literature data, while MRI measurements are used for assessing our results for the cerebral venous circulation. Our results are in good agreement with literature and MRI data.

The physiology modeling improvements have resulted in new mathematical problems to be solved, notably, the viscoelastic nature of all major blood vessels. The associated parabolic system of equations has been approximated by a hyperbolic system with stiff source terms following a relaxation approach.^70^, ^78^, ^79^ The resulting stiff system is solved numerically with the same high‐order ADER‐type numerical scheme,^71^, ^95^ as in the originalmodel.^59^, ^69^ An additional numerical improvement is the adoption of the LTS technique,^106^ first introduced for blood flow in Reference 110 for solving a simplified 1D vessel network. This technique results in significant computational savings, which are more evident when coupling the blood circulation to the CSF and brain dynamics, as thesetwo systems have different temporal scales and the computational time needed to reach periodicity of the solution is considerably larger than the time scale of a cardiac cycle.

The model as presented is applicable to many pathophysiological conditions associated to the circulatory system, involving both the arterial and the venous systems. In the present paper, we have placed considerable emphasis on the CNS fluids in the craniospinal cavity, in which blood (arteries, microvasculature and veins) interact with the CNS fluids. To illustrate the applicability of the present model that couples the blood circulation and the CSF dynamics in a holistic setting, we have presented two specific medical applications, namely IIH as associated to transverse sinus stenoses, and Ménière's disease as associated to extracranial venous outflow strictures. Our results reveal that obstructions in the cerebral venous network lead to intracranial hypertension and disruption of the fluid dynamics in the entire craniospinal cavity. The severity of the consequences of intracranial or extracranial venous outflow obstructions depends on the balance between CSF generation and absorption, the displacement of CSF into the spinal cord and the ability of the venous network in developing collateral routes to respond to the venous outflow obstructions. These findings are relevant to the study of a very important function of CNS fluids, namelythe clearance of brain metabolic waste and neurotoxins from the CNS. Impairment of the cerebral venous drainage will directly disrupt this clearance function. Indirectly, CSF absorption into the venous system will be hampered, due to venous hypertension, leading to decreased CSF turnover, which will also affect the clearance function.

In spite of the progress reported in this paper, there are several limitations to be addressed in future developments. One limitation is the description of SR, which are major determinants of cerebral venous dynamics; blood flow through these compliant vessels is controlled by sphincter‐like structures, which regulate discharge into the main dural sinuses. Another limitation is the absence of a model for solute transport. This limitation prevent us at present from properly describing, via Starling forces,^2^ the transport of fluid and selected solutes across the blood–brain barrier (BBB), for example. Overcoming this limitation will be crucial for tackling the brain waste clearance function of the CNS, alluded to earlier. Mathematical modeling steps in this direction are outlined in References 161, 162. Another limitation involves the linear distenibility equations of the CSF model. Following Reference 62, these equations link the internal pressure with the cross‐sectional area of the compartment in a linear manner. A simple linear pressure‐volume relationship is acceptable in the physiological pressure range, but could give under‐ and/or over‐estimation of pressure changes in case of large volume changes, especially when addressing pathological conditions. Future work will address the non‐linear behavior of the pressure‐volume relationship in the CSF compartments. Another potential improvement concerns the representation of CSF in the spinal canal, which at present consists of a single 0D model; a possible improvement could be the adoption of a 1D model, as proposed in Reference 148, which is based on two coaxial compliant tubes representing the spinal cord and the CSF between the cord and the dura. Potentially, such representation admitting spatial variations could provide the bases for adding new potential routes for CSF reabsorption.

Addressing the modeling of the microcirculation is a challenging task. Here, the microcirculation was simplified as a lumped resistor capacitor system. While this simplification gave acceptable system‐wide predictions, it is not able to account for biphasic blood flow phenomena^163^ and network effects^164^ that occur in the microcirculation. Significant progress has been made to develop realistic microvascular networks models^135^, ^165^, ^166^ which could be integrated with the proposed system models in future work.

On balance, the mathematical model presented here is a significant improvement of the original model^59^, ^69^ published 7 years ago, represents the current state of the art and could provide the bases for realistic applications that require the representation of all extracellular body fluids in a holistic setting, along with regulatory processes.

## 1

Tables A1 and A2 report geometrical data for arteries and veins networks. The first columns refer to number, name, length and radii of each vessel while the last one show the location of the vessel in the human body (using the same location codes of Reference 59, 1 = Dural sinuses, 2 = Extracranial, 3 = Neck, 4 = Thorax, 5 = Abdomen, 6 = Upper limbs, 7 = Lower limbs, 8 = Pelvis, 9 = Intracranial). Geometrical data are the same of References 59, 74, 75 while, for vessels added or changed in this work, the geometry is estimated from literature data.^111^, ^167^

**TABLE A1:** Geometrical and mechanical parameters of the arterial network. *R* ^*T*^ is given in mmHg s/ml while *C*_*art*_ in ml (s mmHg)^−1^

No.	Vessel name	*L* (cm)	$r_{0}$ (cm)	$r_{1}$ (cm)	$R^{T}$	$C_{\mathrm{art}}$	Loc
1	Ascending aorta	2	1.525	1.42	‐	‐	4
2	Aortic arch	3	1.42	1.342	‐	‐	4
3	Brachiocephalic a.	3.5	0.65	0.62	‐	‐	4
4	R. subclavian a.	3.5	0.425	0.407	‐	‐	6
5	R. carotid a.	17.7	0.4	0.37	‐	‐	3
6	R. vertebral a.	13.5	0.15	0.136	‐	‐	3
7	R. subclavian a.	39.8	0.407	0.23	‐	‐	6
8	R. radius	22	0.175	0.14	40.3876	0.0059	6
9	R. ulnar a.	6.7	0.215	0.215	‐	‐	6
10	Aortic arch	4	1.342	1.246	‐	‐	4
11	L. carotid a.	20.8	0.4	0.37	‐	‐	3
12	Thoracic aorta	5.5	1.246	1.124	‐	‐	4
13	Thoracic aorta	10.5	1.124	0.924	‐	‐	4
14	Intercostal a.	7.3	0.3	0.3	10.7761	0.0586	4
15	L. subclavian a.	3.5	0.425	0.407	‐	‐	6
16	L. vertebral a.	13.5	0.15	0.136	‐	‐	3
17	L. subclavian a.	39.8	0.407	0.23	‐	‐	6
18	L. ulnar a.	6.7	0.215	0.215	‐	‐	6
19	L. radius	22	0.175	0.14	40.8837	0.0059	6
20	Celiac a.	2	0.35	0.3	‐	‐	5
21	Celiac a.	2	0.3	0.25	‐	‐	5
22	Hepatic a.	6.5	0.275	0.25	27.8243	0.0089	5
23	Splenic a.	5.8	0.175	0.15	41.2558	0.0059	5
24	Gastric a.	5.5	0.2	0.2	18.0678	0.0139	5
25	Abdominal aorta	5.3	0.924	0.838	‐	‐	5
26	Sup. mesenteric a.	5	0.4	0.35	7.1584	0.0342	5
27	Abdominal aorta	1.5	0.838	0.814	‐	‐	5
28	R. renal a.	3	0.275	0.275	8.8134	0.0287	5
29	Abdominal aorta	1.5	0.814	0.792	‐	‐	5
30	L. renal a.	3	0.275	0.275	8.8134	0.0287	5
31	Abdominal aorta	12.5	0.792	0.627	‐	‐	5
32	Inf. mesenteric a.	3.8	0.2	0.175	52.3507	0.0075	5
33	Abdominal aorta	8	0.627	0.55	‐	‐	5
34	R. com. iliac a.	5.8	0.4	0.37	‐	‐	8
35	R. ext. iliac a.	14.5	0.37	0.314	‐	‐	8
36	R. int. iliac a.	4.5	0.2	0.2	42.8843	0.0077	8
37	R. deep femoral a.	11.3	0.2	0.2	25.902	0.0485	7
38	R. femoral a.	44.3	0.314	0.275	‐	‐	7
39	R. ext. carotid a.	4.1	0.2	0.15	‐	‐	2
40	L. int. carotid a.	17.6	0.25	0.2	‐	‐	3
41	R. post. tibial a.	34.4	0.175	0.175	57.3809	0.0211	7
42	R. ant. tibial a.	32.2	0.25	0.25	25.7857	0.0485	7
43	R. interosseous a.	7	0.1	0.1	644.368	0.0018	6
44	R. ulnar a.	17	0.203	0.18	40.9859	0.0059	6
45	L. ulnar a.	17	0.203	0.18	40.9859	0.0059	6
46	L. interosseous a.	7	0.1	0.1	644.368	0.0018	6
47	R. int. carotid a.	17.6	0.25	0.2	‐	‐	3
48	L. ext. carotid a.	4.1	0.2	0.15	‐	‐	3
49	L. com. iliac a.	5.8	0.4	0.37	‐	‐	8
50	L. ext. iliac a.	14.5	0.37	0.314	‐	‐	8
51	L. int. iliac a.	4.5	0.2	0.2	42.8843	0.0077	8
52	L. deep femoral a.	11.3	0.2	0.2	25.902	0.0485	7
53	L. femoral a.	44.3	0.314	0.275	‐	‐	7
54	L. post. tibial a.	34.4	0.175	0.175	57.3809	0.0211	7
55	L. ant. tibial a.	32.2	0.25	0.25	25.7857	0.0485	7
56	Basilar a.	0.96	0.162	0.162	‐	‐	1
57	R. post. cerebral. a.	0.5	0.107	0.107	‐	‐	1
58	R. post. cerebral. a.	8.6	0.105	0.105	46.3595	0.0029	1
59	R. post. communicating a.	1.5	0.073	0.073	‐	‐	1
60	R. int. carotid a.	0.5	0.2	0.2	‐	‐	1
61	R. mid. cerebral a.	11.9	0.143	0.143	22.5727	0.0059	1
62	R. ant. cerebral a.	1.2	0.117	0.117	‐	‐	1
63	R. ant. cererbal a.	10.3	0.12	0.12	45.0952	0.0029	1
64	Ant. communicating a.	0.3	0.1	0.1	‐	‐	1
65	L. ant. cerebral a.	10.3	0.12	0.12	45.0952	0.0029	1
66	L. ant. cerebral a.	1.2	0.117	0.117	‐	‐	1
67	L. mid. cerebral a.	11.9	0.143	0.143	22.5727	0.0059	1
68	L. int. carotid a.	0.5	0.2	0.2	‐	‐	1
69	L. post. communicating a.	1.5	0.073	0.073	‐	‐	1
70	L. post. cerebral a.	8.6	0.105	0.105	46.3595	0.0029	1
71	L. post. cerebral a.	0.5	0.107	0.107	‐	‐	1
72	L. ext. carotid a.	6.1	0.2	0.2	‐	‐	3
73	R. ext. carotid a.	6.1	0.2	0.2	‐	‐	3
74	L. sup. thyroid a.	10.1	0.1	0.1	228.82	0.0016	3
75	R. sup. thyroid a.	10.1	0.1	0.1	228.82	0.0016	3
76	L. superf. temporal a.	6.1	0.16	0.16	‐	‐	2
77	R. superf. temporal a.	6.1	0.16	0.16	‐	‐	2
78	L. maxillary a.	9.1	0.11	0.11	190.278	0.0016	2
79	R. maxillary a.	9.1	0.11	0.11	190.278	0.0016	2
80	L. superf. temp. fron. bran.	10	0.11	0.11	11379.1	0.0016	2
81	R. superf. temp. fron. bran.	10	0.11	0.11	190.278	0.0016	2
82	L. superf. temp. pari. bran.	10.1	0.11	0.11	190.278	0.0016	2
83	R. superf. temp. pari. bran	10.1	0.11	0.11	11379.1	0.0016	2
169	R. facial a.	11.6	0.13	0.13	207.502	0.0063	2
170	L. facial a.	11.6	0.13	0.13	207.502	0.0063	2
274	Basilar a. II	0.386	0.162	0.162	‐	‐	1
275	R. Labyrinthine artery	1	0.01	0.01	377,411	0.0006	1
278	L. Labyrinthine artery	1	0.01	0.01	377,421	0.0006	1
285	R. PICA I	1	0.0433	0.0433	‐	‐	1
286	L. PICA I	1	0.0433	0.0433	‐	‐	1
287	R. SCA	1	0.0558	0.0558	111.364	0.0006	1
288	L. SCA	1	0.0558	0.0558	111.368	0.0006	1
293	L. Vertebral artery II	0.75	0.15	0.136	‐	‐	3
294	R. Vertebral artery II	0.75	0.15	0.136	‐	‐	3
304	R. AICA I	1.4	0.0366	0.05	‐	‐	1
305	L. AICA I	1.4	0.0366	0.05	‐	‐	1
306	R. AICA II	2.35	0.0366	0.05	468.13	0.0006	1
307	L. AICA II	2.35	0.0366	0.05	468.23	0.0006	1
308	R. PICA MB	1	0.0216	0.0216	17099.13	0.0006	1
309	L. PICA MB	1	0.0216	0.0216	17099.13	0.0006	1
310	R. PICA II	1	0.0433	0.0433	646.13	0.0006	1
311	L. PICA II	1	0.0433	0.0433	646.13	0.0006	1
312	R. ASA	1	0.048	0.048	1509.03	0.0006	1
313	L. ASA	1	0.048	0.048	1509.03	0.0006	1
314	R. VA III	0.75	0.15	0.136	‐	‐	3
315	L. VA III	0.75	0.15	0.136	‐	‐	3
316	R. pontine a. I	1	0.012	0.012	91716.93	0.0006	1
317	R. pontine artery II	1	0.012	0.012	91596.94	0.0006	1
318	L. pontine a. I	1	0.012	0.012	91716.93	0.0006	1
319	L. pontine artery II	1	0.012	0.012	91596.94	0.0006	1
320	BA III	0.386	0.162	0.162	‐	‐	1
321	BA IV	0.386	0.162	0.162	‐	‐	1
322	BA V	0.386	0.162	0.162	‐	‐	1
323	BA VI	0.386	0.162	0.162	‐	‐	1

**TABLE A2:** Geometrical and mechanical parameters of the venous network. *R*_*vn*_ is given in mmHg s/ml while *C*_*vn*_ in ml (s mmHg)^−1^

No.	Vessel name	*L* (cm)	$r_{0}$ (cm)	$r_{1}$ (cm)	$R_{\mathrm{vn}}$	*C* $C_{vn}^{}$	Loc
84	Sup. vena cava	1.5	0.8	0.8	‐	‐	4
85	Sup. vena cava	2	0.8	0.8	‐	‐	4
86	R. brachiocephalic v.	4	0.564	0.564	‐	‐	4
87	L. brachiocephalic v.	7.5	0.535	0.535	‐	‐	4
88	L. subclavian v. I	3	0.564	0.564	‐	‐	6
89	R. subclavian v. I	3	0.564	0.564	‐	‐	6
90	R. ext. jugular v.	10	0.252	0.252	‐	‐	3
91	L. ext. jugular v.	10	0.252	0.304	‐	‐	3
92	R. int. jugular v.	2.5	0.399	0.399	‐	‐	3
93	L. int. jugular v.	2.5	0.564	0.618	‐	‐	3
94	L. vertebral v.	11	0.138	0.16	‐	‐	3
95	R. vertebral v.	11	0.138	0.16	‐	‐	3
96	R. deep cervical v.	13	0.16	0.16	‐	‐	3
97	L. deep cervical v.	13	0.16	0.16	‐	‐	3
98	Vertebral venous plexus	71	0.368	0.368	‐	‐	3
99	R. sigmoid sinus	3.5	0.252	0.252	‐	‐	1
100	L. sigmoid sinus	3.5	0.357	0.378	‐	‐	1
101	R. trans. sinus	3.5	0.178	0.252	‐	‐	1
102	L. trans. sinus	3.5	0.309	0.357	‐	‐	1
103	Sup. sagittal sinus	2.5	0.35	0.367	‐	‐	1
104	Straight sinus	4	0.25	0.25	‐	‐	1
105	Inf. sagittal sinus	3.67	0.16	0.16	‐	‐	1
106	Vein of Galen	0.6	0.366	0.4	‐	‐	9
107	L. int. cerebral v.	5	0.126	0.126	5.369	0.0539	9
108	R. int. cerebral v.	5	0.126	0.126	5.369	0.0539	9
109	L. basal v. of Rosenthal	1	0.126	0.126	5.369	0.0539	9
110	R. basal v. of Rosenthal	1	0.126	0.126	5.369	0.0539	9
111	R. sup. petrosal sinus	3.7	0.149	0.149	‐	‐	1
112	L. sup. petrosal sinus	3.7	0.149	0.149	‐	‐	1
113	R. inf. petrosal sinus	3.2	0.08	0.16	‐	‐	1
114	L. inf. petrosal sinus	3.2	0.08	0.16	‐	‐	1
115	R. post. auricular v.	5	0.08	0.08	15.817	0.0119	2
116	L. post. auricular v.	5	0.08	0.08	15.817	0.0119	2
117	R. post. retromandibular v.	3.52	0.25	0.25	‐	‐	2
118	L. post. retromandibular v.	3.52	0.25	0.25	‐	‐	2
119	R. ant. retromandibular v.	3.15	0.235	0.235	‐	‐	2
120	L. ant. retromandibular v.	3.15	0.235	0.235	‐	‐	2
121	R. retromandibular v.	4.5	0.26	0.26	‐	‐	2
122	L. retromandibular v.	4.5	0.26	0.26	‐	‐	2
123	R. facial v.	6	0.132	0.178	‐	‐	2
124	L. facial v.	6	0.132	0.178	‐	‐	2
125	R. com. facial v.	0.9	0.18	0.18	‐	‐	2
126	L. com. facial v.	0.9	0.18	0.18	‐	‐	2
127	R. superf. temp. v.	5	0.19	0.19	2.119	0.5042	2
128	L. superf. temp. v.	5	0.19	0.19	2.119	0.5042	2
129	R. maxillary v.	1	0.175	0.175	‐	‐	2
130	L. maxillary v.	1	0.175	0.175	‐	‐	2
131	R. deep facial v.	0.9	0.25	0.25	‐	‐	2
132	L. deep facial v.	0.9	0.25	0.25	‐	‐	2
133	R. emissary v.	3	0.1	0.1	‐	‐	2
134	L. emissary v.	3	0.1	0.1	‐	‐	2
135	R. pterygoid plexus	0.9	0.15	0.15	‐	‐	2
136	L. pterygoid plexus	0.9	0.15	0.15	‐	‐	2
137	R. marginal sinus	4	0.1	0.1	‐	‐	1
138	L. marginal sinus	4	0.1	0.1	‐	‐	1
139	Occipittal sinus	3.5	0.235	0.235	‐	‐	1
140	R. ext. jugular v.	10	0.252	0.252	‐	‐	3
141	R. mastoid emissary v.	7.2	0.175	0.175	‐	‐	2
142	L. mastoid emissary v.	7.2	0.175	0.175	‐	‐	2
143	R. post. condylar v.	3	0.315	0.315	‐	‐	2
144	L. post. condylar v.	3	0.315	0.315	‐	‐	2
145	R. subocc. sinus	1	0.45	0.45	‐	‐	2
146	R. lat. ant. condylar v.	3	0.315	0.315	‐	‐	2
147	L. lat. ant. condylar v.	3	0.315	0.315	‐	‐	2
148	L. ext. jugular v.	10	0.304	0.357	‐	‐	3
149	Sup. sagittal sinus	4.33	0.229	0.258	‐	‐	1
150	R. Labbe v.	5	0.15	0.15	3.622	0.0539	9
151	L. Labbe v.	5	0.15	0.15	3.622	0.0539	9
152	Sup. sagittal sinus	4.33	0.258	0.287	‐	‐	1
153	Sup. sagittal sinus	2.5	0.334	0.35	‐	‐	1
154	L. cavernous sinus	1.5	0.1	0.1	‐	‐	1
155	R. cavernous sinus	1.5	0.1	0.1	‐	‐	1
156	Occipittal v.	5	0.126	0.126	‐	‐	2
157	Sup. sagittal sinus	5	0.3	0.334	‐	‐	1
158	Cerebral vein	5	0.15	0.15	3.622	0.0207	9
159	Cerebral vein	5	0.15	0.15	3.622	0.0207	9
160	Azygos v.	2	0.425	0.425	‐	‐	4
161	Cerebral vein	5	0.15	0.15	3.622	0.0207	9
162	Cerebral vein	5	0.15	0.15	3.622	0.0207	9
163	R. vertebral v.	5	0.16	0.16	‐	‐	3
164	L. vertebral v.	5	0.16	0.16	‐	‐	3
165	Sup. sagittal sinus	4.33	0.2	0.229	‐	‐	1
166	L. subocc. sinus	1	0.45	0.45	‐	‐	2
167	R. anastomotic v.	2	0.1	0.1	‐	‐	3
168	L. anastomotic v.	2	0.1	0.1	‐	‐	3
171	R. great saphenous v.	7.5	0.2215	0.23	‐	‐	7
172	L. great saphenous v.	7.5	0.2215	0.23	‐	‐	7
173	L. post. tibial v.	17.3	0.15	0.15	‐	‐	7
174	L. ant. tibial v.	16	0.15	0.15	‐	‐	7
175	R. popliteal v.	19	0.34	0.34	‐	‐	7
176	L. popliteal v.	19	0.34	0.34	‐	‐	7
177	L. femoral v.	25.4	0.35	0.35	‐	‐	7
178	R. femoral v.	25.4	0.35	0.35	‐	‐	7
179	R. deep femoral v.	12.6	0.35	0.35	0.51	2.8741	7
180	L. deep femoral v.	12.6	0.35	0.35	0.51	2.8741	7
181	R. ext. iliac v.	14.4	0.5	0.5	‐	‐	8
182	L. ext. iliac v.	14.4	0.5	0.5	‐	‐	8
183	L. int. iliac v.	5	0.15	0.15	3.622	3.5692	8
184	R. int. iliac v.	5	0.15	0.15	3.622	3.5692	8
185	R. com. iliac v.	2	0.575	0.575	‐	‐	8
186	L. com. iliac v.	2	0.575	0.575	‐	‐	8
187	R. radial v.	40.6	0.2	0.2	1.885	0.651448	6
188	L. interosseous v.	7	0.1	0.1	9.069	0.0372	6
189	R. ulnar v.	30.6	0.2	0.2	1.885	0.6514	6
190	L. ulnar v.	30.6	0.2	0.2	1.885	0.6514	6
191	L. interosseous v.	7	0.1	0.1	9.069	0.0372	6
192	L. radial v.	40.6	0.2	0.2	1.885	0.6514	6
193	L. subclavian v.	27	0.52	0.52	‐	‐	6
194	R. subclavian v.	27	0.52	0.52	‐	‐	6
195	L. subclavian v.	3	0.52	0.52	‐	‐	6
196	R. subclavian v.	3	0.52	0.52	‐	‐	6
197	L. ulnar v.	10	0.2	0.2	‐	‐	6
198	Inf. vena cava	15.3	0.7625	0.7625	‐	‐	5
199	Hepatic v.	6.8	0.485	0.485	0.229	82.3232	5
200	Inf. vena cava	1.5	0.7625	0.7625	‐	‐	5
201	inf. vena cava	1.5	0.7625	0.7625	‐	‐	5
202	Inf. vena cava	12.5	0.7625	0.7625	‐	‐	5
203	Inf. vena cava	8	0.7625	0.7625	‐	‐	5
204	R. com. iliac v.	3.8	0.575	0.575	‐	‐	8
205	L. com. iliac v.	3.8	0.575	0.575	‐	‐	8
206	R. ulnar v.	10	0.2	0.2	‐	‐	6
207	L. renal v.	3.2	0.25	0.25	1.128	11.6388	5
208	R. renal v.	3.2	0.25	0.25	1.128	11.6388	5
209	Ascending lumbar v.	23	0.2	0.2	‐	‐	5
210	hemiazygos v.	23	0.28	0.28	‐	‐	5
211	Inf. mesenteric v.	6	0.45	0.45	0.276	4.5795	5
212	R. post. tibial v.	17.3	0.15	0.15	‐	‐	7
213	R. ant. tibial v.	16	0.15	0.15	‐	‐	7
214	R. ant. tibial v.	2	0.6	0.6	0.132	0.5787	7
215	L. ant. tibial v.	2	0.6	0.6	0.132	0.5787	7
216	R. lumbar v.	3.8	0.1	0.1	‐	‐	5
217	L. lumbar v.	3.8	0.1	0.1	‐	‐	5
218	R. sup. thyroid v.	4	0.15	0.15	‐	‐	3
219	L. sup. thyroid v.	4	0.15	0.15	‐	‐	3
220	R. mid. thyroid v.	3	0.1	0.1	‐	‐	3
221	L. mid. thyroid v.	3	0.1	0.1	‐	‐	3
222	Inf. thyroid v.	7	0.126	0.126	‐	‐	3
223	Thyroid connection	2	0.16	0.16	3.131	0.2585	3
224	R. int. jugular v.	3	0.357	0.357	‐	‐	3
225	L. int. jugular v.	3	0.564	0.564	‐	‐	3
226	R. int. jugular v.	2.7	0.252	0.357	‐	‐	3
227	L. int. jugular v.	2.7	0.564	0.564	‐	‐	3
228	R. int. jugular v.	6.8	0.252	0.252	‐	‐	3
229	L. int. jugular v.	6.8	0.399	0.564	‐	‐	3
230	R. sigmoid sinus	1.5	0.252	0.252	‐	‐	1
231	L. sigmoid sinus	1.5	0.378	0.399	‐	‐	1
232	R. trans. sinus	3.5	0.218	0.178	‐	‐	1
233	L. trans. sinus	3.5	0.437	0.309	‐	‐	1
234	R. facial v.	2	0.113	0.132	6.256	0.284	2
235	L. facial v.	2	0.113	0.132	6.256	0.284	2
236	Sup. sagittal sinus	2	0.287	0.3	‐	‐	1
237	Cerebral vein	5	0.15	0.15	3.622	0.1652	9
238	Cerebral vein	5	0.15	0.15	3.622	0.1652	9
239	Cerebral vein	5	0.15	0.15	3.622	0.1652	9
240	Intra‐cavernous sinus	2	0.126	0.126	‐	‐	1
241	Inf. sagittal sinus	3.67	0.16	0.16	‐	‐	1
242	R. int. jugular v.	1	0.399	0.399	‐	‐	3
243	L. int. jugular v.	1	0.618	0.618	‐	‐	3
244	Azygos v.	28	0.425	0.425	‐	‐	4
245	Cerebral vein	3	0.15	0.15	3.622	0.1652	9
246	L. basal v. of Rosenthal	7	0.126	0.126	‐	‐	9
247	R. basal v. of Rosenthal	7	0.126	0.126	‐	‐	9
248	Inf. sagittal sinus	3.67	0.16	0.16	‐	‐	1
249	Cerebral vein	3	0.15	0.15	3.622	0.1652	9
250	Intercostal v.	2	0.4	0.4	0.369	1.395	4
251	R. post. tibial v.	17.3	0.15	0.15	3.622	0.6805	7
252	R. ant. tibial v.	16	0.15	0.15	‐	‐	7
253	R. great saphenous v.	37.5	0.145	0.1875	‐	‐	7
254	L. great saphenous v.	37.5	0.145	0.1875	‐	‐	7
255	L. ant. tibial v.	16	0.15	0.15	‐	‐	7
256	L. post. tibial v.	17.3	0.15	0.15	3.622	0.6805	7
257	R. great saphenous v.	30	0.1875	0.2215	‐	‐	7
258	L. great saphenous v.	30	0.1875	0.2215	‐	‐	7
259	Confluence of sinuses	1	0.1	0.1	‐	‐	1
260	Cerebral vein	3	0.15	0.15	3.622	0.1652	9
261	Terminal cerebral vein	1	0.15	0.15	‐	‐	1
262	Terminal cerebral vein	1	0.15	0.15	‐	‐	1
263	Terminal cerebral vein	1	0.15	0.15	‐	‐	1
264	Terminal cerebral vein	1	0.15	0.15	‐	‐	1
265	Terminal cerebral vein	1	0.15	0.15	‐	‐	1
266	Terminal cerebral vein	1	0.15	0.15	‐	‐	1
267	Terminal cerebral vein	1	0.15	0.15	‐	‐	1
268	Terminal cerebral vein	1	0.15	0.15	‐	‐	1
269	Terminal cerebral vein	1	0.15	0.15	‐	‐	1
270	Terminal cerebral vein	1	0.15	0.15	‐	‐	1
271	Terminal cerebral vein	1	0.309	0.366	‐	‐	9
272	Terminal cerebral vein	1	0.15	0.15	‐	‐	1
273	Terminal cerebral vein	1	0.15	0.15	‐	‐	1
276	R. v. of cochlear aq. I	0.65	0.01	0.01	253.077	2.33E‐05	9
277	R. labyrinthine v. I	0.43	0.037	0.037	71.986	2.33E‐05	9
279	L. v. of cochlear aq. I	0.65	0.01	0.01	253.077	2.33E‐05	9
280	L. labyrinthine v. I	0.43	0.037	0.037	17.997	2.33E‐05	9
281	R. v. of cochlear aq. II	0.65	0.01	0.01	‐	‐	1
282	R. labyrinthine v. II	0.43	0.037	0.037	‐	‐	1
283	L. v. of cochlear aq. II	0.65	0.01	0.01	‐	‐	1
284	L. labyrinthine v. II	0.43	0.037	0.037	‐	‐	1
289	R. Sup. vermian vein	1	0.08	0.08	15.817	0.006	9
290	L. Sup. vermian vein	1	0.08	0.08	15.817	0.006	9
291	R. Inf. vermian vein	1	0.09	0.09	11.577	0.008	9
292	L. Inf. vermian vein	1	0.09	0.09	11.577	0.008	9
295	R. Inf. vermian vein II	1	0.09	0.09	‐	‐	1
296	L. Inf. vermian vein II	1	0.09	0.09	‐	‐	1
297	Cerebral vein	1	0.309	0.36	‐	‐	9
298	R. Sup. petrosal vein	1	0.08	0.08	15.817	0.008	9
299	L. Sup. petrosal vein	1	0.08	0.08	15.817	0.008	9
300	R. Sup. petrosal vein II	1	0.08	0.08	‐	‐	1
301	L. Sup. petrosal vein II	1	0.08	0.08	‐	‐	1
302	R. Sup. petrosal sinus II	2	0.149	0.149	‐	‐	1
303	L. Sup. petrosal sinus II	2	0.149	0.149	‐	‐	1
